# Revision of New World Species of the Shore-fly Subgenus *Allotrichoma* Becker of the Genus *Allotrichoma* with Description of the Subgenus *Neotrichoma* (Diptera, Ephydridae, Hecamedini)

**DOI:** 10.3897/zookeys.161.2016

**Published:** 2012-01-04

**Authors:** Wayne N. Mathis, Tadeusz Zatwarnicki

**Affiliations:** 1 Department of Entomology, PO Box 37012, MRC 169; Smithsonian Institution, Washington, D.C. 20013-7012, USA; 2 Department of Biosystematics, Opole University, ul. Oleska 22, 45-052 Opole, Poland

**Keywords:** Diptera, Ephydridae (Hecamedini), *Allotrichoma*, New World, new species

## Abstract

The New World species of the subgenera *Allotrichoma* Becker and *Neotrichoma* (new subgenus) are revised, including a phylogenetic analysis of the species groups and subgenera within the genus *Allotrichoma*. For phylogenetic perspective and to document the monophyly of the genus *Allotrichoma* and its included subgenera and species groups, we also provide a cladistic analysis of genera within the tribe Hecamedini. The ingroup included seven exemplar congeners from within *Allotrichoma*. Outgroup sampling included exemplars of other genera within Hecamedini and from the putative sister group, Lipochaetini, and to root the analysis, we used an exemplar of the tribe Discocerinini. Analyses with successive weighting and implied weighting recovered a monophyletic *Allotrichoma* and indicated clades within the genus. Eight new species are described (type locality in parenthesis): *Allotrichoma bifurcatum* (Utah. Utah: Lake Shore (40°06.9'N, 111°41.8'W; 1370 m)), *Allotrichoma dynatum* (Oregon. Benton: Finley National Wildlife Refuge (44°24.6'N, 123°19.5'W)), *Allotrichoma occidentale* (Oregon. Lake: Lakeview (44 km E; Drake Creek; 42°11'N, 119°59.3'W)), *Allotrichoma robustum* (California. Kern: Kern River (35°16.1'N, 119°18.4'W)), *Allotrichoma sabroskyi* (New Mexico. Sandoval: La Cueva (Junction of Highways 126 and 4; 35°52'N, 106°38.4'W; 2342 m)), *Allotrichoma wallowa* (Oregon Baker: Goose Creek (35 km E Baker City; 44°49.2'N, 117°27.79'W; 825 m)), *Allotrichoma baliops* (Florida. Monroe: Key West (Willie Ward Park; 24°32.9'N, 81°47.9'W)), and *Allotrichoma insulare* (Dominica. Cabrits Swamp (15°35'N, 61°29'W)). Within *Allotrichoma*, we recognize three subgenera of which one, *Neotrichoma* (type species: *Allotrichoma atrilabre*), is newly described. All known species from the New World are described with an emphasis on structures of the male terminalia, which are fully illustrated. Detailed locality data and distribution maps for the New World species are provided. A lectotype is designated for *Discocerina simplex* Loew and a neotype is designated for *Allotrichoma bezzii* Becker. *Allotrichoma filiforme* Becker, *Allotrichoma trispinum* Becker, and *Allotrichoma dahli* Beschovski are reported as new synonyms of *Allotrichoma simplex* (Loew) and *Allotrichoma yosemite* Cresson is a new synonym of *Allotrichoma atrilabre* Cresson. We also clarify the status of previously described species, including those with Holarctic distributions. For perspective and to facilitate genus-group and species-group recognition, the tribe Hecamedini is diagnosed and a key to included genera is provided.

## Introduction

Only a few genera of shore flies (Diptera: Ephydridae) have greater species diversity in the Old World than in the New and also have the extant diversity in the New World primarily found in temperate areas of the Nearctic Region, sometimes exclusively so. Examples of these distributional and diversity patterns within shore flies are the genera *Ephydra* Fallén (Wirth 1971, 1975) and *Allotrichoma* Becker. The latter genus, *Allotrichoma*, is the subject of this revision, and why and how its distributional pattern developed and what the New World species-level diversity is have in part prompted this research. This revision also complements relatively recent and ongoing research on the Old World fauna of *Allotrichoma* (Krivosheina and Zatwarnicki 1997), since we also sought to know if any species found in the Palearctic Region actually occurs in the Nearctic Region, as has been reported (Cresson 1942, Wirth 1965, Mathis and Zatwarnicki 1995). Finally, discovering the phylogenetic relationships of taxa within and related to *Allotrichoma*, including relationships among taxa above the species level, continues to intrigue us and is also investigated here.

The discovery and correct identification of species and then accurately characterizing them are fundamental and preliminary steps to any research on the classification and distribution of shore flies. Problems associated with inaccurate species identification of *Allotrichoma* became apparent when the genus was first proposed. Becker (1896) first described *Allotrichoma* and designated *Hecamede lateralis* Loew as its type species. The type species, *Allotrichoma laterale*, was also reported to occur in North America (Cresson 1942, Wirth 1965), but these records were apparently based on misidentifications and have not been confirmed herein. In the same paper, however, and based on specimens collected in Italy, Becker also described *Allotrichoma bezzii*, a species that we have discovered in the Nearctic Region, and thus to have a Holarctic distribution. Moreover, the first species to be described from the Nearctic Region was *Allotrichoma simplex* (Loew 1861), which we have discovered to also have a Holarctic distribution and to have junior synonyms that are based on specimens collected in Europe. Thus, the clarification and in some cases the correct determination of what species are represented in the Nearctic Region is a necessary first and crucial step to any elaboration of zoogeographic and/or phylogenetic relationships.

In addition to Loew and Becker’s species, *Allotrichoma simplex* and *Allotrichoma bezzii*, Cresson (1926) described four species: *Allotrichoma lasiocercum*, *Allotrichoma lacteum*, *Allotrichoma atrilabre*, and *Allotrichoma yosemite*. We (Mathis and Zatwarnicki 2010) recently added *Allotrichoma deonieri*, which was described as part of a study on the fauna of the Delmarva States. No other species have been described from the Nearctic Region. These species, with the exception of *Allotrichoma deonieri*, were included in Cresson’s (1942) review of Nearctic shore flies, and some of these species have also been listed in more localized faunal papers, such as Johnson’s (1925) list of New England flies, and in papers dealing with natural history, such as Runyan and Deonier (1979). Faunistic and natural history papers are noted in the synonymy of each respective species. Wirth (1965, 1968) and Mathis and Zatwarnicki (1995) included all then known species in their respective catalogs.

Although most speciose genera of Nearctic shore flies have been treated comprehensively in the last 30 years (Mathis and Zatwarnicki 1995), an exception is *Allotrichoma* sensu stricto. Certainly, the small size of adults, which are usually less than 2.50 mm in body length, has detracted from their study, but Wirth (1965) identified a more generic concern in his annotation to this genus in his catalog (Wirth 1965) of Nearctic shore flies. Wirth noted that the species of *Allotrichoma* were poorly understood and that revision should include study of structures of the male terminalia to clarify the species and their distributions. Implied, although not stated, is that specimens are virtually indistinguishable externally. This study confirms Wirth’s observation, and following his suggestion and what we have observed in our own studies, we have relied wholly on structures of the male terminalia to make species-level identifications. Thus the key and species’ descriptions rely substantially on these structures.

In this revision, we more than double the number of species known from the New World with six of 12 species in the subgenus *Allotrichoma* being described herein, and in a newly described subgenus, *Neotrichoma*, two of the three species from the New World are also described. We also clarify the status of previously described species, including those with Holarctic distributions.

To provide phylogenetic perspective to this revision, we studied all genus-group taxa within the tribe Hecamedini and reassessed their cladistic relationships. Our phylogenetic study for taxa within the genus *Allotrichoma* was done at the species-group level, and we attempted to approach this globally by examining species from throughout the world and placing them into appropriate species groups. This global approach was done in an effort to adhere to the recommendations of Schmid (1979) and Schefter (2005), i.e., “Recognition of monophyletic groups of species as genera without reference to the world fauna might engender taxonomic inflation at the generic level and thereby erode the cognitive value of the genus.”

**Figure 1.**
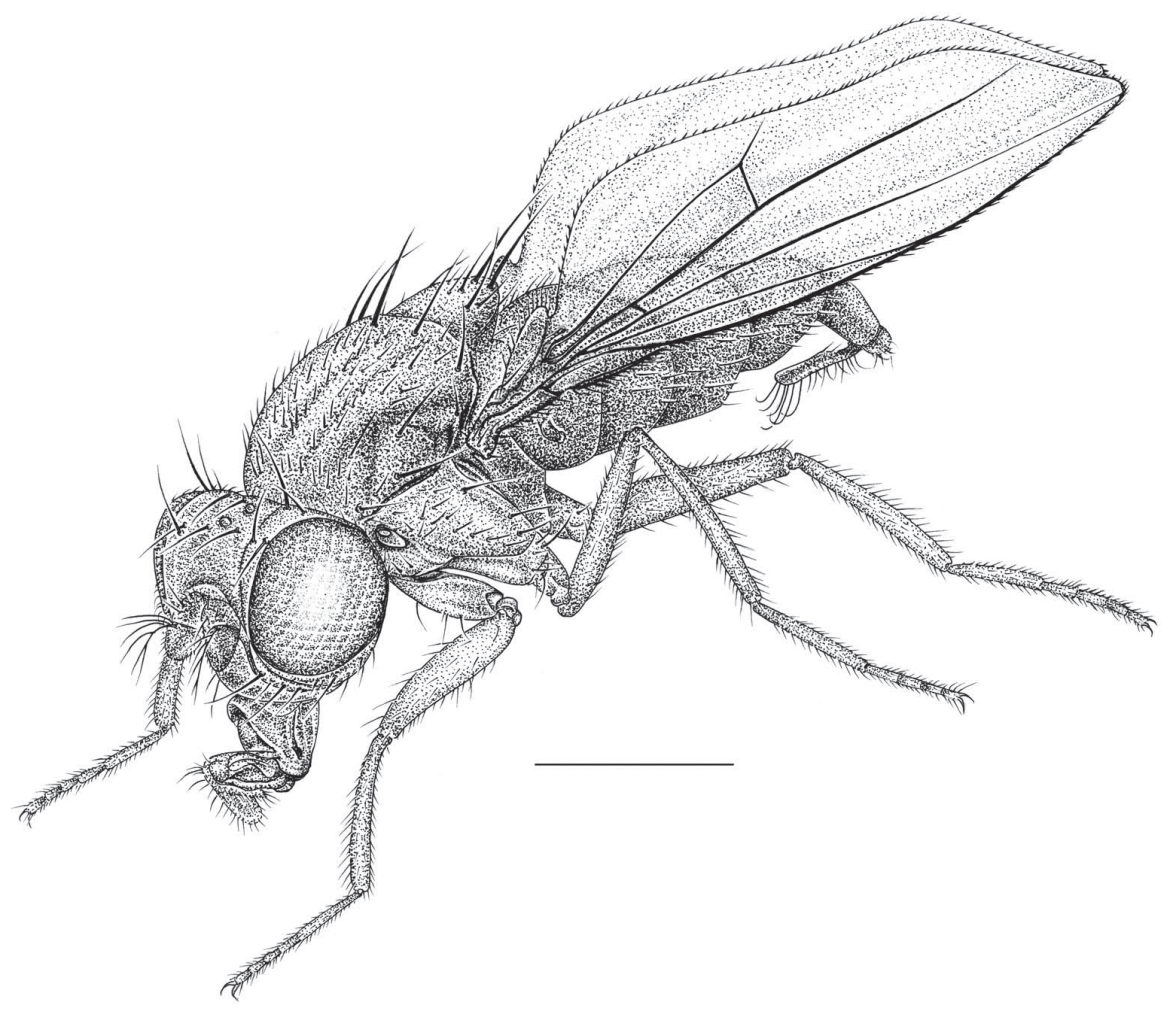
Habitus frontispiece of *Allotrichoma bezzii* Becker.

## Methods and materials

The descriptive terminology, with the exceptions noted in Mathis (1986b) and Mathis and Zatwarnicki (1990a) and below, follows that published in the *Manual of Nearctic Diptera* (McAlpine, 1981). Specimens of *Allotrichoma* are small and study and illustration of the male terminalia required use of a compound microscope. We have followed the terminology for most structures of the male terminalia that other workers in Ephydridae have used (see references in Mathis 1986b, and Mathis and Zatwarnicki 1990a, 1990b), such as surstylus. Zatwarnicki (1996) has suggested that the pre- and postsurstylus correspond with the pre- and postgonostylus and that the subepandrial plate is the same as the medandrium. The terminology for structures of the male terminalia is provided directly on Figs 3–7 and is not repeated for comparable illustrations of other species. Alternative spellings for some localities are cited in parenthesis, especially for locality names that were transliterated into English. Species’ descriptions are composite and not based solely on the holotypes, and paired structures are described in the singular except where the context makes this inappropriate. Two venational ratios used in the descriptions are based on the largest, smallest, and one other specimen and are defined below:

1. Costal vein ratio is the straight line distance between the apices of veins R_2+3_ and R_4+5_ divided by the distance between the apices of veins R_1_ and R_2+3_.

2. M vein ratio is the straight line distance along vein M between crossvein dm-cu and r-m divided by the distance apicad of crossvein dm-cu.

Distribution maps were made using ESRI ArcView® GIS 3.2. Longitude and latitude coordinates were obtained for the locality where each specimen was collected and entered into a Microsoft Excel® spreadsheet. If unavailable directly from specimen labels, longitude and latitude were estimated using gazetteers and maps to determine the geographical coordinates.

The phylogenetic analysis was performed with the assistance of Hennig86© and TNT©, computerized algorithms that produces cladograms by parsimony. Character data were polarized primarily using outgroup procedures. Although autapomorphies were not included in the cladistic analysis (they were made inactive), which would skew the consistency and retention indices, we listed them on the cladogram and included them as part of generic treatments and phylogenetic considerations to document the monophyly of the lineages, particularly at the generic level.

Because only males can now be accurately identified at the species level, we have limited paratype series of new species to that gender only. This practice became especially apparent when we discovered that species of *Allotrichoma* often occur together, with up to four or five species being found at the same locality (La Cueva, New Mexico; Goose Creek, Oregon; Deadman Canyon, Utah; Swasey Beach, Utah). Thus, not now being able to distinguish females and to make positive, species associations of males and females often precludes designating females as paratypes.

Although dissection of male abdomens is ordinarily needed to make species-level identifications, many specimens have these structures exposed, which facilitate their examination. When we pin adult males before they have dried, we often gently squeeze the abdomen slightly, usually extruding these structures, or we carefully tease these structures out with a pin.

Dissections of male terminalia were performed using the method of Clausen and Cook (1971) and Grimaldi (1987). Microforceps were used to remove abdomens, which were macerated in a potassium hydroxide solution. Cleared genitalia were rinsed in water and then transferred to glycerin for observation and illustration. Abdomens were placed in an attached plastic microvial filled with glycerin and attached to the pin supporting the remainder of the insect from which it was removed.

Although most specimens for this study, including the primary types, are in the National Museum of Natural History (USNM), numerous others were borrowed, particularly type specimens of the species previously described. To our colleagues and their institutions listed below who loaned specimens, we express our sincere thanks. Without their cooperation this study could not have been completed.

AMNHAmerican Museum of Natural History, New York, New York (Dr. David A. Grimaldi)

ANSPAcademy of Natural Sciences of Philadelphia, Pennsylvania (Drs. Jon K. Gelhaus, Donald F. Azuma, and Jason D. Weintraub)

BYUMonte L. Bean Museum, Brigham Young University, Provo, Utah (Dr. Shawn M. Clark)

CASCalifornia Academy of Sciences, San Francisco, California (Dr. Paul H. Arnaud, Jr.)

CNCCanadian National Collection, Ottawa, Canada (Dr. Jeffrey M. Cumming and Mr. Bruce Cooper)

HNHMHungarian Natural History Museum, Budapest, Hungary (Dr. László Papp)

KSUKansas State University, Manhattan, Kansas (Dr. Gregory Zolnerowich)

KUSnow Entomological Museum, University of Kansas, Lawrence, Kansas (Drs. Michael S. Engel and George W. Byers)

LACMLos Angeles County Museum, California (Drs. Brian V. Brown and Charles L. Hogue (deceased))

MCZMuseum of Comparative Zoology, Harvard University, Cambridge, Massachusetts (Dr. Philip D. Perkins)

MSNMMuseo di Storia Naturale di Milano, Milano, Italia (Dr. Maurizio Pavesi)

OSUOregon State University, Corvallis, Oregon (Drs. John D. Lattin, Christopher Marshall)

UARUniversity of Arizona, Tucson, Arizona (Drs. Floyd Werner, David R. Maddison, Carl Olson)

UCOUniversity of Colorado, Boulder, Colorado (Dr. M. Deane Bowers)

UCRUniversity of California at Riverside, Riverside, California (Drs. Saul Frommer and Douglas Yanega)

UMSPUniversity of Minnesota, St. Paul, Minnesota, United States (Dr. Philip J. Clausen)

USNMformer United States National Museum, collections in the National Museum of Natural History, Smithsonian Institution, Washington, D. C.

USUUtah State University, Logan, Utah (Dr. Wilford J. Hansen)

WSUMaurice T. James Collection, Department of Entomology, Washington State University, Pullman, Washington (Dr. Richard S. Zack)

ZILZoological Institute, Lund University, Lund, Sweden (Dr. Roy Danielsson)

ZMSZoological Museum, Bulgarian Academy of Sciences, Sofia, Bulgaria (Dr. Venelin L. Beschovski)

ZMHUZoologisches Museum, Humboldt Universität, Berlin, Germany (Dr. Joachim Ziegler)

## Systematics

### Tribe Hecamedini

Mathis

Hecamedini Mathis 1991: 2. Type genus: *Hecamede*Haliday 1837 [phylogeny]. Mathis and Zatwarnicki 1995: 149–160 [world catalog].

#### Diagnosis.

The tribe Hecamedini is distinguished from other tribes of Gymnomyzinae by the following combination of characters: *Head*: Arista with 3–5 dorsally branching rays, longer 2 or 3 rays subequal, inserted toward aristal base; compound eye bare of microsetulae or the latter very sparse. *Thorax*: Usually with a gray to silvery stripe on thorax from postpronotum through ventral portion of notopleuron; anterior supra-alar seta lacking; posterior notopleural seta inserted at distinctly elevated position, especially as compared to anterior seta; anepisternum usually two toned, dorsal portion concolorous with mesonotum, ventral portion gray; anepisternum with 2 subequal setae inserted along posterior margin. *Wing*: venation of wing generally pale colored; vein R_2+3_ elongate, section III much shorter than section II; apical section of vein M longer than section between crossveins r-m and dm-cu; alula wide, width subequal to that of costal cell. *Abdomen*: Male terminalia: Pregonite either lacking or fused indistinguishably with postgonite; subepandrial sclerite lacking; postgonite generally elongate and bearing few setulae, usually only 2 are conspicuous.

#### Phylogenetic considerations. 

Mathis (1991) placed *Allotrichoma* in the tribe Hecamedini Mathis (subfamily Gymnomyzinae), and in his proposed phylogeny for genera within Hecamedini, *Allotrichoma* and related genera comprised the most derived lineages within the tribe. In this study, we have re-examined the evidence, all morphological characters, in an attempt to discover more definitively the phylogenetic placement of *Allotrichoma* and related taxa, especially those taxa that have been treated as subgenera within *Allotrichoma*. The basic question is “What are the phylogenetic relationships (hypothetical) among taxa closely related to or within *Allotrichoma*?” As background, we first present a summary of the evidence and relationships at the tribal level (within Gymnomyzinae) and then proceed to the evidence and our analysis of it for taxa within Hecamedini.

The tribe Hecamedini, which is one of six tribes now placed in the subfamily Gymnomyzinae (Mathis and Zatwarnicki 1995), appears to be most closely related to the tribe Lipochaetini (Zatwarnicki 1992, Mathis and Zatwarnicki 2004). Hecamedini’s sister-group relationship with Lipochaetini is corroborated by two synapomorphies (Zatwarnicki’s (1992) characters 59 and 60): 1. Pre- and postgonites fused and/or reduced; 2. Posterior notopleural seta inserted dorsad of level of anterior seta (thoracic character 7(28) below).

Hecamedini are distinguished from Lipochaetini, and the tribe’s monophyly is confirmed by the following characters (synapomorphies are noted by an *): *1. Arista bearing 3–5 dorsally branching rays, longer 2–3 rays subequal, inserted toward aristal base (character 7(9) below); *2. Compound eye bare of microsetulae or the latter very sparse (character 8(10) below); *3. Usually with a gray to silvery stripe on thorax from postpronotum through ventral portion of notopleuron (character 6(27) below); *4. Anterior presutural supra-alar seta lacking (character 3(24) below); *5. Posterior notopleural seta inserted at distinctly elevated position, especially as compared to anterior seta (character 8(29) below); 6. Venation of wing generally pale colored; 7. Gonite present, triangular to narrowly triangular (character 13(49) below), and bearing few setulae, usually only 2 are conspicuous (Discocerinini, Hecamedini; lacking in Lipochaetini) (character 14 (50) below).

**Figure 2.**
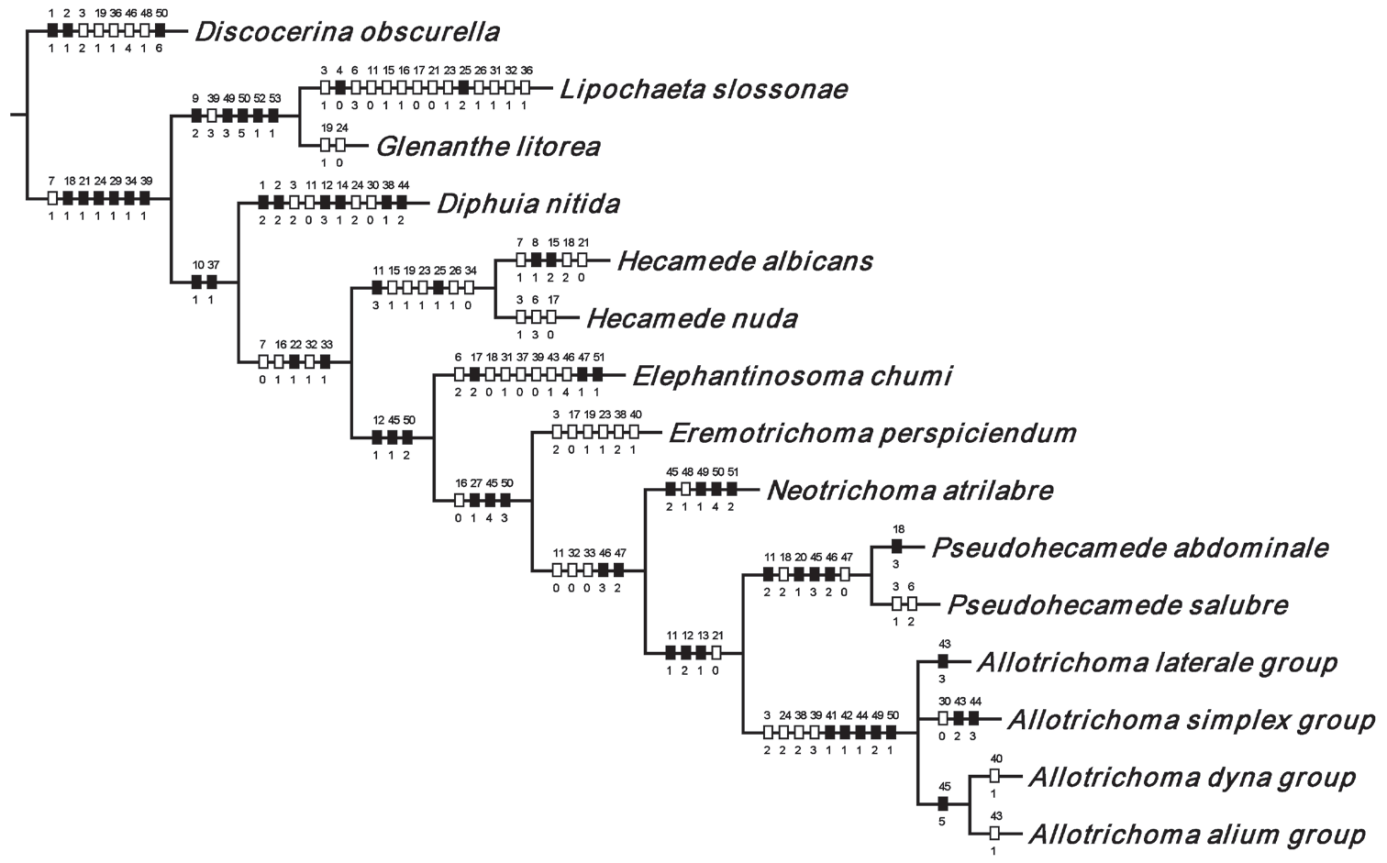
Hypothetical phylogeny of taxa within the tribe Hecamedini. Numbers in parenthesis correspond with those in the text. The most parsimonious tree required 135 steps, and after implied weighting has a consistency index of 0.89, and a retention index of 0.90.

With the phylogenetic background for further study of the tribe Hecamedini within the subfamily Gymnomyzinae established and the monophyly of Hecamedini documented, we now proceed with the cladistic analysis and resultant relationships among the included genera, but with a few explanatory remarks first. In the presentation on genus-level relationships that follows, the characters used in the analysis are listed first. Each character is immediately followed by a discussion to explain its states and to provide perspective and any qualifying comments about that character. After presentation of the information on character evidence, an hypothesis of the cladistic relationships is presented and briefly discussed. A detailed, species-level phylogeny is beyond the scope of this paper, especially as *Allotrichoma* *sensu stricto* is essentially found worldwide except for the Neotropical Region, and herein we focus primarily on the New World fauna. Our intent here is to present evidence and an analysis of that evidence in an attempt to determine intermediate clusters of taxa, such as subgenera and species groups within *Allotrichoma*. We have allocated all taxa within *Allotrichoma* to one of three subgenera (*Allotrichoma*, *Neotrichoma*, *Pseudohecamede*), and within the subgenus *Allotrichoma*, four species groups (*alium*, *dyna*, *laterale*, and *simplex* species groups). The New World species of the subgenus *Allotrichoma* are in two of the species groups: *laterale* (*Allotrichoma bezzii*, *Allotrichoma deonieri*, *Allotrichoma dynatum*, *Allotrichoma lacteum*, *Allotrichoma lasiocercum*, *Allotrichoma occidentale*, *Allotrichoma robustum*, *Allotrichoma sabroskyi*, and *Allotrichoma schumanni*) and *simplex* (*Allotrichoma bifurcatum*, *Allotrichoma simplex*, and *Allotrichoma wallowa*). The cladogram (Fig. 2) is the primary mode to convey relationships, and the discussion is to supplement the cladogram and is intended only to complement the latter. In the discussion of character data, a “0” indicates the state of the outgroup; a “1” or “2” indicates the derived states. Multistate characters (1, 2, 3, 4, 6, 9, 11, 12, 15, 17, 18, 25, 31, 33, 34, 40, 43, 44, 45, 46, 47, 49, 50, 51), which comprise 46 percent of the total number, were treated as nonadditive (-), and characters. The numbers used for characters in the presentation are the same as those on the cladogram, and the sequence is the same as noted in the character matrix (Table 1). For polarization of character states within Hecamedini, we used the tribes Lipochaetini (*Glenanthe* Haliday and *Lipochaeta*) and Discocerinini (*Discocerina obscurella*) as the successive outgroups in this phylogenetic analysis. Runyan and Deonier (1979) cited six morphological characters from immature stages in their preliminary phylogenetic analysis. While these may have phylogenetic significance, we know so little about these characters for the taxa being treated that we are hesitant to use them. The majority of taxa would be represented by a question mark in the matrix, as we known virtually nothing about their distribution among related taxa. Hopefully this revision will promote further research on immature stages, including field work, and allow us to incorporate these kinds of characters into an analysis.

**Table 1.** Matrix of characters and taxa used in the cladistic analysis of *Allotrichoma* (numbers for characters correspond with those used in the text).

**TAXA**	**CHARACTERS**			
	G	Head	Thorax	Abdomen
character number	00	0000000111111111122	222222223333333	33344444444445555
	12	3456789012345678901	234567890123456	78901234567890123
*Discocerina obscurella*	11	2111001040000010100	000000001000001	00000000040106000
*Lipochaeta slossonae*	00	1013102000001101000	011210011110101	00300000000035011
*Glenanthe litorale*	00	0111102040000011101	000000011000100	00300000000035011
*Elephantinosoma chnumi*	00	0112001141000120001	101000011111100	00000010141002100
*Diphuia nitida*	22	2111101103010011001	002000010000100	11100002000000000
*Allotrichoma (Allotrichoma) dyna*	00	2111001112100011000	102001011000100	12311101532021000
*Allotrichoma (Allotrichoma) alium* *group*	00	2111001112100011000	102001011000100	12301111532021000
*Allotrichoma (Allotrichoma) laterale* *group*	00	2111001112100011000	102001011000100	12301131432021000
*Allotrichoma (Allotrichoma) simplex* *group*	00	2111001112100011000	102001010000100	12301123432021000
*Allotrichoma (Neotrichoma) atrilabre*	00	0111001101000011001	101001011000100	10100000232114200
*Allotrichoma (Pseudohecamede) abdominale*	00	0111001122100013010	101001011000100	10100000320003000
*Allotrichoma (Pseudohecamede) salubre*	00	1112001122100012010	101001011000100	10100000320003000
*Eremotrichoma*	00	2111001141000001101	111001011011100	12110000400003000
*Hecamede albicans*	00	0111111130002112100	111110011011000	10100000000000000
*Hecamede nuda*	00	1113001130001101101	111110011011000	10100000000000000

#### Characters Used in the Analysis. (running count in parenthesis)

**GENERAL**

1 (1) Vestiture in general (nonadditive): (0) partially to mostly microtomentose (*Glenanthe*, *Lipochaeta*, *Hecamede albicans*, *Hecamede nuda*, *Allotrichoma (Pseudohecamede) abdominale*, *Allotrichoma (Pseudohecamede) salubre*, *Elephantinosoma*, *Eremotrichoma*, *Neotrichoma*, *Allotrichoma* *sensu stricto*); (1) mostly bare, shiny (*Discocerina*); (2) *Diphuia* (secondarily bare).

2 (2) Coloration in general (nonadditive): (0) gray to tan (*Glenanthe*, *Lipochaeta*, *Hecamede albicans*, *Hecamede nuda*, *Elephantinosoma*, *Eremotrichoma*, *Allotrichoma* *sensu lato* (including *Neotrichoma*, *Allotrichoma (Pseudohecamede) abdominale*, *Allotrichoma (Pseudohecamede) salubre*)); (1) mostly black (*Discocerina*); (2) *Diphuia* (secondarily mostly black).

**HEAD**

1 (3) Size of pseudopostocellar setae (nonadditive): (0) normally developed (length about equal to distance between a posterior ocellus and anterior ocellus) (*Glenanthe*, *Elephantinosoma*, *Allotrichoma (Pseudohecamede) abdominale*, *Hecamede albicans*, *Neotrichoma*); (1) greatly reduced (length subequal to diameter of posterior ocellus) or absent (*Lipochaeta*, *Hecamede nuda*, *Allotrichoma (Pseudohecamede) salubre*); (2) well developed, proclinate (*Discocerina*, *Diphuia*, *Eremotrichoma*, *Allotrichoma* *sensu stricto*).

2 (4) Position of ocellar setae (nonadditive): (0) inserted posterior to transverse alignment of anterior ocellus (most Gymnomyzinae (Gastropini, Gymnomyzini, Lipochaetini, Ochtherini)); (1) inserted anterior to transverse alignment of anterior ocellus (*Glenanthe*, Hecamedini, *Discocerina*); (2) not distinguished from setulae, greatly reduced or lacking (*Lipochaeta*).

3 (5) Alignment of fronto-orbital setae: (0) aligned posterior to ocellar setae (most Ephydridae except for Hecamedini and Atissini); (1) aligned transversely with ocellar setae (Lipochaetini, Hecamedini, *Discocerina*, also in Atissini of the subfamily Hydrelliinae).

4 (6) Relative size of fronto-orbital setae (nonadditive): (0) reclinate seta larger than proclinate seta; (1) reclinate and proclinate subequal (*Discocerina*, *Glenanthe*, *Hecamede albicans*, *Diphuia*, *Eremotrichoma*, *Allotrichoma* *sensu stricto*, *Allotrichoma (Pseudohecamede) abdominale*, *Neotrichoma*); (2) only reclinate seta well developed (*Elephantinosoma*, *Allotrichoma (Pseudohecamede) salubre*); (3) not distinguished from setulae, greatly reduced or lacking (*Lipochaeta*, *Hecamede nuda*).

5 (7) Setulae on frons: (0) lacking setulae (*Discocerina*, *Diphuia*, *Elephantinosoma*, *Eremotrichoma*, *Allotrichoma* *sensu lato*, *Hecamede nuda*); (1) bearing numerous setulae (*Glenanthe*, *Lipochaeta*, *Hecamede albicans*).

6 (8) Interfrontal setae (an interfrontal seta in addition to ocellar setae, which are usually inserted more anteriorly, usually just anterior of anterior ocellus in Hecamedini): (0) lacking (*Discocerina*, *Glenanthe*, *Lipochaeta*, *Diphuia*, *Elephantinosoma*, *Eremotrichoma*, *Allotrichoma sensu lato*, *Hecamede nuda*); (1) present (*Hecamede albicans*).

7 (9) Aristal setae (nonadditive): (0) bearing 5–10 dorsally branching rays more or less evenly along arista (*Discocerina*); (1) bearing 3–5 dorsally branching rays, longer 2–3 rays subequal, inserted toward aristal base (a synapomorphy for the tribes Gymnomyzini, Hecamedini); (2) a brush, with short setulae both dorsally and ventrally (Lipochaetini).

8 (10) Microsetulae on compound eye: (0) bearing microsetulae (*Discocerina*, Lipochaetini, and also Hydrelliinae, probably through convergence); (1) lacking microsetulae or these very sparse (Gymnomyzinae except for Lipochaetini).

9 (11) Facial conformation (nonadditive): (0) distinctly but shallowly convex (Lipochaeta); (1) shallowly conically produced (*Diphuia*, *Allotrichoma* *sensu stricto*, *Neotrichoma*); (2) conically protrudent and carinate dorsally (*Allotrichoma (Pseudohecamede) abdominale*, *Allotrichoma (Pseudohecamede) salubre*); (3) distinctly conically produced, “tuberculate” (*Hecamede albicans*, *Hecamede nuda*); (4) shallowly carinate dorsally (*Discocerina*, *Glenanthe*, *Elephantinosoma*, *Eremotrichoma*).

10 (12) Facial coloration (nonadditive): (0) uniformly colored (*Discocerina*, *Glenanthe*, *Lipochaeta*, *Hecamede albicans*, *Hecamede nuda*); (1) darker between antennae, lighter, usually gray to whitish gray ventrally (*Elephantinosoma*, *Eremotrichoma*, *Neotrichoma*); (2) females two toned, dark dorsally (brown to golden brown), light colored ventrally; males generally uniformly dark colored (*Allotrichoma* *sensu lato*); (3) patterned, black with vertical, silvery white microtomentose stripes and dots (*Diphuia*).

11 (13) Facial coloration: (0) not sexually dimorphic (*Discocerina*, *Glenanthe*, *Lipochaeta*, *Hecamede albicans*, *Hecamede nuda*, *Elephantinosoma*, *Eremotrichoma*, *Neotrichoma*, *Diphuia*); (1) sexually dimorphic females two toned, dark dorsally (brown to golden brown), light colored ventrally; males are generally uniformly dark colored (*Allotrichoma* *sensu stricto*, *Allotrichoma (Pseudohecamede) abdominale*, *Allotrichoma (Pseudohecamede) salubre*).

12 (14) Facial vestiture: (0) generally microtomentose (*Discocerina*, *Glenanthe*, *Lipochaeta*, *Elephantinosoma*, *Eremotrichoma*, *Hecamede albicans*, *Hecamede nuda*, *Allotrichoma* *sensu lato*); (1) generally bare except for silvery white microtomentose stripes and dots (*Diphuia*).

13 (15) Genal seta (nonadditive): (0) present, distinct from other setulae if the latter are present (*Discocerina*, *Glenanthe*, *Elephantinosoma*, *Diphuia*, *Eremotrichoma*, *Allotrichoma* *sensu lato*); (1) absent (*Lipochaeta*, *Hecamede nuda*); (2) several setulae but not distinct seta (*Hecamede albicans*).

14 (16) Genal height (eye-to-cheek ratio): (0) 0.27 and less (*Discocerina*, *Glenanthe*, *Diphuia*, *Eremotrichoma*, *Allotrichoma* *sensu lato*); (1) 0.34 and greater (*Lipochaeta*, *Elephantinosoma*, *Hecamede*).

15 (17) Width of oral margin (nonadditive): (0) moderately wide (*Lipochaeta*, *Eremotrichoma*, *Hecamede nuda*); (1) narrow (*Discocerina*, *Glenanthe*, *Diphuia*, *Allotrichoma* *sensu lato*, *Hecamede albicans*); (2) wide (*Elephantinosoma*).

16 (18) Shape of ventral margin of face (nonadditive): (0) flat or nearly so (*Elephantinosoma*); (1) shallowly emarginate (*Discocerina*, *Glenanthe*, *Lipochaeta*, *Eremotrichoma*, *Diphuia*, *Allotrichoma* *sensu stricto*, *Hecamede nuda*, *Neotrichoma*); (2) distinctly emarginate anteriorly, concave with clypeus exposed in emargination. (*Allotrichoma (Pseudohecamede) abdominale*, *Allotrichoma (Pseudohecamede) salubre*, *Hecamede albicans*)

17 (19) Palpal color: (0) dark colored, brown to blackish brown (*Lipochaeta*, *Elephantinosoma*, *Diphuia*, *Allotrichoma* *sensu lato*); (1) pale, yellowish to whitish (*Discocerina*, *Glenanthe*, *Eremotrichoma*, *Hecamede albicans*, *Hecamede nuda*).

18 (20) Shape of mouthparts: (0) normally developed, mediproboscis not especially elongate (*Discocerina*, *Glenanthe*, *Lipochaeta*, *Elephantinosoma*, *Hecamede albicans*, *Hecamede nuda*, *Eremotrichoma*, *Diphuia*, *Allotrichoma* *sensu stricto*, *Neotrichoma*); (1) geniculate, with elongate mediproboscis (*Allotrichoma (Pseudohecamede) abdominale*, *Allotrichoma (Pseudohecamede) salubre*).

19 (21) Clypeal vestiture: (0) microtomentose, usually gray (*Discocerina*, *Lipochaeta*, *Hecamede albicans*, *Allotrichoma (P. ) abdominale*, *Allotrichoma (Pseudohecamede) salubre*, *Allotrichoma* *sensu stricto*); (1) thinly microtomentose or bare, appearing black (*Glenanthe*, *Elephantinosoma*, *Eremotrichoma*, *Hecamede nuda*, *Diphuia*, *Neotrichoma*).

**THORAX**

1 (22) Number of rows of acrostichal setae, especially on posterior half: (0) 3–4 rows (*Discocerina*, *Glenanthe*, *Lipochaeta* (greatly reduced and difficult to discern), *Hecamede nuda*, *Diphuia*); (1) 2 rows (*Hecamede albicans*, *Eremotrichoma*, *Elephantinosoma*, *Allotrichoma* *sensu lato*). There is a tendency for less rows of setulae on the anterior half of the scutum. We numbered rows on one side and from the posterior half of the scutum.

2 (23) Prescutellar acrostichal setae: (0) present (*Discocerina*, *Glenanthe*, *Diphuia*, *Elephantinosoma*, *Allotrichoma* *sensu lato*); (1) greatly reduced or absent (*Lipochaeta*, *Hecamede albicans*, *Hecamede nuda*, *Eremotrichoma*).

3 (24) Presutural supra-alar seta (additive): (0) present (*Discocerina*, *Glenanthe*); (1) greatly reduced or absent (*Lipochaeta*, *Elephantinosoma*, *Hecamede albicans*, *Hecamede nuda*, *Allotrichoma (Pseudohecamede) abdominale*, *Eremotrichoma*, *Elephantinosoma*, *Neotrichoma*, *Allotrichoma (Pseudohecamede) salubre*); (2) secondarily reversed (*Diphuia*, *Allotrichoma* *sensu stricto*). In *Hecamede albicans*, this seta is only slightly larger than surrounding setae.

4 (25) Number of lateral scutellar setae (nonadditive): (0) 2 pairs (*Discocerina*, *Glenanthe*, *Elephantinosoma*, *Diphuia*, *Eremotrichoma*, *Elephantinosoma*, *Allotrichoma* *sensu lato*); (1) 3 pairs (*Hecamede albicans*, *Hecamede nuda*); (2) not distinguished from setulae, greatly reduced or lacking (*Lipochaeta*).

5 (26) Number of setulae on scutellar disc: (0) 0 to a few scattered setulae, less than 15 (*Discocerina*, *Glenanthe*, *Elephantinosoma*, *Hecamede nuda*, *Diphuia*, *Eremotrichoma*, *Elephantinosoma*, *Allotrichoma* *sensu lato*); (1) numerous setulae, more than 20, evenly scattered (*Lipochaeta*, *Hecamede albicans*).

6 (27) Pleural stripes: (0) lacking stripes (*Discocerina*, *Glenanthe*, *Lipochaeta*, *Elephantinosoma*, *Diphuia*, *Hecamede albicans*, *Hecamede nuda*, *Elephantinosoma*); (1) with a stripe (*Eremotrichoma*, *Allotrichoma* *sensu lato*).

7 (28) Anterior notopleural seta: (0) present, subequal to posterior seta (*Discocerina*, *Glenanthe*, *Lipochaeta*, *Hecamede albicans*, *Diphuia*, *Eremotrichoma*, *Elephantinosoma*, *Allotrichoma* *sensu lato*); (1) greatly reduced or absent (*Hecamede nuda*).

8 (29) Position of posterior notopleural seta: (0) inserted at same level as anterior seta (*Discocerina*); (1) inserted a more dorsal position from level of anterior seta (Hecamedini, Lipochaetini, and also many Atissini in the subfamily Hydrelliinae, probably by convergence).

9 (30) Anepisternal setulae: (0) reduced (less than 10) and/or lacking (*Hecamede nuda*, *Diphuia*, *Eremotrichoma*, *Elephantinosoma*, *Neotrichoma*, *Allotrichoma* *sensu lato*); (1) numerous present, especially on dorsal 1/3 (*Discocerina*, *Glenanthe*, *Lipochaeta*, *Hecamede albicans*). The usual condition is for none to a few setulae in addition to the two setae along the posterior margin. If present, the setulae are usually toward the center on the dorsal half.

10 (31) Katepisternal seta (nonadditive): (0) present, well developed (*Discocerina*, *Glenanthe*, *Lipochaeta*, *Hecamede albicans*, *Diphuia*, *Eremotrichoma*, *Allotrichoma* *sensu lato*); (1) absent (*Elephantinosoma*); (2) absent (*Hecamede nuda*).

11 (32) Color of wing membrane: (0) hyaline (*Discocerina*, *Glenanthe*, *Diphuia*, *Neotrichoma*, *Allotrichoma* *sensu stricto*); (1) lacteous (*Lipochaeta*, *Allotrichoma (Pseudohecamede) abdominale*, *Allotrichoma (Pseudohecamede) salubre*, *Hecamede albicans*, *Hecamede nuda*, *Eremotrichoma*, *Elephantinosoma*).

12 (33) Tibial coloration (nonadditive): (0) tibiae mostly to entirely dark, similar to femora (*Discocerina*, *Glenanthe*, *Lipochaeta*, *Diphuia*, *Allotrichoma* *sensu lato*); (1) mostly yellow, similar to basal tarsomeres, at most with thin investment of mostly white microtomentum (*Hecamede albicans*, *Eremotrichoma*, *Elephantinosoma*); (2) foretibia especially mostly grayish, only apices of foretibia mostly yellow (*Hecamede nuda*).

13 (34) Adornment of posteroventral surface of forefemur (nonadditive): (0) bearing 5–7 setae on apical 1/2–2/3 (*Discocerina*, *Hecamede albicans*); (1) setae lacking or with just 1–3 along this surface (*Glenanthe*, *Lipochaeta*, *Diphuia*, *Eremotrichoma*, *Elephantinosoma*, *Allotrichoma* *sensu lato*); (2) setae along this surface greatly enlarged (*Hecamede nuda*).

14 (35) Tarsomere 1 of foreleg: (0) lacking black setae at base of posteroventral surface (*Discocerina*, *Glenanthe*, *Lipochaeta*, *Hecamede albicans*, *Diphuia*, *Eremotrichoma*, *Elephantinosoma*, *Allotrichoma* *sensu lato*); (1) bearing 2 or 3 black setae inserted toward base of posteroventral surface (*Hecamede nuda*).

15 (36) Setae at base of tarsomere 1 of hindleg: (0) mostly yellow (*Glenanthe*, *Hecamede albicans*, *Diphuia*, *Eremotrichoma*, *Elephantinosoma*, *Allotrichoma* *sensu lato*); (1) 2–4 black setae inserted near base on anteroventral surface (*Discocerina*, *Lipochaeta*, *Hecamede nuda*).

**ABDOMEN**

1 (37) Number of abdominal tergites normally visible in ♂: (0) 5 abdominal tergites usually exposed and visible, none wholly retracted into preceding tergite (*Discocerina*, *Glenanthe*, *Lipochaeta*, *Elephantinosoma*); (1) only 4 abdominal tergites generally exposed and visible, tergite 5 mostly to wholly retracted within tergite 4 (*Hecamede*, *Eremotrichoma*, *Allotrichoma* *sensu lato*, *Diphuia*).

2 (38) Relationship of tergite 5 and sternite 5 (additive): (0) tergite separate from ventral sternite (*Discocerina*, *Glenanthe*, *Lipochaeta*, *Hecamede*, *Elephantinosoma*, *Allotrichoma (Pseudohecamede) abdominale*, *Allotrichoma (Pseudohecamede) salubre*, *Neotrichoma*); (1) tergite 5 moderately narrow, forming a complete ring, especially anteriorly, with sternite (*Diphuia*); (2) tergite relatively narrow, forming a tube-like structure, sternite 5 reduced (*Eremotrichoma*, *Allotrichoma* *sensu stricto* (= *alium*, *laterale*, and *simplex* groups)).

3 (39) Length of tergite 5 of ♂ (nonadditive): (0) tergite relatively short (length usually less than tergite 4), comparatively wide, trapezoidal and/or triangular (*Discocerina*, *Elephantinosoma*); (1) tergite 5 comparatively short, band-like (*Hecamede*, *Diphuia*, *Eremotrichoma*, *Neotrichoma*, *Allotrichoma (Pseudohecamede) abdominale*, *Allotrichoma (Pseudohecamede) salubre*); (2) tergite moderately long (*Glenanthe*, *Lipochaeta*); (3) tergite 5 comparatively long (*Allotrichoma* *sensu stricto*).

4 (40) Presence or absence of relatively large, anterior, digitiform, paired apodemes of tergite 5 of ♂: (0) absent (*Discocerina*, *Glenanthe*, *Lipochaeta*, *Elephantinosoma*, *Diphuia*, *Hecamede*, *Allotrichoma* *sensu lato*; (1) present (*Allotrichoma dyna*, *Eremotrichoma* sp. n. from Namibia (*incertae sedis*)).

5 (41) Condition of ventral margin of tergite 5 of ♂: (0) simple, unadorned (*Discocerina*, *Glenanthe*, *Lipochaeta*, *Elephantinosoma*, *Diphuia*, *Hecamede*, *Eremotrichoma*, *Neotrichoma*, *Allotrichoma (Pseudohecamede) abdominale*, *Allotrichoma (Pseudohecamede) salubre*): (1) with a ventral tergal process (these structures are paired, i.e., one on each side, and are apparently tergal in origin; *Allotrichoma* *sensu stricto*).

6 (42) Condition of sternite 5 of ♂: (0) simple, an unadorned, flat sclerite, lacking a medial process (*Discocerina*, *Glenanthe*, *Lipochaeta*, *Elephantinosoma*, *Diphuia*, *Hecamede*, *Eremotrichoma*, *Neotrichoma*, *Allotrichoma (Pseudohecamede) abdominale*, *Allotrichoma (Pseudohecamede) salubre*); (1) bearing a medial, unpaired structure with apical setulae (an autapomorphy of the *Allotrichoma* *sensu stricto*).

7 (43) Shape of cerci (nonadditive): (0) length normal, not longer than epandrium, oval (*Discocerina*, *Glenanthe*, *Lipochaeta*, *Hecamede*, *Diphuia*, *Allotrichoma (Pseudohecamede) abdominale*, *Allotrichoma (Pseudohecamede) salubre*, *Eremotrichoma*, *Neotrichoma*, *Allotrichoma dyna*); (1) length normal, not longer than epandrium, extensively fused laterally and ventrally with epandrium (*Elephantinosoma*, *Allotrichoma alium* group); (2) cerci greatly elongate, curved laterally, thinly developed (an autapomorphy of the *Allotrichoma simplex* group of *Allotrichoma* *sensu stricto*); (3) cerci greatly elongate, robustly developed, especially apically (an autapomorphy of the *Allotrichoma laterale* group of *Allotrichoma* *sensu stricto*).

8 (44) Vestiture of cerci (nonadditive): (0) not bearing elongate setae at ventral margin (*Discocerina*, *Glenanthe*, *Lipochaeta*, *Elephantinosoma*, *Hecamede*, *Eremotrichoma*, *Allotrichoma (Pseudohecamede) abdominale*, *Allotrichoma (Pseudohecamede) salubre*, *Neotrichoma*); (1) ventral margin bearing conspicuously longer setae (*Allotrichoma alium* and *Allotrichoma laterale* groups); (2) ventral margin bearing 2 elongate setae (*Diphuia*); (3) 2–4 short setae at ventral apex of cercus (*Allotrichoma simplex* group).

9 (45) Condition of surstylus (nonadditive): (0) separate from epandrium (*Discocerina*, *Glenanthe*, *Lipochaeta*, *Diphuia*, *Hecamede*); (1) lost or fused indistinguishably with epandrium (*Elephantinosoma*); (2) secondarily separate from epandrium (*Neotrichoma* sp. n. from Namibia (*incertae sedis*)); (3) fused to ventral margin of epandrium+cercus complex (*Allotrichoma (Pseudohecamede) abdominale*, *Allotrichoma (Pseudohecamede) salubre*); (4) fused to ventral margin of epandrium (*Eremotrichoma*, *Allotrichoma (laterale* and *simplex* groups); (5) secondarily fused indistinguishably with epandrium (*Allotrichoma alium* group).

10 (46) Size of surstylus (nonadditive): (0) large, distinct (*Glenanthe*, *Lipochaeta*, *Hecamede*, *Diphuia*, *Eremotrichoma*); (1) greatly reduced, rodlike (*Neotrichoma* sp. n. from Nimibia (*incertae sedis*)); (2) greatly reduced, lobelike (*Allotrichoma (Pseudohecamede) abdominale *, *Allotrichoma (Pseudohecamede) salubre*); (3) moderately reduced, usually narrow (*Allotrichoma* *sensu stricto*); (4) size not evident (*Discocerina*, *Elephantinosoma*).

11 (47) Shape of Phallapodeme (nonadditive): (0) typical, roughly quadrate to broadly triangular (*Discocerina*, *Glenanthe*, *Lipochaeta*, *Hecamede*, *Allotrichoma (Pseudohecamede) abdominale*, *Allotrichoma (Pseudohecamede) salubre*, *Diphuia*); (1) narrowly triangular (*Elephantinosoma*); (2) triangular with concave sides, producing narrow, digitiform-like extensions (*Allotrichoma* *sensu lato* without *Pseudohecamede*).

12 (48) Relationship of phallapodeme to base of aedeagus: (0) separate but approximate (*Glenanthe*, *Lipochaeta*, *Elephantinosoma*, *Hecamede*, *Diphuia*, *Allotrichoma* *sensu lato* without *Neotrichoma*); (1) phallapodeme fused to base of aedeagus (*Discocerina*, *Neotrichoma*).

13 (49) Shape of gonite (nonadditive): (0) broadly to moderately triangular (*Discocerina*, *Elephantinosoma*, *Hecamede*, *Diphuia*, *Eremotrichoma*, *Allotrichoma (Pseudohecamede) abdominale*, *Allotrichoma (Pseudohecamede) salubre*); (1) with an anterobasal process (*Neotrichoma*); (2) narrowly rectangular to almost tubular (*Allotrichoma* *sensu stricto*); (3) lacking a gonite (*Glenanthe*, *Lipochaeta*).

14 (50) Apical gonal setulae (nonadditive): (0) several setulae, especially along dorsal margin (*Hecamede*, *Diphuia*); (1) 2 setulae, usually 1 apical, the other apicoventral (*Allotrichoma* *sensu stricto*); (2) 1 subapical setulae (*Elephantinosoma*); (3) 3 setulae, 2 dorsal and 1 subapical (*Eremotrichoma*, *Allotrichoma (Pseudohecamede) abdominale*, *Allotrichoma (Pseudohecamede) salubre*); (4) 3 setulae (2 apicodorsally, 1 subapical) or 2 setulae (both apical) (*Neotrichoma*); (5) gonite and setulae lacking (*Glenanthe*, *Lipochaeta*); (6) 1 apical setula (*Discocerina*).

15 (51) Shape of aedeagus (nonadditive): (0) tubular (*Glenanthe*, *Lipochaeta*, *Hecamede*, *Diphuia*, *Eremotrichoma*, *Allotrichoma* *sensu stricto*, *Allotrichoma (Pseudohecamede) abdominale*, *Allotrichoma (Pseudohecamede) salubre*); (1) narrowly elongate (*Elephantinosoma*); (2) conspicuously arched (*Neotrichoma*).

16 (52) Apical aedeagal flap: (0) lacking, simple (Hecamedini); (1) present (Lipochaetini).

17 (53) Ejaculatory apodeme: (0) lacking (Hecamedini); (1) present (Lipochaetini).

#### Analysis and results.

Using the implicit enumeration (ie*) option of Hennig86 and TNT, which is an exhaustive search, 29 most parsimonious trees were generated from the analysis of the 53 characters. The cladograms have a length of 135 steps and consistency and retention indices of 0.64 and 0.65 respectively. The matrix was then subjected iteratively to successive weighting (xs w, ie*, cc) to determine a character’s contribution or weight (Carpenter 1988, Dietrich and McKamey 1995). We also used the implicit weighting option in TNT. The successive weighing stabilized at 559 steps, and with stabilization, the consistency and retention indices increased to 0.89 and 0.90, respectively. Both successive and implicit weighting schemes resulted in the same two cladograms. One of these cladograms is identical to one of the 29 original trees and is our cladogram of choice (Fig. 2), being one of the most parsimonious cladograms. The two cladograms only differ in the terminal relationships of the *simplex* species group, i.e., whether it forms a tritomy or whether the *simplex* group is the sister group to the other species groups in the subgenus *Allotrichoma*. The analysis of characters for this cladogram is given in Table 2 and the weights of the various characters are given in Table 3.

In summary and as indicated on the cladogram (Fig. 2), the tribe Hecamedini is a monophyletic lineage (unambiguous synapomorphies 10 and 37) that is closely related to Lipochaetini (the outgroup), and these two tribes together (Hecamedini and Lipochaetini) are particularly well supported (synapomorphies 7, 18, 21, 24, 29, 34, and 39). The typology of the genera within Hecamedini, including subgenera within *Allotrichoma*, forms a stepwise hierarchy, beginning with *Diphuia* as the sister group to all other genera (see Mathis and Marinoni 2010, for a review and further discussion of *Diphuia*), followed by the subgenera within the genus *Hecamede* (see Mathis 1993 for a revision and further discussion of *Hecamede*). The next lineage in the hierarchy is *Elephantinosoma*, an Old World genus (see Mathis and Deeming 1987, for a revision and further discussion of *Elephantinosoma*.), followed by *Eremotrichoma*, which is likewise an Old World genus. *Eremotrichoma*, which we continue to accord generic status following recent precedent, now includes six species (Mathis 1986a, Canzoneri and Vienna 1989, Krivosheina 1992).

All further lineages (from *Neotrichoma* to the end), which are the more derived lineages, are interpreted to be the genus *Allotrichoma* with the names of taxa denoting subgenera and species groups. This follows the precedent of Mathis (1991) and Mathis and Zatwarnicki (1995). *Neotrichoma*, which is a new subgenus being proposed herein, is the first lineage within *Allotrichoma* and is the sister-group to the remaining subgenera and species groups. The monophyly of this *Neotrichoma* is well established, as is its immediate sister-group, the node giving rise to the subgenera *Pseudohecamede* (see Mathis 1991, for a revision and further discussion of *Pseudohecamede*) and *Allotrichoma*. Likewise, note that *Pseudohecamede* and *Allotrichoma* are well established and well documented subgenera.

In parenthetic notation, the relationships on the cladogram are as follows: (*Discocerina*, ((*Lipochaeta*, *Glenanthe*), (*Diphuia*, ((*Hecamede albicans*, *Hecamede nuda*), (*Elephantinosoma chnumi*, (*Eremotichoma perspiciendum*, (*Neotrichoma atrilabre*, ((*Pseudohecamede abdominale*, *Pseudohecamede salubre*), (*Allotrichoma simplex* group, (*Allotrichoma laterale* group, (*Allotrichoma dyna* group, *Allotrichoma alium* group))))))))))).

**Table 2.** Analysis of characters based on the cladogram (Figure 2).

Characters	1	2	3	4	5	6	7	8	9	10	11	12	13	
Steps	2	2	7	1	0	4	3	1	1	1	6	3	1	
Consistency index	100	100	28	100	100	50	33	100	100	100	66	100	100	
Retention index	100	100	16	100	100	0	33	100	100	100	71	100	100	
Characters	14	15	16	17	18	19	20	21	22	23	24	25	26	
Steps	1	3	3	4	5	4	1	4	1	3	4	2	2	
Consistency index	100	66	33	50	60	25	100	25	100	33	50	100	50	
Retention index	100	0	33	0	0	25	100	40	100	33	60	100	50	
Characters	27	28	29	30	31	32	33	34	35	36	37	38	39	
Steps	1	0	1	2	2	3	2	2	0	2	2	5	6	
Consistency index	100	100	100	50	50	33	50	50	100	50	50	40	50	
Retention index	100	100	100	0	0	50	66	50	100	0	66	66	72	
Characters	40	41	42	43	44	45	46	47	48	49	50	51	52	53
Steps	2	1	1	4	3	5	4	3	2	3	6	2	1	1
Consistency index	50	100	100	75	100	100	75	66	50	100	100	100	100	100
Retention index	0	100	100	0	100	100	83	75	0	100	100	100	100	100

**Table 3.** Weights (1–10) and status (i.e., nonadditive -; inactive ]) of characters after successive weighing.

Character No.	1	2	3	4	5	6	7	8	9	10	11
Weight, status	10-[	10-[	0-[	10-[	10+[	0-[	1+[	10+[	10-[	10+[	4-[
Character No.	12	13	14	15	16	17	18	19	20	21	22
Weight, status	10-[	10+[	10+[	0-[	1+[	0-[	0-[	0+[	10+[	1+[	10+[
Character No.	23	24	25	26	27	28	29	30	31	32	33
Weight, status	1+[	3+[	10-[	2+[	10+[	10+[	10+[	0+[	0-[	1+[	3-[
Character No.	34	35	36	37	38	39	40	41	42	43	44
Weight, status	2-[	10+[	0+[	3+[	2+[	3+[	0-[	10+[	10+[	0-[	10-[
Character No.	45	46	47	48	49	50	51	52	53		
Weight, status	10-[	6-[	5-[	0+[	10-[	10-[	10-[	10+[	10+[		

#### Key to Genera of Hecamedini Mathis

**Table:** 

1	Oral opening large, gaping; only reclinate fronto-orbital seta present; anteroventral margin of face essentially flat, at same level with rest of oral margin; clypeus broad; katepisternal seta lacking	*Elephantinosoma* Becker
–	Oral opening narrow; usually both a reclinate and proclinate fronto-orbital seta present; anteroventral margin of face emarginate with narrow clypeus exposed within facial emargination; katepisternal seta usually present	2
2	Scutellum bearing 3 marginal setae; postgenal margin sharp; gena high, over 1/2 eye height	*Hecamede* Haliday
-	Scutellum bearing 2 marginal setae; postgenal margin rounded; gena short, less than 1/2 eye height	3
3	Palpus mostly yellow; prescutellar acrostichal setae greatly reduced or absent; 1 katepisternal seta; face with 1 large lateral seta	*Eremotrichoma* Giordani Soika
–	Palpus blackish; 1 pair of prescutellar acrostichal setae, well developed; 2 katepisternal setae, the 2nd seta smaller and inserted below larger seta; face with 2 or more large lateral setae	4
4	Body color generally black; microtomentum sparse, subshiny to shiny	*Diphuia* Cresson
–	Body color generally gray to brown; microtomentum dense, generally appearing dull	*Allotrichoma* Becker

### Genus Allotrichoma

Becker

http://species-id.net/wiki/Allotrichoma

Allotrichoma Becker 1896: 121 [type species: *Hecamede lateralis* Loew, by original designation]; 1926: 19 [revision, Palearctic species]. Aldrich 1905: 624 [Nearctic catalog]. Jones 1906: 181 [catalog]. Williston 1908: 307 [generic key]. Hendel 1930: 135–136 [comparison with *Discocerina* and *Hecamede*]; 1936: 102, 104 [comparison with *Discocerina*]. Cresson 1942: 108–109 [review of Nearctic fauna]. Wirth and Stone 1956: 467 [species, California]. Wirth 1965: 736 [Nearctic catalog]; 1968: 5 [Neotropical catalog]. Cole 1969: 397 [discussion, species of western United States]. Mathis 1986a: 129–130 [description]; 1991: 6 [description and discussion]. Mathis and Zatwarnicki 1995: 149–156 [world catalog].

#### Diagnosis.

*Allotrichoma* is distinguished from other genera of the tribe Hecamedini by the following combination of characters: small to moderately small shore flies, body length 1.15–2.95 mm.

*Head*: Wider than high in anterior view; frons wider than high, entirely and mostly densely microtomentose, with vestiture of mesofrons undifferentiated except by color; ocellar setae lacking; 1 well-developed pair of intrafrontal setae inserted in front of anterior ocellus; a reclinate fronto-orbital seta and a proclinate fronto-orbital seta present, reclinate seta inserted slightly anteromediad of proclinate seta; pseudopostocellar setae present, usually well developed; both inner and outer vertical setae present; ocelli in isosceles triangle, with distance between posterior pair slightly larger than between anterior ocellus and either posterior ocellus. Antenna exerted; aristal length subequal to antennal length and bearing 4–6 dorsal rays, with basal 3–4 rays longer than apical 1–2, subequal. Eye apparently bare of microsetulae (using stereomicroscope). Face with dorsal ½-3/4 carinate between antennae; ventral margins curved inward laterally making oral margin narrow, width subequal to narrowest distance between eyes, anterior margin shallowly emarginate; bearing 2 facial setae, the dorsal one very slightly larger, both inserted near parafacials; labella broad, fleshy, shorter than mediproboscis.

*Thorax*: Dorsal portion of anepisternum darker colored than medial portion, frequently concolorous with dorsal coloration of scutum, these areas separated by lighter colored band through postpronotal and notopleural areas; chaetotaxy conspicuous, setae dark colored, arranged in well-defined setal tracks as follows: acrostichal setulae in 4 rows, 2 medial rows better developed, 2 lateral rows attenuated anteriorly; dorsocentral track terminated posteriorly with larger seta; intra-alar setulae irregularly seriated; 1 postpronotal seta; 1 postalar seta; 2 scutellar setae and with sparse, scattered setulae on scutellar disc; 2 notopleural setae, insertion of posterior one elevated dorsally above anterior one; 2 anepisternal setae along posterior margin. Wing with vein R_2+3_ extended beyond level of crossvein dm-cu, 2nd costal section at least 1.5X longer than 3rd section.

*Abdomen*: Fifth tergite of male not visible from a dorsal view, telescoped within 4th.

#### Natural History.

Adults generally feed on nectar from different flowering-plant species and oviposit upon various kinds of decomposing organisms and excrement (Deonier, in litt.).

#### Discussion. 

The precedent of an enlarged concept of *Allotrichoma*, including *Pseudohecamede* Hendel as a subgenus (Mathis 1986a, 1988, 1991, 1993), is adhered to here.

For purposes of agreement between species-group names and genus-group names, the generic name *Allotrichoma* is neuter, not feminine.

The following is a key to the subgenera presently included in the genus.

#### Key to Subgenera of *Allotrichoma*

**Table:** 

1	Medial facial carina above facial prominence shallow; presutural supra-alar seta present; labella broad, fleshy, shorter than mediproboscis	2
–	Medial facial carina above facial prominence distinct, high, acute; presutural supra-alar seta lacking (except in *Allotrichoma slossonae*); labella lanceolate, elongate, nearly equal to length of mediproboscis	Subgenus *Pseudohecamede* Hendel
2	Facial microtomentum cinereous; clypeus mostly bare, black, subshiny. Tergite 5 of ♂ short, less than 1/2 length of 4th; sternite 5 unmodified; cerci unmodified	*Neotrichoma*, subgen. n.
–	Facial microtomentum golden brown; clypeus microtomentose, dull. Tergite 5 of ♂ long, subequal in length to 4th; sternite 5 modified bearing a ventral process; cerci modified, usually elongate	Subgenus *Allotrichoma* Becker

### Subgenus Allotrichoma

Becker

http://species-id.net/wiki/Allotrichoma

Allotrichoma Becker 1896: 121 [type species: *Hecamede lateralis* Loew, by original designation]. Hendel 1936: 104 [discussion]. Wirth 1965: 736 [Nearctic catalog]. Runyan and Deonier 1979: 123-137 [discussion, natural history, phylogenetic analysis]. Mathis 1986a: 129-130 [diagnosis and discussion]; 1991: 6–7 [diagnosis]. Mathis and Zatwarnicki 1995: 149–154 [world catalog]. Also see generic synonymy.
Epiphasis Becker 1907: 301 [type species: *Epiphasis clypeata*Becker 1907, monotypy]. Cogan 1984: 131 [synonymy].

#### Diagnosis.

Small to moderately small shore flies, body length 1.15-2.40 mm.

*Head*: Frons mostly unicolorous, at most with narrow, anterior fronto-orbits slightly lighter in color, lacking distinctively colored ocellar triangle; pseudopostocellar setae subequal in length to intrafrontal setae. Pedicel with well-developed, proclinate, dorsal seta. Facial coloration sexually dimorphic, males unicolorus and darker; face with dorsal 2/3 between antennal grooves shallowly carinate, becoming more prominent ventrad of antennal grooves, slightly tuberculate; clypeus usually mostly microtomentose, dull colored; palpus blackish.

*Thorax*: Mesonotum generally dark brown; chaetotaxy generally well developed; prescutellar acrostichal setae much larger than other acrostichal setae and more widely set apart; presutural seta well developed, length subequal to notopleural setae; katepisternum with 2 setae, 2nd seta smaller and inserted below larger seta. Wing mostly milky white; veins behind costa brownish; alular marginal setulae short, less than 1/2 alular height. Legs: tibiae dark, concolorous with femora; male hindtibia bearing 3-4 semierect, preapical setae on dorsum.

*Abdomen*: Fifth tergite tubular, elongate, well sclerotized, length subequal to that of 4th, bearing a posteroventral process in conjunction with 5th sternite; 5th sternite highly modified, produced as a ventral flap or processes. Male terminalia: cerci well sclerotized, elongate, often twice length of epandrium (probably used at least partially as a clasping structure); surstylus fused to ventral margin of epandrium, usually appearing as an elongate extension of the epandrium (secondarily lost in some species), bearing apical setulae; a single gonite (presumably fused pre- and postgonites) sheathing aedeagus; aedeagus simple, tubelike, sometimes slightly arched; hypandrium generally reduced, fused basally to base of gonite, in lateral view appearing strap or bar-like.

#### Distribution.

Except for polar regions and South America, the subgenus *Allotrichoma* occurs worldwide, although with greater diversity in temperate zones (Mathis and Zatwarnicki 1995).

#### Natural history.

Very little is known about the natural history of any species of *Allotrichoma*, especially larval habitats and immature stages. The brief and few references are as follows:

From the island of Guam, Bohart and Gressitt (1951: 85) wrote that “Adults [of apparently *Allotrichoma livens* Cresson] swarm around foul-smelling mud and puddles and are especially abundant in the mud of pig pens around feeding troughs where contamination by garbage and pig feces is heavy. The larvae breed in pig droppings and pupate at the surface of the droppings. Although maggots were not observed in the contaminated mud of pig pens, it is probable that most of them develop there.”

“This fly is extremely common wherever found. It is apparently not present around mud which is free from contamination. It probably has no medical significance, and control is unnecessary.”

Bohart and Gressitt (1951: plate 11) also presented an illustration of the puparium of *Allotrichoma livens*.

Runyan and Deonier (1979: 123-137) summarized information on the natural history of *Allotrichoma* and published the only illustrations on the eggs, puparia, and cephalopharyngeal skeleton of *Allotrichoma (Allotrichoma) simplex* and *Allotrichoma (Pseudohecamede) abdominale* and the egg of *Allotrichoma (Neotrichoma) atrilabre* (as *Allotrichoma yosemite*). They also reported that specimens of these three groups occur in habitats that are rich in decaying organic matter, including limnic wrack, stagnant pools, and vertebrate excrement (muskrat, raccoon, horse, pig, and cow feces). Sand shores and piles of lawn clippings were also added as habitats for adults, which have also been swept from the inflorescences of a wide variety of plant species. Runyan, the senior author of this paper, also collected adults on dead fish and crayfish carcasses.

We too have collected adults from the kinds of habitats reported above but emphasize that much of our success in collecting was on fairly undisturbed, sandy shores associated with both lotic and lentic aquatic systems, sometimes with the adjoining shore having alkali and perhaps other salts. Moreover, we often found several species occurring microsympatrically together. We commonly found two species together and at a few sites in the West, we collected up to five species occurring together. Based on this finding, we have been especially hesitant to designate females as paratypes, not knowing how to distinguish between them, from localities where multiple species occur. Our discovery begs the question about how these various species are partitioning the habitat where and when they occur together microsympatrically.

#### Discussion.

The monophyly of this subgenus is corroborated by several characters that Runyan and Deonier (1979) and more recently that Krivosheina and Zatwarnicki (1997) have identified. These synapomorphies are as follows: (1) Surstylus (=gonostylus; fused to epandrium, therefore called surstylus) as a simple appendix, terminating with apical setulae, or secondarily reduced; (2) gonites incised apically; (3) cerci generally elongate, in some tropical species fused to each other, rarely oval; (4) 5th sternite with a ventrally produced, paired, lateral flap (one flap on each side) and a medial process; (5) 4th tergite of male largely vestigial, forming two small bands close to the anterior margin of 5th tergite.

An objective of this revision was to report the determination of species that may have a Holarctic distribution, as noted in the introduction. For example, among the seven species reported from the Nearctic Region, Wirth (1965) listed two, *Allotrichoma laterale* (Loew) and *Allotrichoma trispinum* Becker, with Holarctic distributions. Wirth also cautioned (1965: 736), however, that “The species are poorly understood and distributions records need revision after a study of male genitalia.” Our research, which has emphasized characters from structures of the male terminalia, indeed reports not two but three Holarctic species, but none is one of the species names that Wirth reported earlier. The Holarctic species reported in this revision are: *Allotrichoma bezzii*, *Allotrichoma schumanni*, and *Allotrichoma simplex*. The first two are species first named from specimens collected in Europe and then discovered here, and the last species, *Allotrichoma simplex*, has as its type locality, “Maryland,” and has now been found to be the senior synonym of taxa occurring in Europe. We call attention to these discoveries to emphasize that studies of limited geographic scope, such as this revision of New World species, need to consider congeners on a global basis to avoid misidentifications and incorrect distributional data.

We can presently identify only males of this subgenus, and as a consequence, we have only used structures of the male terminalia in the key to the New-World species of this subgenus that follows.

#### Key to Males of New-World Species of *Allotrichoma (Allotrichoma)*

**Table:** 

1	Cercus in posterior view with ventral extensions straight to very slightly bowed ventrolaterally (Figs 3, 15, 21), not lyrelike or abruptly bowed laterally and then curved ventrally, producing shoulder-like lateral margins	2
–	Cercus in posterior view with ventral extensions more or less lyrelike (Figs 27, 39, 63, 69), basal shoulders broadly rounded laterally and then curved ventrally	5
2	Cercus in posterior view with ventrolateral extension essentially straight	3
–	Cercus in posterior view with ventral extension very slightly bowed laterally and then ventrally	4
3	Cercus in posterior view with ventral half more or less parallel sided with apex abruptly curved medially, in lateral view with apex angulate, truncate, bearing several long to very long mostly apical setulae (length subequal or greater than cercal length); gonite bearing 3 short setulae, two subapical, the other apical	*Allotrichoma (Allotrichoma) deonieri* Mathis and Zatwarnicki
–	Cercus in posterior view with ventral extension becoming wider toward very broad apex, in lateral view with ventral apex rounded, veering numerous setulae, those at apex especially elongate; gonite with 2 setulae, one subapical, the other apical	*Allotrichoma (Allotrichoma) robustum* sp. n.
4	Cercus with apex rounded (best seen in lateral view); male 5th sternal flap in lateral view (Fig. 5) shallow but long, bearing numerous, tuberculate setae; 5th medial process (Fig. 4) elongate, very shallowly curved, bearing 4–5 short, apical setulae	*Allotrichoma (Allotrichoma) bezzii* Becker
–	Cercus with apex truncate (best seen in lateral view; Fig. 22); male 5th sternal flap in lateral view (Fig. 25) relatively shallow but long, bearing 6–8, tuberculate setae; 5th medial process in lateral view (Fig. 25) robust, greatly enlarged basally, with a posterior projection, broadly pointed apically, bear row of setulae anteroapically	*Allotrichoma (Allotrichoma) dynatum* sp. n.
5	Cercal apex bearing 2–4 short setulae	6
–	Cercal apex bearing numerous, long setulae	7
6	Cercal apex bearing 2 short, apical setulae (Figs 72); surstylus with short, apical, ventral extension (Fig. 69) but not bifurcate; male 5th sternal flap in lateral view (Fig. 70) moderately elongate, bearing 3 long setulae apically; 5th medial process in lateral view (Fig. 70) very elongate, nearly straight, apex widened, bearing 4–5 apical setulae	*Allotrichoma (Allotrichoma) wallowa* sp. n.
–	Cercal apex bearing 4 short, apical setulae (Figs 10); surstylar apex distinctly bifurcate (Fig. 10); male 5th sternal flap in lateral view (Fig. 12) much longer than wide, truncate apically, bearing numerous, apical setae; 5th medial process in lateral view (Fig. 12) elongate, bar-like, bearing 4–5 apical setulae	*Allotrichoma (Allotrichoma) bifurcatum* sp. n.
7	Surstylus comparatively wide, apex broad, truncate*Allotrichoma lacteum* Cresson
–	Surstylus narrow, elongate, apex not truncate	8
8	Extended cercus in posterior view with medial, midheight step, step angulate to slightly produced	9
–	Extended cercus essentially straight medially, lacking midheight step	10
9	Cercus with apical portion spathulate apically, bearing numerous elongate setulae	*Allotrichoma (Allotrichoma) schumanni* Papp
–	Cercal with apical portion parallel sided, not expanded, bearing 4 short setulae apically	*Allotrichoma (Allotrichoma) simplex* (Loew)
10	Surstylus with ventral extension in lateral view comparatively more robustly developed, with shallow, subapical notch and bearing numerous, moderately short setulae (Fig. 34); male 5th sternal flap in lateral view (Fig. 37) pedunculate, relatively narrow at base, becoming wider apically, bearing numerous setulae along truncate apex; 5th medial process in lateral view (Fig. 37) elongate, bar-like, bearing 4–5 apical setulae	*Allotrichoma (Allotrichoma) lasiocercum* Cresson
–	Surstylus with ventral extension in lateral view narrowly and thinly developed to apex, bearing only very short, apical setulae; male 5th sternal flap in lateral view narrow, elongate, slightly widened apically, bearing 5–6 apical setulae, some from short tubercles; 5th medial process in lateral view relatively narrow, very elongate, apex slightly widened, bearing 4–5 setulae along anteroapical surface	11
11	Cercus in lateral view comparatively more robustly developed to apex and bearing numerous setulae along length (Figs 51–53); surstylar extensions straight (Fig. 53)	*Allotrichoma (Allotrichoma) sabroskyi* sp. n.
–	Cercus in lateral view comparatively more thinly developed along most of length, apex moderately flared, with moderately long length from midheight to apex without setulae (Fig. 40); surstylar extensions slight curved laterally in posterior view (Fig. 39)	*Allotrichoma (Allotrichoma) occidentale* sp. n.

### Allotrichoma (Allotrichoma) bezzii

Becker

http://species-id.net/wiki/Allotrichoma_bezzii

Figs 1 3 8

Allotrichoma bezzii Becker 1896: 123 [Italy]; 1905: 189 [Palearctic catalog]. Zatwarnicki 1991: 298–301 [lectotype designation; illustration of male terminalia]. Mathis and Zatwarnicki 1995: 150 [world catalog]. Krivosheina and Zatwarnicki 1997: 295–297 [revision].
Allotrichoma laterale of authors, not Loew 1860 [misidentification in part]. Becker 1896: 121 [generic combination]. Williston 1897: 4 [compared with *Allotrichoma abdominale* Williston]. Cresson 1942: 108 [list, west coast of United States]. Wirth and Stone 1956: 467 [key]. Wirth 1965: 736 [Nearctic catalog, in part]. Cole 1969: 397 [list]. Mathis and Zatwarnicki 1995: 151–152 [world catalog, in part].

#### Description.

This species is distinguished from congeners by the following combination of characters: Small to moderately small shore flies, body length 1.35–2.30 mm (Fig. 1). *Head*: Medial facial carina above facial prominence shallow; labella broad, fleshy, shorter than mediproboscis; clypeus microtomentose, usually gray.

*Thorax*: Presutural supra-alar seta present. Wing with costal vein ratio 0.27–0.33; M vein ratio 0.38–0.44.

**Figures 3–7.**
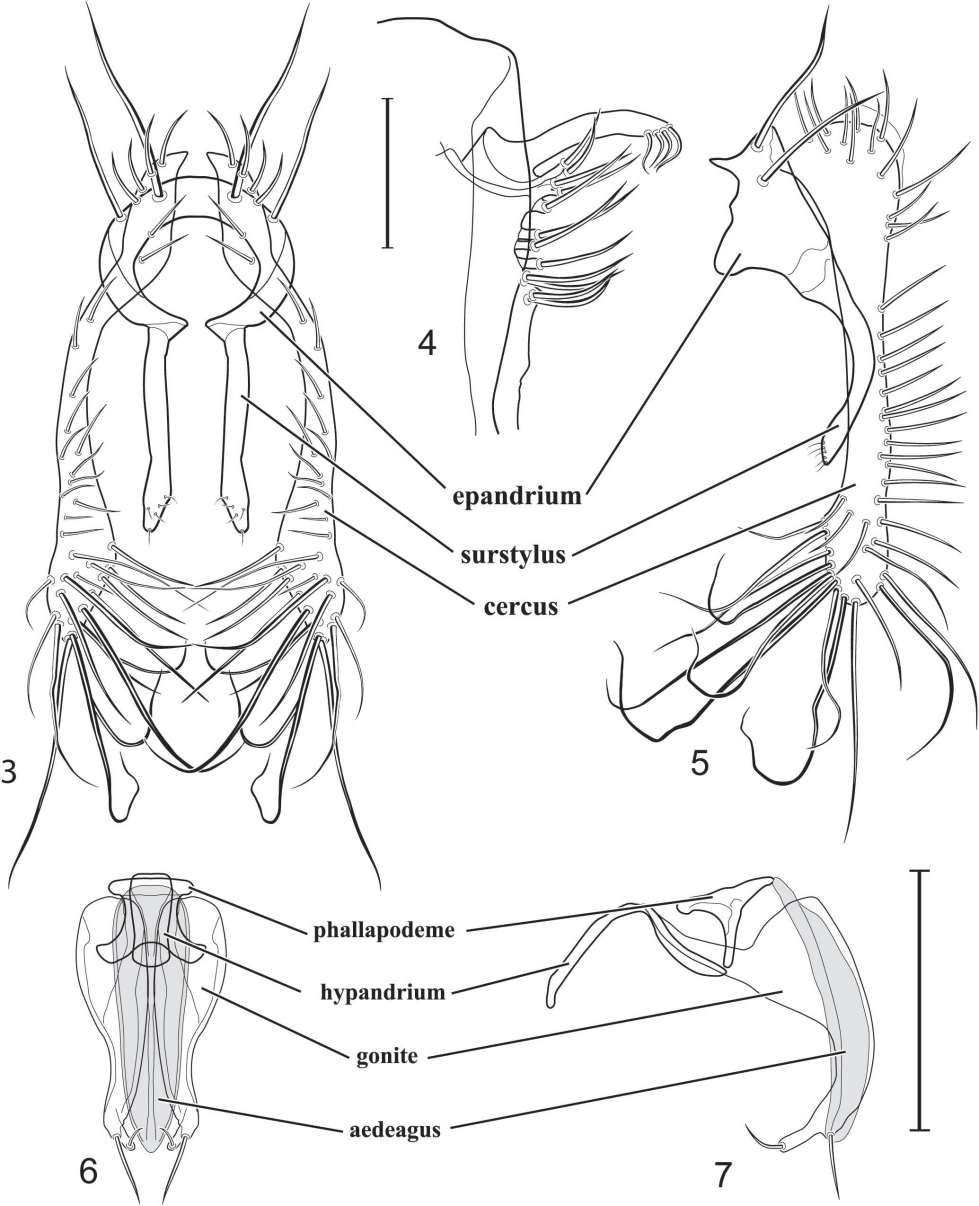
Illustration of *Allotrichoma bezzii* Becker (male) **3** epandrium, cerci, surstylus, posterior aspect **4** male 5th sternal flap and medial process, lateral aspect **5** epandrium, cerci, surstylus, lateral aspect **6** aedeagus, phallapodeme, gonite, hypandrium, ventral aspect **7** same, lateral aspect. Scale bar = 0.1 mm.

*Abdomen*: Male 5th sternal flap in lateral view (Fig. 4) shallow but long, bearing numerous, tuberculate setae; 5th medial process (Fig. 4) elongate, shallowly curved, bearing 4–5 setulae apically. Male terminalia (Figs 3, 5–7): Epandrium in posterior view (Fig. 3) like an inverted, laterally rounded, almost oval U; epandrium in lateral view (Fig. 5) relatively narrow dorsally, becoming wider ventrally; cercus in lateral view (Fig. 5) essentially straight, widest dorsally, then narrowed but gradually becoming wider ventrally, ventral apex narrowly spatulate, long and apically curved setulae especially evident on ventral 1/4; cercus in posterior view (Fig. 3) as a elongate, pendulous, ventrally projected process, gradually becoming wider ventrally, apex irregularly spatulate (slightly curved laterally), bearing numerous, very long setulae along length and especially long at apical 1/4; surstyli (ventral extensions of epandrium) in posterior view as parallel, narrow processes that bear setulae on apical portion, apical portion very slightly broadened and turned laterally; surstylus in lateral view narrow, elongate, gently curved, apical portion not expanded, bearing setulae apically; aedeagus in ventral view (Fig. 6) elongate, narrowly ovate, slightly tapered apically, in lateral view (Fig. 7) elongate, shallowly and narrowly lunate; phallapodeme in lateral view (Fig. 6) narrowly triangular, keel shorter than length, almost digitiform; gonite in ventral view (Fig. 7) somewhat bar-like, lateral margin shallowly curved, especially basally and apically, with short, subapical process, apex of process bearing a setula, with a subapical, distinct, angulate notch and bearing a subapical setula; gonite in lateral view (Fig. 7) wide basally, gently curved and tapered toward apex, with a subapical, short process that bears a setula and an apical setula.

#### Type material.

Although Zatwarnicki (1991: 298) designated a lectotype for this species, that designation is invalid, not being based on a syntype. According to Becker’s diary (ZMHU), the specimen selected as the lectotype was collected in 1897, a year after publication of this species (1896), and therefore, it could not be a syntype. We and others (Maurizio Pavesi, MSNM) have searched for the type series of this species in Becker and Bezzi’s collections without success. Thus to stabilize the nomenclature of this species, we have determined that a neotype should be designated and have selected the so-called lectotype as the neotype. The neotype is labeled “[Sweden.] Edefors 1/7. 43265 [handwritten]/Allotrichoma bezzii Beck. ♂ det. Papp L[ászló]/NEOTYPE ♂ *Allotrichoma bezzii* Becker by Mathis & Zatwarnicki, ZMHU [red].” The neotype is double mounted, is in generally good condition (abdomen removed and dissected; structures in an attached microvial), and is deposited in the ZMHU.

#### Type locality.

Sweden. Norrbotten: Edefors (66°13'N, 20°54'E).

#### Other specimens examined from the New World.

*CANADA. Saskatchewan*. Waskesiu Lake (53°55'N, 106°05'W), Aug 1947, R. Coleman (1♂; USNM).

*MEXICO. Baja California*: Sierra San Pedro Martir (31°02.7'N, 115°28'W), 15 Jun 1953, P. H. Arnaud, Jr. (3♂; CAS). *Jalisco*: Jalisco (2.8 mi E; Lagos de Moreno; 21°12.6'N, 101°33'W), 27 Jul 1962, N. Marston (1♂; USNM).

*UNITED STATES. ALASKA*. Mirror Lake (61°25.7'N, 149°24.9'W), 29 Jun 2006, D. and W. N. Mathis (5♂, 4♀; USNM). *Kenai Peninsula*: Arc Lake (3.2 km W Soldotna; 60°27'N, 151°06.3'W), 5 Jul 2006, D. and W. N. Mathis (1♂, 7♀; USNM); Kenai Lake (60°20.5'N, 149°22.2'W; Primrose Campground), 31 Jul 2002, D. and W. N. Mathis (3♂, 3♀; USNM); Skilak Lake (60°26.3'N, 150°19.4'W), 3 Aug 2002, D. and W. N. Mathis (8♂, 13♀; USNM); Soldotna (6.5 km E; 60°30.5'N, 150°55.6'W), 1 Aug 2003, D. and W. N. Mathis (1♂, 2♀; USNM). *Matanuska-Susitna*: Talkeetna (62°18.9'N, 150°06.3'W), 4 Aug 2003, D. and W. N. Mathis (1♂, 2♀; USNM); Willow Creek (61°46.1'N, 150°04.2'W; 50 m), 10–26 Jul 2006, 2011, D. and W. N. Mathis (7♂, 8♀; USNM).

*ARIZONA. Coconino*: Globe (33°23.6'N, 110°47.2'W), 13 Apr 1935, A. L. Melander (1♂; USNM). *Cochise*: Southwestern Research Station (8 km W Portal; 31°52.9'N, 109°12.2'W), P. H. Arnaud (1♂; CAS). *Maricopa*: Gila Bend (47 km E; 32°57'N, 112°13'W), 15 Aug 1964, E. I. Schlinger (1♂; UCR); Tempe (33°24.9'N, 111°54.6'W), 11 May 1942, A. L. Melander (1♂; USNM). *Navajo*: Kayenta (36°44.4'N, 110°14.4'W), 14 Apr 2003, W. N. Mathis, T. Zatwarnicki (1♂; USNM). *Santa Cruz*: Nogales (31°20.4'N, 110°56.1'W), 28 Jun 1953, W. W. Wirth (1♂; USNM). *Yuma*: Yuma (32°43.5'N, 114°37.5'W), Oct 1953 (2♂, 2♀; USNM).

*CALIFORNIA. Kern*: Rosamond (34°51.8'N, 118°9.7'W), 17 Oct 1956, A. H. Sturtevant (5♂; USNM). *Lake*: Clear Lake (39°03.7'N, 122°49.6'W), 18 Jun 1935, A. L. Melander (1♂; ANSP). *Los Angeles*: San Gabriel Mountains, King Creek (34°17.3'N, 117°38.8'W), A. L. Melander (1♂; USNM). *Orange*: Irvine lake (33°47.1'N, 117°43.5'W), 11 Sep 1963, M. E. Irwin (1♂; UCR). *Riverside*: Deep Creek (34°17.2'N, 117°7.6'W), 25 Oct 1953, A. L. Melander (1♂; USNM); Magnesium Spring Canyon (near Indio; 33°43.2'N, 116°13'W), 5 Apr 1945, A. L. Melander (1♂; USNM). *San Bernardino*: Jenks Lake (34°09.9'N, 116°52.9'W), 16 Jul-18 Aug 1950, 1958, A. L. Melander (4♂, 2♀; USNM); Lake Gregory (34°14.8'N, 117°16.1'W), 6 Jul 1965, R. E. Orth (1♂; UCR); Mountain Home Canyon (34°06'N, 117°0.1'W), 4 Sep-8 Oct 1952, 1955, 1956, A. L. Melander (1♂, 4♀; USNM); South Fork, Santa Ana River (34°10'N, 116°48.8'W), 29 Jul 1942, A. L. Melander (1♂; USNM); Twenty-nine Palms (34°08.1'N, 116°03.3'W), 6 Oct 1949, A. L. Melander (1♂; USNM). *San Diego*: El Cajon (32°47.6'N, 116°57.7'W), 9 Aug 1944, A. L Melander (1♂; USNM).

*COLORADO. Archuleta*: Pagosa Springs (37°15.8'N, 107°00.7'W), 27 May 1969, W. W. Wirth (1♂; USNM). *Boulder*: Boulder (11 km N, 40°11.6'N, 105°14.7'W; 1525 m), 6 Jul 1986, R. Danielsson (1♂; ZIL). *Custer*: Wetmore (16 km SW; 38°14'N, 105°06'W), 8 Aug 1973, G. S. and S. Hevel (1♂; USNM). *Dolores*: Cahone (9 km N, Dolores River; 37°38.9'N, 108°44.1'W; 1955 m), 3 Aug 2007, D. and W. N. Mathis (3♂, 1♀; USNM). *Gunnison*: Crested Butte (38°52.8'N, 106°59.4'W; 2682 m), 8 Jul-5 Aug 1957, A. H. Sturtevant (3♂; USNM); Gothic (38°57.5'N, 106°59.4'W; 2895 m), 20-28 Jul 1957, A. H. Sturtevant (3♂; USNM); Rocky Mountain Biological Laboratory (38°57.5'N, 106°59.4'W; 2895 m), 26 Aug 1961, D. L. Deonier (1♂; USNM).

*IDAHO. Cassia*: Black Pine Mountains (42°09.3'N, 112°58.3'W), 29 Sep 1969, G. F. Knowlton (1♂; USU). *Oneida*: Curlew National Grassland (42°12.5'N, 112°45'W), 29 Jul 1969, G. F. Knowlton (1♂, 2♀; USU).

*MINNESOTA. Lake*: Basswood Lake (48°04.5'N, 91°34.5'W), 12 Jul-16 Aug 1950, E. F. Cook, J. W. Barnes (2♂, 1♀; USNM).

*MONTANA. Lake*: Swan Lake (4.8 km S; 47°55.7'N, 113°50.7'W), 10 Aug 1968, B. A. Foote (1♂; USNM).

*NEVADA. Lincoln*: Alamo (6.5 km S; 37°19'N, 115°10'W), 17 May 2004, D. Mathis (2♂, 1♀; USNM). *Nye*: Ash Meadows National Wildlife Refuge (36°26.7'N, 116°21'W), 12 May 2001, D. Mathis (6♂, 4♀; USNM). *Washoe*: Galena Creek (39°21.3'N, 119°46.1'W), 18 Jul 1961, F. D. Parker (1♂; USNM).

*NEW MEXICO. Catron*: Gila River (33°13.6'N, 106°15.1'W; 1750 m, near Gila Cliff Dwellings National Monument), 15 Aug 2007, D. and W. N. Mathis (4♂; USNM). *Chaves*: Roswell (9 km E; Pecos River; 33°23.8'N, 104°24'W; 1060 m), 10 Aug 2007, D. and W. N. Mathis (1♂; USNM). *Dona Ana*: Las Cruces (32°18.7'N, 106°46.7'W), 17 Jun 1950, L. D. Beamer (1♂; USNM). *Grant*: Bill Evans Lake (32°52.1'N, 108°34.5'W; 1416 m), 14 Aug 2007, D. and W. N. Mathis (1♂; USNM); Mimbres River (NM Highways 61 and Royal John Mine Road; 32°43.8'N, 107°52'W; 1665 m), 13-22 Aug 2007, 2009, D. and W. N. Mathis, T. Zatwarnicki (3♂; USNM). *Sandoval*: La Cueva (Junction of Highways 126 and 4; 35°52'N, 106°38.4'W; 2342 m), 6 Aug 2007, D. and W. N. Mathis (11♂; USNM); Las Conchas (fishing access and picnic area, 17 km N La Cueva; 35°48.9'N, 106°31.5'w; 2550 m), 7 Aug 2007, D. and W. N. Mathis (1♂; USNM); Valles Caldera National Preserve (E Fork Jemez River; 35°51'N, 106°29.5'W; 2580 m), 6 Aug 2007, D. and W. N. Mathis (1♂; USNM); Valles Caldera National Preserve, Alamo Canyon (middle pond; 35°54.9'N, 106°35'W; 2645 m), 4 Aug 2008, D. and W. N. Mathis (3♂; USNM). *San Miguel*: Las Vegas (35°35.6'N, 105°13.4'W), 26 Jun 1973, W. N. Mathis (1♂; USNM). *Valencia*: Los Lunas (34°48.4'N, 106°44'W), 17-22 Jun 1963, J. G. Watts (2♂, 3♀; USNM); Rio Puerco (34°47.8'N, 106°59.5'W; 1575 m), 9 Aug 2007, D. and W. N. Mathis (3♂; USNM).

*NORTH DAKOTA. Bowman*: Bowman-Haley Dam and Reservoir (45°59.5'N, 103°15.4'W; 840 m), 19 Jun 2008, D. and W. N. Mathis (1♂; USNM).

*OREGON. Baker*: Goose Creek (35 km E Baker City; 44°49.2'N, 117°27.79'W; 825 m), 7 Jun 2006, D. and W. N. Mathis (1♂; USNM). *Harney*: Pikes Creek (42°34.5'N, 118°31.7'W; 1320 m), 7 Aug 2005, D. and W. N. Mathis (16♂, 6♀; USNM). *Jackson*: Trail (42°38.9'N, 122°48.7'W), 10 Aug, A. H. Sturtevant (1♂; USNM). *Josephine*: Grants Pass (42°26.3'N, 123°19.7'W), 4 Aug 1952, H. A. Scullen (1♂; OSU). *Linn*: Monument Peak (44°41.7'N, 122°19.3'W), 12 Jul 1972, W. N. Mathis (1♂; USNM).

*TEXAS. Bexar*: Camp Stanley (29°41.7'N, 98°37'W), Oct 1954 (1♂; USNM). *Pecos*: Bakersfield (30°53.5'N, 102°17.9'W), 19 May 1950, A. H. Sturtevant (1♂; USNM).

*UTAH. Cache*: Beaver Mountain (41°52.9'N, 111°33.5'W), 13 Jul 1945, G. F. Knowlton (1♂; USNM). *Carbon*: Deadman Canyon (16 km NE Price; 39°41.7'N, 110°44'W; 2055 m), 14-19 Aug 2008, 2009, D. and W. N. Mathis, T. Zatwarnicki (4♂; USNM). *Duchesne*: Roosevelt (40°18'N, 109°59.3'W), 29 Jun 1954, G. F. Knowlton (1♂; USNM). *Emery*: Green River (3.3 km N, 39°01.7'N, 110°09.7'W; 1253 m), 30 Jul 2007, D. and W. N. Mathis (4♂; USNM); San Rafael River (22.5 km SW Green River; 38°55.7'N, 110°24.5'W; 1270 m), 31 Jul 2007, D. and W. N. Mathis (4♂; USNM). *Grand*: Crystal Geyser (14.5 km SE Green River; 38°56.3'N, 110°08.1'W), 15 Aug 2008, D. and W. N. Mathis (4♂; USNM); Swasey Beach (15.3 km N Green River; 39°07'N, 110°06.6'W; Green River; 1255 m), 29 May-15 Aug 2007, 2008, D. and W. N. Mathis (4♂; USNM); Thompson Spring (8.9 km N Thompson Springs; 39°02.3'N, 109°43.4'W; 1740 m), 20 Aug 2009, W. N. Mathis, T. Zatwarnicki (2♂; USNM). *Kane*: Drip Tank Canyon (37°19.4'N, 111°31.8'W), 15 May 2001, D. and W. N. Mathis (1♂; USNM). *Millard*: Delta (39°21.1'N, 112°34.6'W), 14 Aug 1940, L. J. Lipovsky (1♂, 1♀; USNM); Delta (4.8 km W; 39°19.4'N, 111°34.4'W), 21 Jul 1968, W. N. Mathis (4♂, 11♀; USNM). *Piute*: Marysvale (38°27'N, 112°13.8'W), 19 Sep 1952, A. H. Sturtevant (1♂; USNM). *Salt Lake*: Butterfield Canyon (40°29.2'N, 112°08.2'W; 1890 m), 13 Aug 2008, D. and W. N. Mathis (3♂; USNM); Draper (40°31.6'N, 111°55.1'W; Jordan River; 1320 m), 10 May 2007, D. and W. N. Mathis (6♂; USNM). *Uintah*: Lapoint (40°24.2'N, 109°47.6'W), 29 Jun 1954, G. F. Knowlton (1♂, 4♀; KU); Vernal (40°27.3'N, 109°31.7'W), 17 Jul 1952, G. F. Knowlton (1♂; USNM); Vernal Mountains (40°29.5'N, 109°27'W), 17 Jul 1952, G. E. Bohart, G. F. Knowlton (5♂; USNM). *Utah*: American Fork Springs (40°22.6'N, 111°47.7'W), 8 May 2001, R. W. Baumann, I. Winkler (3♂, 1♀; BYU); Goshen Springs (39°57.8'N, 111°51.2'W), 16 Apr 2003, T. Zatwarnicki, W. N. Mathis (6♂; USNM).

*WASHINGTON. Benton*: Plymouth (12 km W; 45°56.1'N, 119°24.6'W), 9 Jul 1978, R. S. Zack (1♂; WSU). *Mason*: Lilliwaup (47°28'N, 123°07'W), 23 Jul 1917, A. L. Melander (1♂, 2♀; ANSP). *Pierce*: Sunrise Point, Mount Rainier National Park (1.6 km W; 46°55.2'N, 121°35.2'W), 22 Aug 1973, W. J. Turner (1♂; WSU).

*WYOMING. Carbon*: Elk Mountain (5 km W; 41°41.1'N, 106°26.4'W), 20 Aug 1982, R. S. Zack (8♂; WSU).

**Figure 8.**
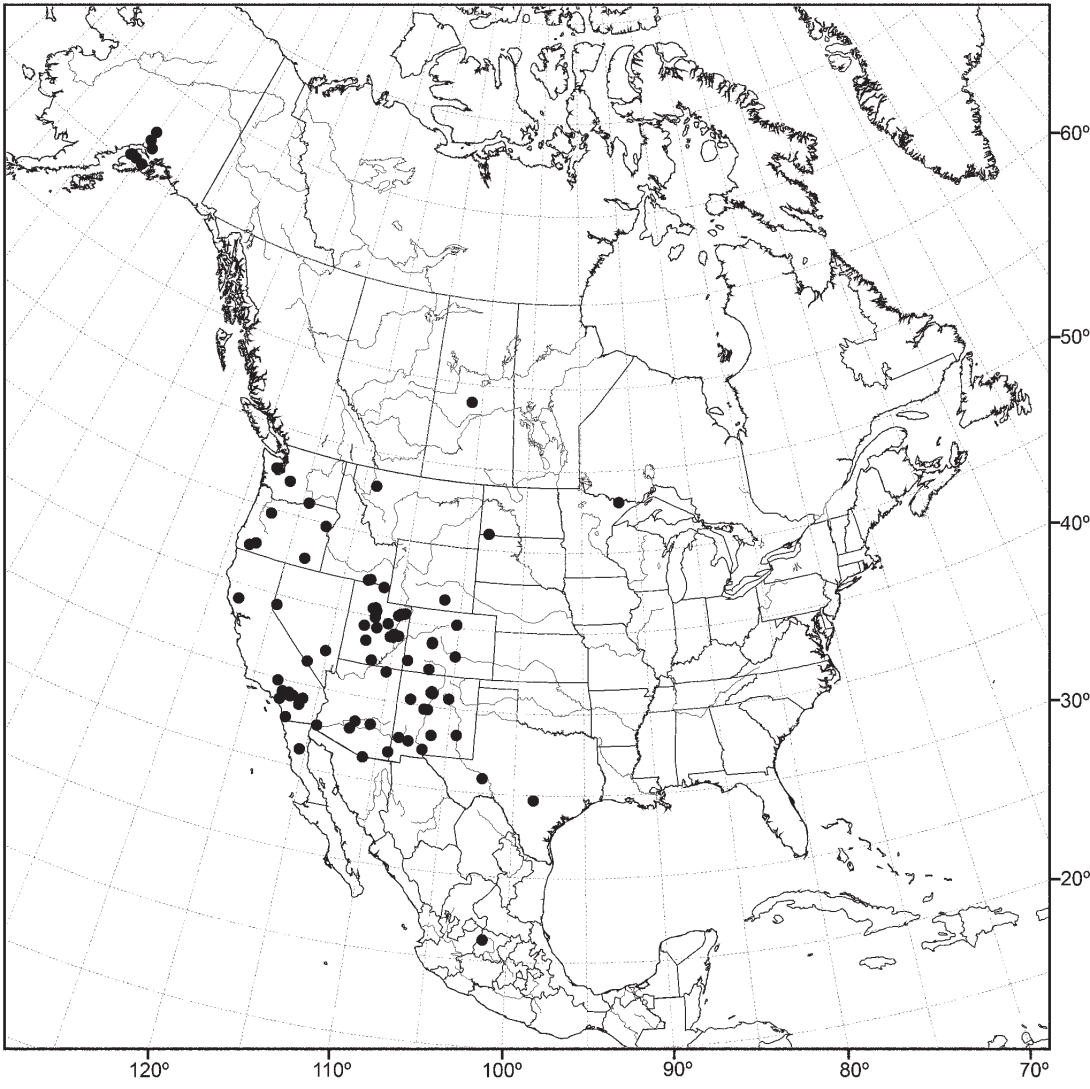
Distribution of *Allotrichoma bezzii* Becker.

**Distribution (Fig. 8).** *Nearctic*: Canada (Saskatchewan), Mexico (Baja California, Jalisco), United States (Alaska, Arizona, California, Colorado, Idaho, Minnesota, Montana, Nevada, New Mexico, North Dakota, Oregon, Texas, Utah, Washington, Wyoming). Palearctic: Afghanistan, Austria, Bulgaria, Croatia, France, Germany, Hungary, Italy, Lithuania, Poland, Russia (European Territory), Slovenia, Spain, Sweden, Yugoslavia.

**Remarks.** In the Nearctic Region, this species has been found thus far only in the West, including Alaska, and the locality data suggest that the species probably also occurs in the western provinces of Canada.

As noted in the synonymy, many previous records of this species occurring in the New World were cited as *Allotrichoma laterale*, a common misidentification. In the Palearctic Region, Zatwarnicki (1991) and Mathis and Zatwarnicki (1995) listed two European species as being conspecific with *Allotrichoma bezzii*: *Allotrichoma lena* Dahl (1973: 354: Afghanistan. Herat: Bala Murghab (470 m); HT ♂) and *Allotrichoma pedemontanum* Canzoneri and Meneghini (1979: 629: Italy. fiume Po a Torino; HT ♂). We confirm their conspecificity here and thus, that these names are synonyms.

### Allotrichoma (Allotrichoma) bifurcatum sp. n.

urn:lsid:zoobank.org:act:4EA5F0F3-54A0-4A71-A7EB-113C061B3BC7

http://species-id.net/wiki/Allotrichoma_bifurcatum

Figs 9 14

#### Description.

This species is distinguished from congeners by the following combination of characters: Small shore flies, body length 1.35–1.85 mm. *Head*: Medial facial carina above facial prominence shallow; labella broad, fleshy, shorter than mediproboscis; clypeus microtomentose, usually gray.

*Thorax*: Presutural supra-alar seta present. Wing with costal vein ratio 0.27–0.29; M vein ratio 0.47–0.50.

**Figures 9–13.**
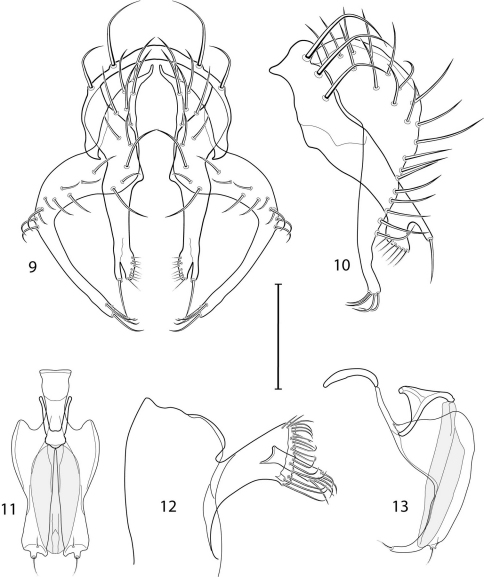
Illustration of *Allotrichoma bifurcatum* sp. n. (male) **9** epandrium, cerci, surstylus, posterior aspect **10** same, lateral aspect **11** aedeagus, phallapodeme, gonite, hypandrium, ventral aspect **12** male 5th sternal flap and medial process, lateral aspect **13** aedeagus, phallapodeme, gonite, hypandrium, lateral aspect. Scale bar = 0.1 mm.

*Abdomen*: Male 5th sternal flap in lateral view (Fig. 12) much longer than wide, truncate apically, bearing numerous, apical setae; 5th medial process in lateral view (Fig. 12) elongate, bar-like, bearing 4–5 apical setulae. Male terminalia (Figs 9–11, 13): Epandrium in posterior view (Fig. 9) like an inverted U with short, apical medial flanges ventrally; epandrium in lateral view (Fig. 10) simple, bar-like, height more than twice width; cercus in posterior view (Fig. 9) pointed, nipple-like dorsally, first gradually broadened ventrally then rather abruptly curved laterally than medioventrally, generally gradually tapered, although medioventral portion almost parallel sided, dorsal and lateral portion bearing numerous setulae, most of medioventral portion bare except for 3 setulae at apex; cercus in lateral view (Fig. 10) very shallowly curved anteriorly, wider dorsally, ventral half slightly tapered, nearly parallel sided, apex not abruptly broadened and bearing 3 setulae, these setulae about same length as setulae on dorsal portions; surstylus a ventral extension from ventral margin of epandrium, becoming slightly wider apically and bifurcate, anterior process bearing several setulae anteriorly, posterior process bearing a single setula; aedeagus in ventral view (Fig. 11) elongate, narrowly ovate, slightly tapered apically, in lateral view (Fig. 13) elongate, nearly straight; phallapodeme in lateral view (Fig. 13) triangular, keel shorter than length, pointed; gonite in ventral view (Fig. 11) somewhat bar-like, lateral margins sinuous, wider basally, apex with a short, nipple-like process medially that bears a single, short setula, also a subapical, setula; gonite in lateral view (Fig. 13) wide basally, shallowly C-curved apically, tapered to apical point, with a subapical, nipple-like process, and apical portion narrow, elongate.

#### Type material.

The holotype male is labeled “USA. UTAH. Utah: Lake Shore (40°06.9'N, 111°41.8'W; 1370 m), 11 May 2007, D.&W.N.Mathis/HOLOTYPE ♂ *Allotrichoma bifurcatum* W. Mathis & T. Zatwarnicki USNM [red]/USNM ENT 00117956 [plastic bar code label].” The holotype is double mounted (minuten in a block of plastic elastomer), is in excellent condition, and is deposited in the USNM. Eighteen male paratypes bear the same locality label data as the holotype. Other paratypes are as follows: *UTAH. Salt Lake*: Butterfield Canyon (40°29.2'N, 112°08.2'W; 1890 m), 14 May-13 Aug 2007, 2008, D. and W. N. Mathis (8♂, 1♀; USNM). *Utah*: Thistle (40°0.4'N, 111°29.7'W; 1530 m), 11 May 2007, D. and W. N. Mathis (7♂, 2♀; USNM).

#### Type locality.

United States. Utah. Utah: Lake Shore (40°06.9'N, 111°41.8'W; 1370 m).

#### Other specimens examined from the New World.

CANADA. *BRITISH COLUMBIA*. Wasa Lake (49°47.6'N, 115°44.3'W), 17 Jul 1974, P. H. Arnaud, Jr. (1♂, 3♀; CAS).

UNITED STATES. *IDAHO. Boundary*: Dawson Lake (48°46.3'N, 116°14.3'W; 885 m), 3 Jun 2006, D. and W. N. Mathis, T. Zatwarnicki (2♂, 1♀; USNM); Solomon Lake (48°47.6'N, 116°06.1'W; 825 m), 3 Jun 2006, W. N. Mathis, T. Zatwarnicki (3♂; TZ, USNM). *Latah*: Bear Creek (46°37.8'N, 116°32'W), 28 Aug 1990, R. S. Zack (1♂; WSU); Big Meadow Creek (46°44'N, 116°45.5'W), 31 Jul 1979, R. S. Zack (5♂; WSU); Helmer (1.6 km W; Little Boulder Creek; 46°48'N, 116°29'W), 28 Aug 1990, R. S. Zack (1♂; WSU); Moscow (9.5 km N, 46°45'N, 117°W), 22 May 1971, W. J. Turner (1♂; WSU). *Nez Perce*: Lake Waha (46°12.4'N, 116°50.1'W), 9 Jun 1918, A. L. Melander (1♂; USNM).

*OREGON. Baker*: Goose Creek (35 km E Baker City; 44°49.2'N, 117°27.79'W; 825 m), 7 Jun 2006, D. and W. N. Mathis, T. Zatwarnicki (3♂; TZ, USNM); Lower Goose Creek (44°49.2'N, 117°27.79'W; 1220 m), 25 Jul 1976, E. J. Davis (1♂; WSU). *Deschutes*: Tumalo Reservoir (44°08.4'N, 121°24.9'W), 23 Jun 1954, G. F. Knowlton (1♂; KU).

*UTAH. Cache*: Hyde Park (41°47.9'N, 111°49.1'W), 4 Jul 1935, G. F. Knowlton (1♂; KU); Wellsville Canyon (41°36.2'N, 111°56.7'W), 28 Jun 1954, G. F. Knowlton (1♂; KU). *Carbon*: Deadman Canyon (16 km NE Price; 39°41.7'N, 110°44'W; 2055 m), 14 Aug 2008, D. and W. N. Mathis (1♂; USNM). *Salt Lake*: Butterfield Canyon (40°29.2'N, 112°08.2'W; 1890 m), 14 May–13 Aug 2007, 2008, D. and W. N. Mathis (7♂, 1♀; USNM); Draper (40°31.6'N, 111°55.1'W; Jordan River; 1320 m), 10 May 2007, D. and W. N. Mathis (2♂; USNM). *Utah*: Lake Shore (40°06.9'N, 111°41.8'W; 1370 m), 11 May 2007, D. and W. N. Mathis (19♂; USNM); Provo Canyon, Sundance (40°23.1'N, 111°34.9'W), 20 Jul 2001, I. Winkler (1♂; BYU); Thistle (40°0.4'N, 111°29.7'W; 1530 m), 11 May 2007, D. and W. N. Mathis (7♂, 2♀; USNM).

WASHINGTON. *Columbia*: Dayton, Curl Lake, Tucannon River (46°15.2'N, 117°40.3'W), 17 Jul 2009, D. Mathis (1♂, 1♀; USNM). *Okanogan*: Little Goose Lake (48°16.5'N, 119°31'W), 22 Jul 1983, R. D. Akre, R. S. Zack (1♂; WSU).

#### Distribution.

**Figure 14.**
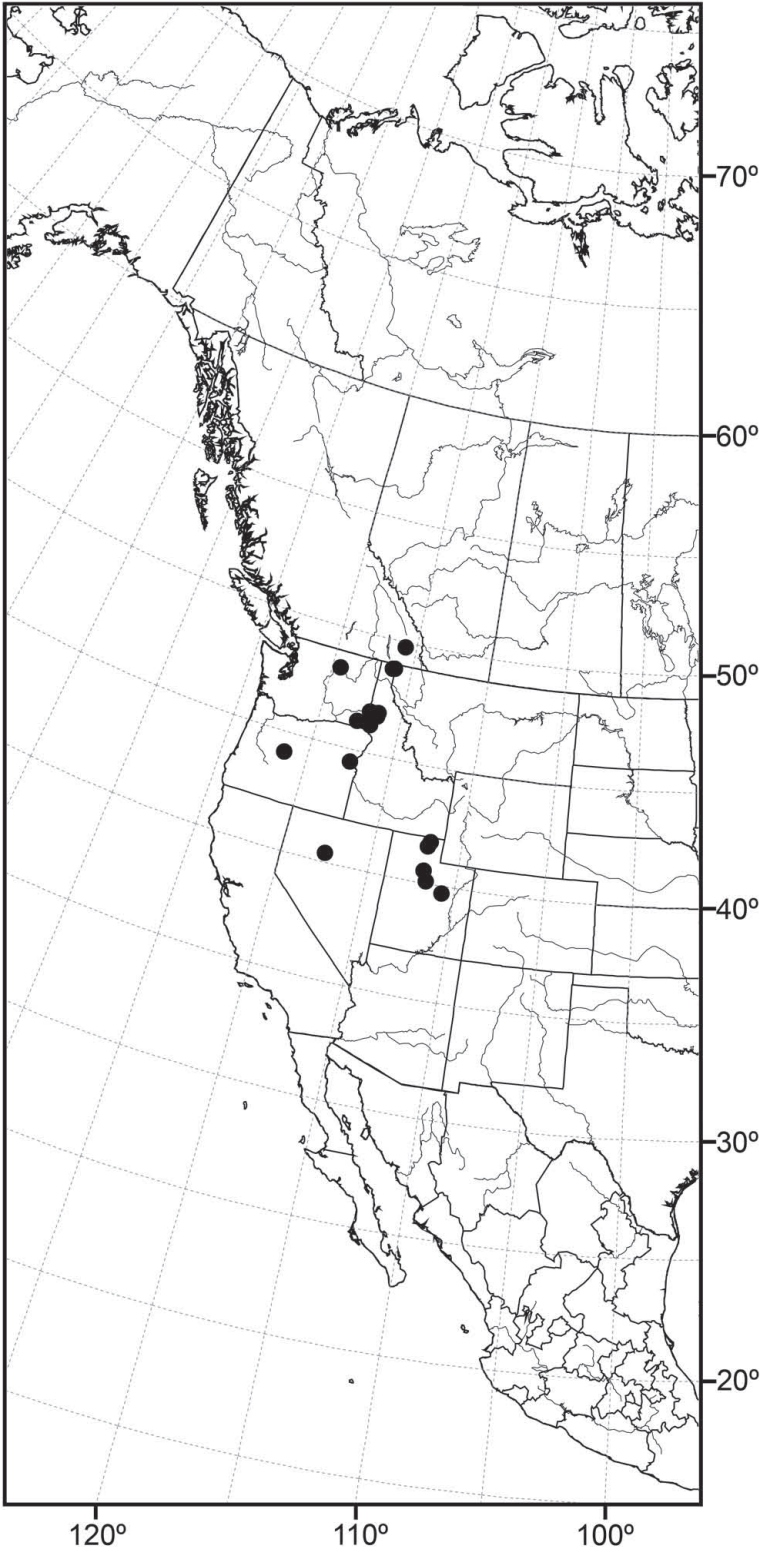
Distribution of *Allotrichoma bifurcatum* sp. n.

(Fig. 14) *Nearctic*: Canada (British Columbia), United States (Idaho, Oregon, Utah, Washington).

#### Etymology.

The species epithet, *bifurcatum*, is of Latin derivation and refers to the bifurcate apex of the surstylus of this species.

#### Remarks.

The distribution of this species is somewhat related to the Great Basin in western North America, where current climatic conditions are semiarid, although there is a northern extension into northern Idaho and British Columbia. Better sampling may reveal this species to be more widespread.

### Allotrichoma (Allotrichoma) deonieri

Mathis & Zatwarnicki

http://species-id.net/wiki/Allotrichoma_deonieri

Figs 15 20

Allotrichoma deonieri Mathis and Zatwarnicki 2010: 120 [United States. Virginia. Spotsylvania: Rappahannock River (38°18.8'N, 77°32.5'W); HT ♂, USNM].

#### Description.

This species is distinguished from congeners by the following combination of characters: Small shore flies, body length 1.20–1.95 mm. *Head*: Medial facial carina above facial prominence shallow; labella broad, fleshy, shorter than mediproboscis; clypeus microtomentose, usually gray.

*Thorax*: Presutural supra-alar seta present. Wing with costal vein ratio 0.31–0.39; M vein ratio 0.38–0.40.

**Figures 15–19.**
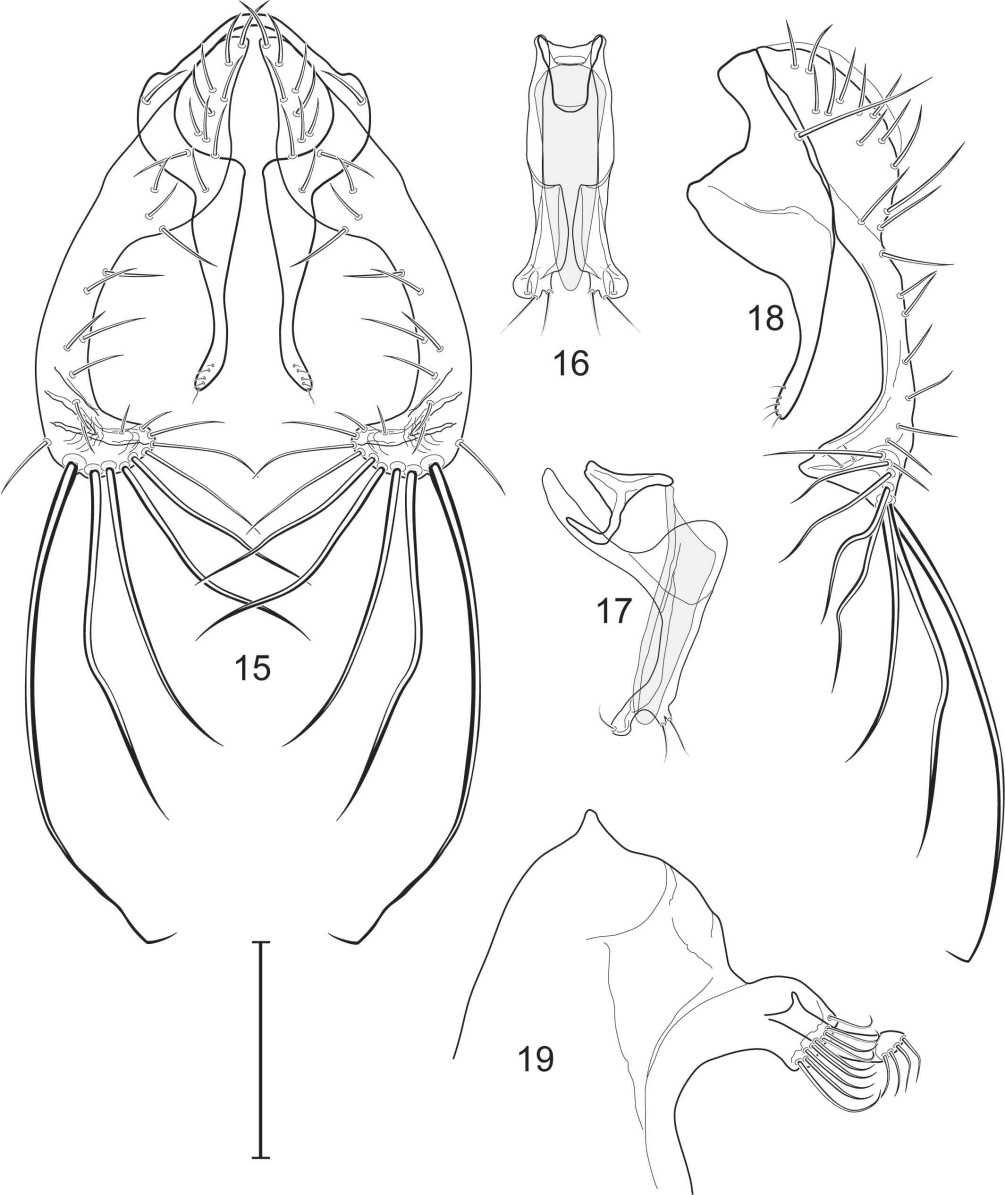
Illustration of *Allotrichoma deonieri* Mathis and Zatwarnicki (male) **15** epandrium, cerci, surstylus, posterior aspect **16** aedeagus, phallapodeme, gonite, hypandrium, ventral aspect **17** same, lateral aspect **18** epandrium, cerci, surstylus, lateral aspect **19** male 5th sternal flap and medial process, lateral aspect. Scale bar = 0.1 mm.

*Abdomen*: Male 5th sternal flap in lateral view (Fig. 19) moderately elongate, curved, truncate apex bearing numerous setulae; 5th medial process in lateral view (Fig. 19) elongate, bar-like, slightly curved, 4–5 setulae apically. Male terminalia (Figs 15–18): Epandrium in posterior view (Fig. 15) V-shaped with ventrolateral margin rounded and curved medially to base of surstyli; epandrium in lateral view (Fig. 18) somewhat narrowly rectangular on dorsal half than abruptly projected and angled anteriorly before taper posteroventral to base of surstyli; cerci in posterior view (Fig. 15) approximate dorsally then extended laterally to form an inverted V, each cercus robustly developed, medial margin distinctly sinuous, lateral margin shallowly and mostly evenly curved, bearing setae throughout length, especially medially and with very long setulae apically at expanded apex; cercus in lateral view (Fig. 18) lunate on dorsal third, then narrowed, linear before expanded, truncate apex; surstyli (ventral extensions of epandrium) in posterior view narrow, broader at base, tapered to apex, apical 1/3 shallowly recurved and very slightly spatulate, bearing several short setulae; surstylus in lateral view (Fig. 18) tapered on basal third, thereafter ventrally parallel sided and shallowly curved, apical fourth bearing several short setulae; aedeagus in ventral view (Fig. 16) elongate, narrowly ovate, bullet-like; very slightly tapered apically, in lateral view (Fig. 17) elongate, nearly parallel sided, apex bluntly rounded; phallapodeme in lateral view (Fig. 17) triangular, keel moderately tapered to form a triangular; gonite in ventral view (Fig. 16) bar-like, mostly parallel sided except for short, lateral expansion at apex; gonite in lateral view (Fig. 17) generally elongate, moderately wider basally, thereafter tapered gradually toward apex, apex produced as a lateral and ventral process, lateral process bearing 2 setulae, apical, process longer, bearing 1 setula.

#### Type material.

The holotype male *Allotrichoma deonieri* is labeled “**USA. V[IRGINI]A.** Spotsylvania: Rappahannock River[,] 38°18.8'N, 77°32.5'W), 14 Aug 2006, D. & Wayne N. Mathis/USNM ENT 00199488 [plastic bar code label]/HOLOTYPE ♂ *Allotrichoma deonieri* W. Mathis & T. Zatwarnicki USNM [red].” The holotype is double mounted (minuten in a block of plastic), is in excellent condition, and is deposited in the USNM. Thirteen paratypes (10♂, 3♀; USNM) bear the same label data as the holotype. Other paratypes are as follows: *VIRGINIA. Fairfax*: Great Falls (Clay Pond; 39°00.1'N, 77°15.4'W), 13 Jun-17 Aug 2006, 2007, D. and W. N. Mathis (3♂; USNM); Great Falls (Patowmack Canal; 39°00.1'N, 77°15.2'W), 25 Jul-29 Aug 2006, 2007, 2008, D. and W. N. Mathis (8♂; USNM); Turkey Run (38°57.8'N, 77°09.4'W), 10 Jul 2008, W. N. Mathis (1♂; USNM); Turkey Run (mouth; 38°57.9'N, 77°09.4'W), 8 Aug-17 Sep 2006, 2007, 2008, W. N. Mathis (31♂, 2♀; USNM). *Rappahannock*: Hazel River (NW Culpeper; 38°33.8'N, 78°11.6'W, 171 m), 28 Jun-24 Jul 2008, W. N. Mathis and T. Zatwarnicki (3♂; USNM). *Spotsylvania*: Rappahannock River (38°18.8'N, 77°32.5'W), 3 Jul 2007, D. and W. N. Mathis (9♂; USNM). *Stafford*: Aquia Harbour, Aquia Creek (38°27.8'N, 77°23.1'W), 2 Sep 2006, D. and W. N. Mathis (1♂; USNM); Aquia Harbour, Lions Park (38°27'N, 77°23.3'W), 10 Apr-11 Aug 2006, 2009, W. N. Mathis (2♂; USNM); Falmouth (38°19.2'N, 77°28.1'W; Rappahannock River; 9 m), 30 Jun 2007, D. and W. N. Mathis (3♂; USNM). *York*: Maury Lake (ca. James River; 37°02.5'N, 76°29.2'W), 19 Aug 2006, D. and W. N. Mathis (1♂; USNM).

#### Type locality.

United States. Virginia. *Spotsylvania*: Rappahannock River (38°18.8'N, 77°32.5'W).

#### Other specimens examined from the New World.

UNITED STATES. *FLORIDA. Walton*: Morrison Spring (30°38.7'N, 85°53.7'W), 31 Jul 1962, D. L. Deonier (1♂; USNM).

*LOUISIANA. East Carroll*: Lake Providence (32°48.3'N, 91°10.2'W), 14 Jul 1953, W. W. Wirth (1♂, 2♀; USNM).

*MARYLAND. Garrett*: Broadford Lake (39°24.7'N, 79°22.4'W; 750 m), 20 Jun 2007, D. and W. N. Mathis (1♂; USNM).

*MISSISSIPPI. Washington*: Leroy Percy State Park (W Hollandale; 33°09.8'N, 90°56.2'W; 63 m), 9 Jun 2004, W. N. Mathis (6♂, 3♀; USNM).

*TENNESSEE. Obion*: Reelfoot State Park, Reelfoot Lake (36°21.1'N, 89°25.5'W), 16 Aug 1962, D. L. Deonier (3♂, 7♀; USNM). *Shelby*: Meeman Shelby State Park (Piersol Lake; 35°20.4'N, 90°02.1'W; 98 m), 10 Jun 2004, W. N. Mathis (4♂, 4♀; USNM).

*WEST VIRGINIA. Mercer*: Ceres (Kee Reservoir; 37°18.4'N, 81°10.4'W; 757 m), 24 Sep 2007, D. and W. N. Mathis (1♂; USNM).

#### Distribution.

**Figure 20.**
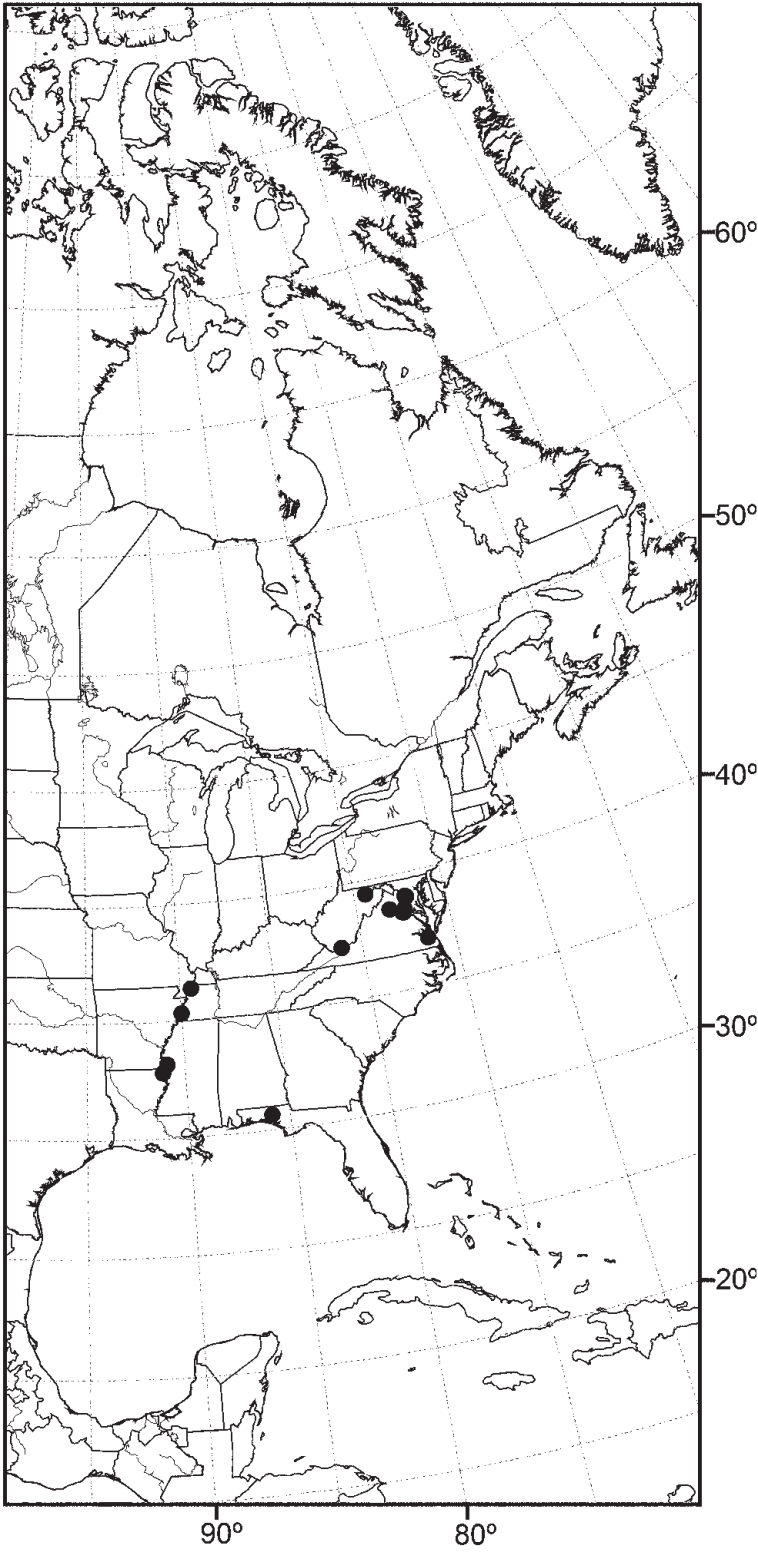
Distribution of *Allotrichoma deonieri* Mathis and Zatwarnicki.

(Fig. 20) *Nearctic*: United States (Florida, Louisiana, Maryland, Mississippi, Tennessee, Virginia, West Virginia).

#### Etymology.

The species epithet, *deonieri*, is a genitive patronym to honor one of the collectors of this species, D. L. Deonier, a long-time student of shore flies. Dick is not only our friend and colleague but an excellent field biologist. His collecting efforts have greatly assisted our research on shore flies.

#### Remarks.

Although most records of this species are from the drainage of the Mississippi River, including nearby tributaries, the records from Maryland and Virginia represent a completely different drainage system.

### Allotrichoma (Allotrichoma) dynatum sp. n.

urn:lsid:zoobank.org:act:5D7781D0-CEC5-4870-97BB-F5D6466F155B

http://species-id.net/wiki/Allotrichoma_dynatum

Figs 21 26

Allotrichoma laterale of authors, not Loew 1860 [misidentification]. Becker 1896: 121 [generic combination]. Williston 1897: 4 [compared with *Allotrichoma abdominale* Williston]. Cresson 1942: 108 [list, west coast of United States]. Wirth and Stone 1956: 467 [key]. Wirth 1965: 736 [Nearctic catalog, in part]. Cole 1969: 397 [list]. Mathis and Zatwarnicki 1995: 151–152 [world catalog, in part].

#### Description.

This species is distinguished from congeners by the following combination of characters: Small to moderately small shore flies, body length 1.40–2.00 mm. *Head*: Medial facial carina above facial prominence shallow; labella broad, fleshy, shorter than mediproboscis; clypeus microtomentose, usually gray.

*Thorax*: Presutural supra-alar seta present. Wing with costal vein ratio 0.35–0.36; M vein ratio 0.47–0.52.

**Figures 21–25.**
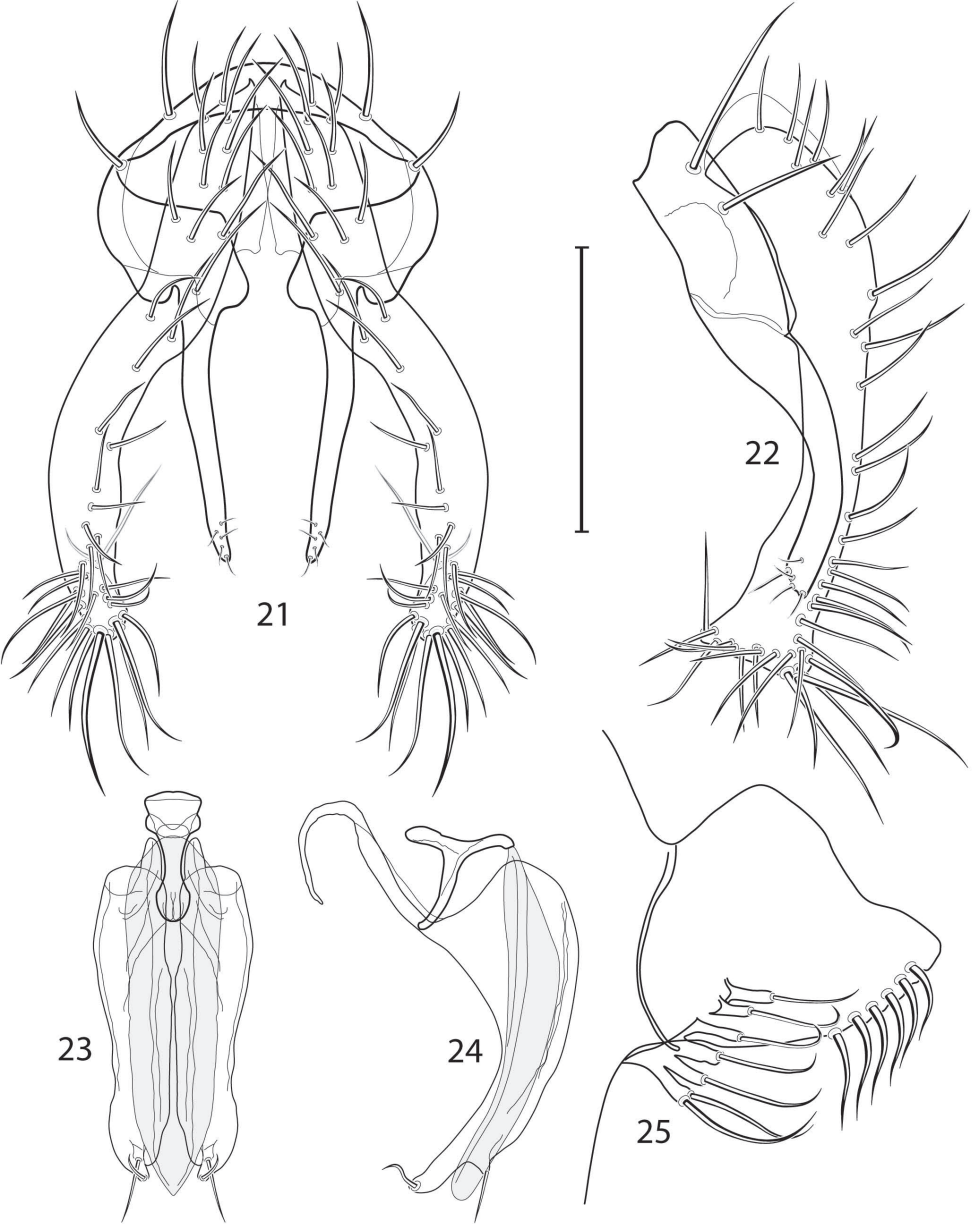
Illustration of *Allotrichoma dynatum* sp. n. (male) **21** epandrium, cerci, surstylus, posterior aspect **22** same, lateral aspect **23** aedeagus, phallapodeme, gonite, hypandrium, ventral aspect **24** same, lateral aspect **25** male 5th sternal flap and medial process, lateral aspect. Scale bar = 0.1 mm.

*Abdomen*: Male 5th sternal flap in lateral view (Fig. 25) relatively shallow but long, bearing 6–8, tuberculate setae; 5th medial process in lateral view (Fig. 25) robust, greatly enlarged basally, with a posterior projection, broadly pointed apically, bear row of setulae anteroapically. Male terminalia (Figs 21–24): Epandrium in posterior view (Fig. 21) ovately rounded on dorsal half, wider than high; epandrium in lateral view (Fig. 22) with anterior and posterior margins of dorsal half nearly straight and parallel sided; cercus robust, thick, in posterior view (Fig. 21) pointed dorsally, gradually broadened ventrally then rather abruptly curved laterally and ventrally as a slightly tapered, elongate process that bears numerous long setulae apically and along posterior margin; cercus in lateral view (Fig. 22) slightly curved, wider dorsally, ventral half nearly parallel sided, apex broadened and bearing setulae; surstyli in posterior view as parallel, narrow, elongate processes that bear setulae on apical portion, apex narrowly rounded; surstylus in lateral view shallowly curved, basal half slightly tapered, apical half almost parallel sided, apical portion bearing short setulae, apex tapered to narrow point; aedeagus in ventral view (Fig. 23) elongate, moderately narrowly ovate, tapered apically, in lateral view (Fig. 24) narrowly elongate, somewhat lunate; phallapodeme in lateral view (Fig. 24) very narrowly triangular, digitiform keel shorter than length, pointed; gonite in ventral view (Fig. 23) bar-like, very slightly wider basally, apex with short, lateral process, apex bearing setula, in lateral view (Fig. 24) wide basally, gently curved, tapered to apical point, with a subapical, short process that bears a setula.

#### Type material.

The holotype male is labeled “OREGON BentonCo Finley WildlRefg 18 June 1976 Wayne N. Mathis/HOLOTYPE ♂ *Allotrichoma dynatum* W. Mathis & T. Zatwarnicki USNM [red]/USNM ENT 00117957 [plastic bar code label].” The holotype is double mounted (minuten in a block of plastic), is in excellent condition, and is deposited in the USNM. Fifty paratypes (27♂, 23♀; USNM) bear the same locality data as the holotype but with collection dates from 3 Jul 1972 to 18 Jun 1976.

#### Type locality.

United States. Oregon. Benton: Finley National Wildlife Refuge (44°24.6'N, 123°19.5'W).

#### Other specimens examined from the New World.

CANADA. *BRITISH COLUMBIA*. Revelstoke, Murphys Ranch (50°58'N, 118°48'W), 1 Jul 1968, W. W. Wirth (1♂; USNM). Savona (50°45.2’N, 120°50.6’W), 19 Jul 1988, R. Danielsson (1♂; ZIL).

UNITED STATES. *OREGON. Benton*: Cary's Grove (44°22.6'N, 123°36.1'W), 2 Sep 1974, W. N. Mathis (2♂, 1♀; USNM); Corvallis (44°33.9'N, 123°15.7'W), 15 Jul 1972, W. N. Mathis (1♂; USNM); McDonald Forest, Oak Creek (44°33.3'N, 123°16.7'W), 30 Jun 1971, G. C. Steyskal (1♂; USNM); Rock Creek (6.4 km SW Philomath; 44°30.1'N, 123°26.2'W), 29 May 1972, W. N. Mathis (1♂; USNM). *Douglas*: North Umpqua River (42°43.2'N, 122°60'W; 250 m), 31 Jul 2005, D. and W. N. Mathis (1♂; USNM). *Josephine*: Grants Pass (42°26.3'N, 123°19.7'W), 4 Aug 1952, H. A. Scullen (1♂; OSU). *Linn*: Waterloo (44°29.6'N, 122°49.5'W), 24 Jul 1974, W. N. Mathis (3♂; USNM).

*WASHINGTON. Clallam*: Sequim (48°04.8'N, 123°06.1'W), 2 Aug 1951 (1♂; USNM). *Pacific*: Ocean Park (46°29.5'N, 124°03'W), 22 Aug 1957, M. T. James (1♂; USNM).

#### Distribution.

**Figure 26.**
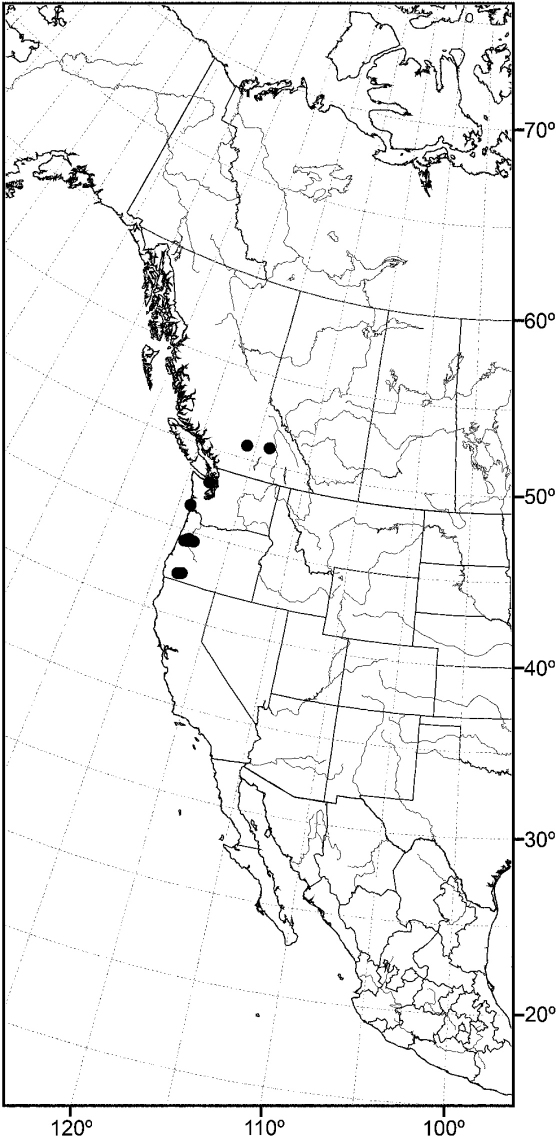
Distribution of *Allotrichoma dynatum* sp. n.

(Fig. 26) *Nearctic*: Canada (British Columbia), United States (Oregon, Washington).

#### Etymology.

The species epithet, *dynatum*, is derived from the Greek work *dynamis*, meaning strong or powerful, alluding to the well-developed cerci of this species.

#### Remarks.

In the Nearctic Region, this is a western species that has been collected west of the Sierra/Cascade cordillera in the states of Oregon and Washington and in the province of British Columbia.

### Allotrichoma (Allotrichoma) lacteum

Cresson

http://species-id.net/wiki/Allotrichoma_lacteum

Figs 27 32

Allotrichoma lacteum Cresson 1926: 252; 1948: 260 [synonymy with *Allotrichoma simplex*]. Wirth 1965: 736 [Nearctic catalog]. Cole 1969: 397 [list]. Mathis and Zatwarnicki 1995: 151 [world catalog].

#### Description.

This species is distinguished from congeners by the following combination of characters: Small to moderately small shore flies, body length 1.50–2.25 mm. *Head*: Medial facial carina above facial prominence shallow; labella broad, fleshy, shorter than mediproboscis; clypeus microtomentose, usually gray.

*Thorax*: Presutural supra-alar seta present. Wing with costal vein ratio 0.28–0.32; M vein ratio 0.43–0.47.

**Figures 27–31.**
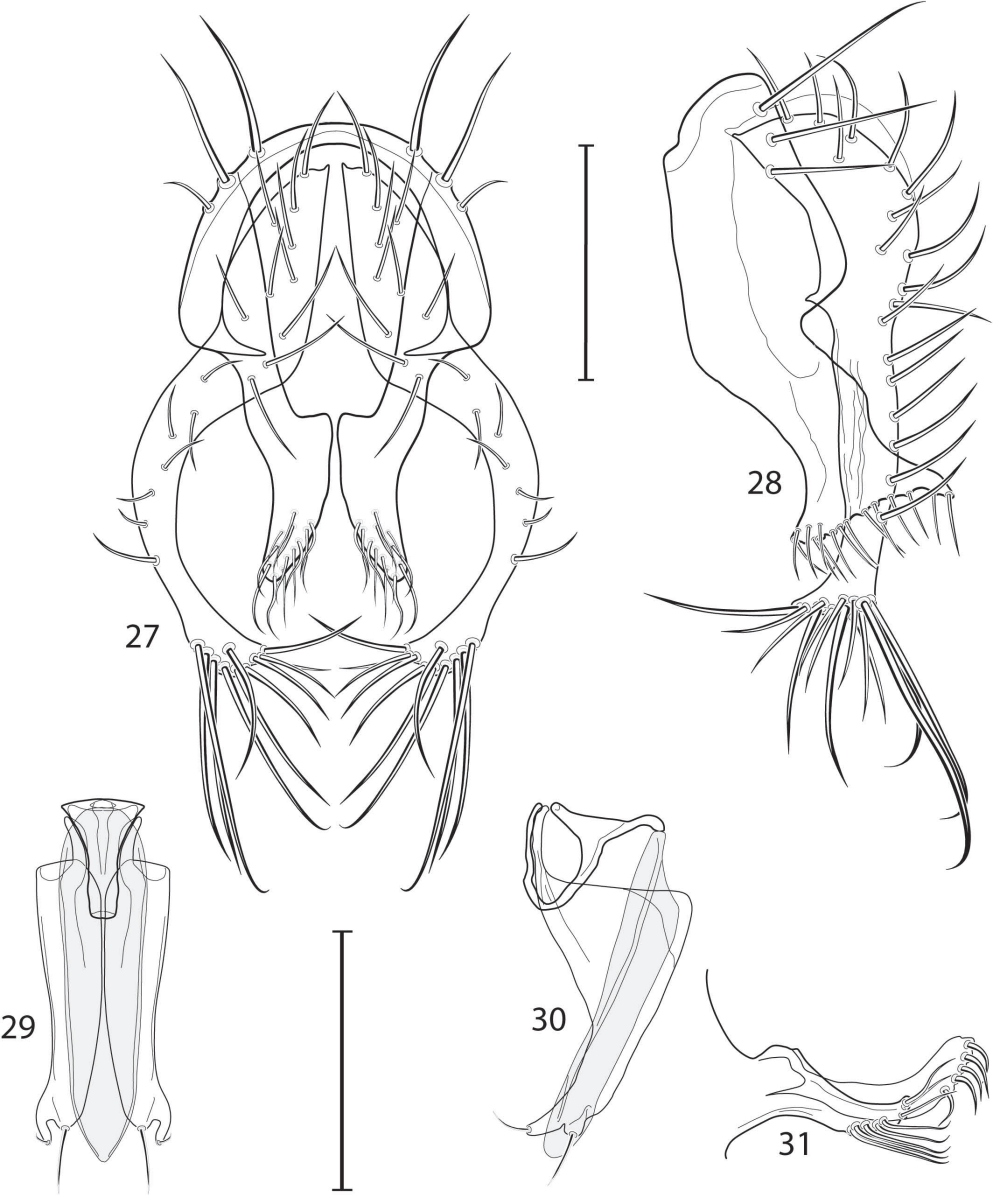
Illustration of *Allotrichoma lacteum* Cresson (male) **27** epandrium, cerci, surstylus, posterior aspect **28** same, lateral aspect **29** aedeagus, phallapodeme, gonite, hypandrium, ventral aspect **30** same, lateral aspect **31** male 5th sternal flap and medial process, lateral aspect. Scale bar = 0.1 mm.

*Abdomen*: Male 5th sternal flap in lateral view (Fig. 31) elongate, digitiform, bear several setulae apically and subapically; 5th medial process in lateral view (Fig. 31) elongate, bar-like, bearing 5 apical setulae. Male terminalia (Figs 27–30): Epandrium in posterior view (Fig. 27) like an inverted U with medial flanges, flanges becoming wider ventrally; epandrium in lateral view (Fig. 28) simple, bar-like, height more than twice width; surstylus robust, greatly widened ventrally, posteroventral and anteroventral corners narrowly angulate, ventral margin bearing numerous short setulae; cercus in posterior view (Fig. 27) pointed dorsally, gradually broadened ventrally then abruptly curved laterally and ventrally as a slightly tapered, elongate process that bears numerous, very long setulae apically; cercus in lateral view (Fig. 28) essentially straight, wider dorsally, ventral half slightly tapered, nearly parallel sided, apex abruptly broadened and bearing numerous, very long setulae; aedeagus in ventral view (Fig. 29) elongate, narrowly ovate, slightly tapered apically, in lateral view (Fig. 30) elongate, somewhat lunate; phallapodeme in lateral view (Fig. 30) triangular, keel shorter than length, pointed; gonite in ventral view (Fig. 29) somewhat bar-like, slightly wider basally, apex with short, lateral process, apex of process bearing a setula, with a subapical, distinct, angulate notch and bearing a subapical setula; gonite in lateral view (Fig. 30) wide basally, gently curved apically, tapered to apical point.

#### Type material.

The holotype male is labeled “Bill W[illia]ms. For[est,] Ariz[ona]. Aug. F. H. Snow/♂/TYPE Allotrichoma LACTEUM E. T. Cresson, Jr. [maroon; species name handwritten].” The holotype is double mounted, is in good condition (abdomen removed and apparently lost; there is an empty attached microvial with glycerin), and is deposited in the Snow Entomological Collections (KU). Cresson also listed two male and one female paratopotypes, and we have examined the female allotype, which is a topotypical paratype (KU), and a male, topotypical male paratype (ANSP).

#### Type locality.

United States. Arizona. Coconino: Bill Williams Forest (35°12'N, 112°12'W).

#### Other specimens examined from the New World.

UNITED STATES. *ARIZONA. Apache*: Adamana (34°58.6'N, 109°49.3'W), 12 Jun 1950, A. H. Sturtevant (1♂; USNM). *Coconino*: Bill Williams Forest (35°12'N, 112°12'W), F. E. Snow (1♂; ANSP). *La Paz*: Parker (34°09'N, 114°17'W), Jun 1943, W. C. Reeves (1♂; USNM). *Navajo*: Joseph City (35°57.3'N, 110°20'W), 12 Jun 1950, A. H. Sturtevant (1♂; USNM); Kayenta (36°44.4'N, 110°14.4'W), 14 Apr 2003, W. N. Mathis, T. Zatwarnicki (14♂, 5♀; USNM). *Yuma*: Yuma (32°43.5'N, 114°37.5'W), 3 Jun-6 Sep 1952, 1953, D. M. Tuttle, Haga (8♂, 14♀; UAR, USNM).

*CALIFORNIA. Imperial*: Calexico (32°40.7'N, 115°29.9'W), 31 Jul 1958, E. I. Schlinger (3♂, 1♀; UCR); Hot Mineral (33°25.5'N, 115°41.1'W), 25–30 Apr 1952, 1953, J. N. Belkin, W. W. Wirth (12♂, 9♀; LACM, USNM); Palo Verde (33°26'N, 114°44'W), 8 Apr 1949, W. W. Wirth (3♂, 3♀; USNM). *Riverside*: Thermal (33°38.4'N, 116°08.4'W), 24 Aug 1965, S. Frommer, D. Gerling (1♂; UCR). *San Diego*: Borrego (33°13.2'N, 116°20.1'W), 2 May 1945, A. L. Melander (1♂; USNM); Borrego Sink (11 km SE Borrego Springs; 33°12.9'N, 116°13.5'W), 14 Sep 1969, J. B. Heppner (1♂, 2♀; FSCA); Palm Spring (33°55.2'N, 116°13.1'W), 21 May 1953, A. L. Melander (1♂; USNM).

*COLORADO. Archuleta*: Pagosa Springs (37°15.8'N, 107°00.7'W; hot springs), 27 May 1969, W. W. Wirth (1♂; USNM). *Moffat*: Hells Canyon (9.7 km W; 40°28.3'N, 108°53.7'W), 21 Jun 1946, M. T. James (3♂; UCO). *Uintah*: Lodore (40°38'N, 108°57.4'W), 23 Jun 1948, M. T. James (1♂; UCO).

*IDAHO. Benewah*: Chatcolet (47°22.3'N, 116°54.8'W), Aug 1915, A. L. Melander (3♀; USNM). *Bonner*: Priest Lake (48°34.5‘N, 116°57.4‘W), 1 Aug 1916, A. L. Melander (1♂, 2♀; USNM). *Cassia*: Declo (42°31.1'N, 113°37.7'W; on *Beta vulgaris*), 3 Aug 1931, H. Waters (1♀; USNM).

*MONTANA. Glacier*: Glacier National Park, St. Mary Lake (48°47.4'N, 113°25.2'W), 2 Aug 1935, A. L. Melander (1♂; USNM). *Lake*: Ninepipe National Wildlife Refuge (47°26.5'N, 114°07.3'W), 14 Sep 1983. R. S. Zack (1♂; WSU). *Lincoln*: Troy (48°27.8'N, 115°53.4'W), 29 Jun 1954, A. H. Sturtevant (1♀; USNM). *Sheridan*: Medicine Lake (48°30.1'N, 104°30.3'W), 9 Jun 1969, W. W. Wirth (1♂; USNM).

*NEBRASKA. Sheridan*: Hay Springs (42°41'N, 102°41.3'W), 17 Sep 1952, A. H. Sturtevant (1♂; USNM).

*NEVADA. Clark*: Warm Springs, N. Glendale (36°42.5'N, 114°42.9'W), 18 May 1987, D. E. Hardy (1♂; BYU).

*NEW MEXICO. Cibola*: Bluewater Lake State Park (35°18.1'N, 108°06.6'W; 2220 m), 9 Aug 2007, D. and W. N. Mathis (5♂, 2♀; USNM). *Socorro*: Socorro (33°55.9'N, 106°57'W), 1916, S. W. Williston (1♂; ANSP). *Valencia*: Rio Puerco (34°47.8'N, 106°59.5'W; 1575 m), 9 Aug 2007, D. and W. N. Mathis (2♂; USNM).

*TEXAS. Brewster*: Big Bend National Park, Boquillas Canyon (29°12.7'N, 102°53.2'W), 20 Jun 1953, W. W. Wirth (1♂, 1♀; USNM). *Randall*: Palo Duro State Park (34°56'N, 101°39.8'W), 21 Sep 1954, A. H. Sturtevant (1♀; USNM). *Jefferson*: Fort Davis, near Limpia Creek (30°59'N, 103°88'W), 15 Jul 1963, A. B. Gurney (1♀; USNM).

*UTAH. Carbon*: Deadman Canyon (16 km NE Price; 39°41.7'N, 110°44'W; 2055 m), 19 Aug 2009, W. N. Mathis (1♂; USNM). *Emery*: Green River (Green River; 38°59.6'N, 110°08.5'W; 1240 m), 31 Jul 2007, D. and W. N. Mathis (1♂; USNM); Green River (3.3 km N, 39°01.7'N, 110°09.7'W; 1253 m), 30 Jul-16 Aug 2007, 2008, D. and W. N. Mathis (16♂; USNM); San Rafael River (22.5 km SW Green River; 38°55.7'N, 110°24.5'W; 1270 m), 31 Jul 2007, D. and W. N. Mathis (13♂; USNM). *Garfield*: Boulder Mail Trail/Upper Calf Creek Falls (37°52.3'N, 111°28.3'W), 12 Jul 2000, M. Moody, C. R. Nelson (1♂; BYU); Collet Top Junction, Smoky Mountain Road (37°28.1'N, 111°33'W), 20 Jun 2000, E. C. Green, W. N. Mendel (1♂; BYU); Willow Tank-Hurricane Wash (37°23.2'N, 111°08'W; stock tank), 22 May 2001, D. and W. N. Mathis (1♂; USNM). *Grand*: Crystal Geyser (14.5 km SE Green River; 38°56.3'N, 110°08.1'W), 15 Aug 2008, D. and W. N. Mathis (7♂; USNM); Swasey Beach (15.3 km N Green River; 39°07'N, 110°06.6'W; Green River; 1255 m), 29 May-15 Aug 2007, 2008, D. and W. N. Mathis (21♂; USNM); Thompson Spring (8.9 km N Thompson Springs; 39°02.3'N, 109°43.4'W; 1740 m), 1-20 Aug 2007, 2008, D. and W. N. Mathis, T. Zatwarnicki (26♂, 2♀; USNM). *Kane*: Drip Tank Canyon (37°19.4'N, 111°31.8'W), 15 May-6 Jul 2000, 2001, D. and W. N. Mathis, C. R. Nelson (8♂; BYU, USNM); East Fork of Virgin River (26.6 km N Kanab; 37°12.2'N, 112°41.4'W) 18 May 2001, D. and W. N. Mathis (6♂; USNM); Last Chance Creek Junction, Smoky Mountain Road (37°20.8'N, 111°31.6'W; 1920 m), 19 Jun 2000, E. C. Green, W. N. Mendel (2♂; BYU); Sheep Creek (37°29.7'N, 112°04'W), 17 May 2001, D. and W. N. Mathis (2♂; USNM); White House Spring (71 km E Kanab; 37°04.8'N, 111°53.4'W; 1250 m) 19 May 2001, D. and W. N. Mathis (2♂, 1♀; USNM); Willis Creek (37°29'N, 112°05.8'W), 17 May 2001, D. and W. N. Mathis (8♂, 1♀; USNM). *Salt Lake*: Murray (40°40'N, 111°53.2'W; on celery), 13 Sep 1950, G. F. Knowlton (1♂; USNM). *Utah*: Goshen Hot Springs (39°57.8'N, 111°51.2'W), 16 Apr 2003, W. N. Mathis, T. Zatwarnicki (5♂, 4♀; USNM). *Uintah*: Vernal Mountains (40°29.5'N, 109°27'W), 17 Jul 1952, G. E. Bohart, G. F. Knowlton (1♂; USNM).

*WASHINGTON. Grant*: Columbia National Wildlife Refuge (46°51.1'N, 119°32.2'W), 13 May 1959, H. F. Harwood (1♂; USNM). *Pierce*: Mt. Rainer National Park, Yakima Park (46°55'N, 121°38'W), 14 Aug 1940, A. L. Melander (1♀; USNM).

*WYOMING. Hot Springs*: Thermopolis (43°38.8'N, 108°12.6'W), 30 Aug 1940, A. L. Melander (1♂, 5♀; USNM).

#### Distribution.

**Figure 32.**
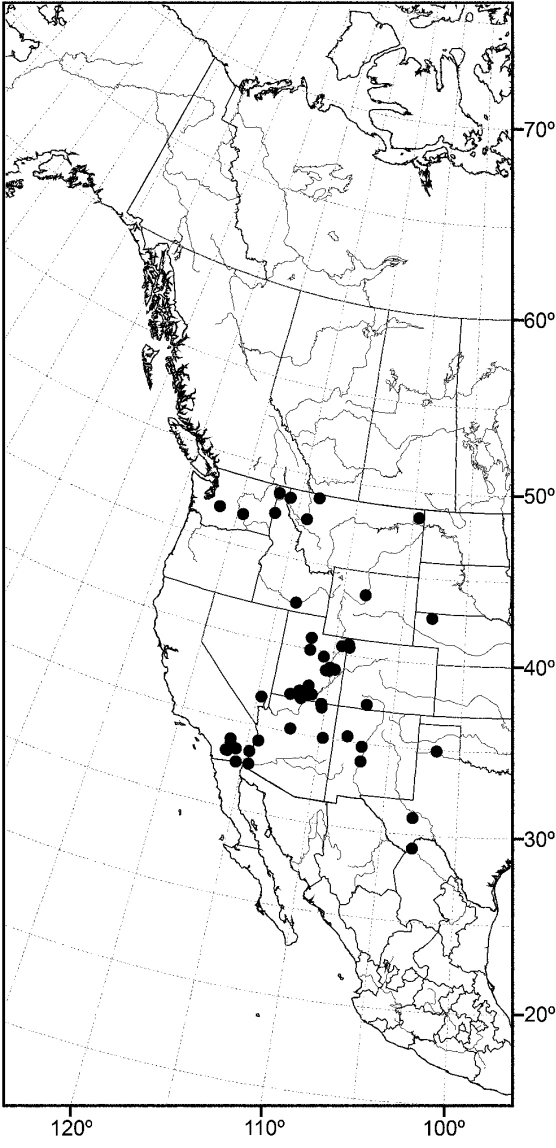
Distribution of *Allotrichoma lacteum* Cresson.

(Fig. 32) *Nearctic*: United States (Arizona, California, Colorado, Idaho, Montana, Nebraska, Nevada, New Mexico, Texas, Utah, Washington, Wyoming).

#### Remarks.

Like *Allotrichoma bezzii*, this species occurs in the western United States, although not as far north as Alaska.

Although similar to *Allotrichoma sabroskyi*, the greatly widened surstylus that bears numerous short setulae easily distinguishes this species from it and all other congeners.

### Allotrichoma (Allotrichoma) lasiocercum

Cresson

http://species-id.net/wiki/Allotrichoma_lasiocercum

Figs 33 38

Allotrichoma lasiocercum Cresson 1926: 251; 1948: 260 [synonymy with *Allotrichoma simplex*]. Wirth 1965: 736 [Nearctic catalog]. Cole 1969: 397 [list]. Mathis and Zatwarnicki 1995: 151 [world catalog].

#### Description.

This species is distinguished from congeners by the following combination of characters: Small to moderately small shore flies, body length 1.55–2.15 mm. *Head*: Medial facial carina above facial prominence shallow; labella broad, fleshy, shorter than mediproboscis; clypeus microtomentose, usually gray.

*Thorax*: Presutural supra-alar seta present. Pleural area lacking stripes. Wing with costal vein ratio 0.36–0.38; M vein ratio 0.37–0.40. Legs blackish brown to black; midtibia bearing numerous, erect setae along dorsal surface.

**Figures 33–37.**
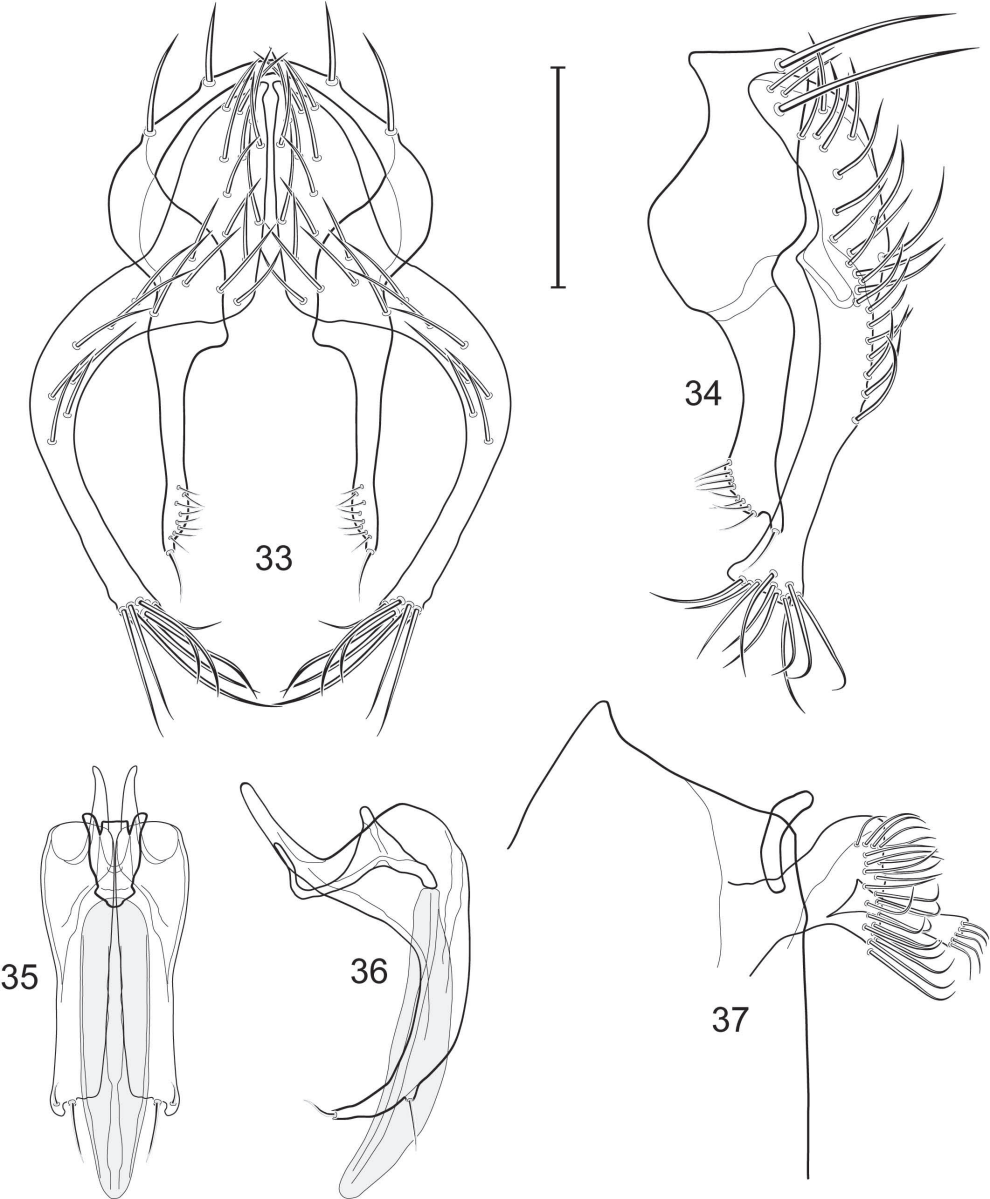
Illustration of *Allotrichoma lasiocercum* Cresson (male) **33** epandrium, cerci, surstylus, posterior aspect **34** same, lateral aspect **35** aedeagus, phallapodeme, gonite, hypandrium, ventral aspect **36** same, lateral aspect **37** male 5th sternal flap and medial process, lateral aspect. Scale bar = 0.1 mm.

*Abdomen*: Male 5th sternal flap in lateral view (Fig. 37) pedunculate, relatively narrow at base, becoming wider apically, bearing numerous setulae along truncate apex; 5th medial process in lateral view (Fig. 37) elongate, bar-like, bearing 4–5 apical setulae. Male terminalia (Figs 33–36): Epandrium in posterior view (Fig. 33) ovately rounded on dorsal half; epandrium in lateral view (Fig. 34) with anterior and posterior margins sinuous, relatively wide dorsally; cercus in posterior view (Fig. 33) broadly pointed dorsally, gradually broadened ventrally then abruptly curved or arched laterally and ventrally as a slightly tapered, elongate, arched process that bears numerous moderately long setulae apically; cercus in lateral view (Fig. 34) shallowly arched, wider dorsally, ventral half nearly parallel sided, apex broadened and bearing moderately long setulae; surstyli (ventral extensions of epandrium) in posterior view as parallel, narrow processes that bear setulae on apical portion; surstylus in lateral view narrowed and nearly parallel sided, apical portion bearing setulae anteriorly and apically, with a short, apical process that bears a longer, apical setula; aedeagus in ventral view (Fig. 35) elongate, narrowly ovate, tapered apically, in lateral view (Fig. 36) elongate, somewhat lunate; phallapodeme in lateral view (Fig. 36) triangular, keel shorter than length, pointed; gonite in ventral view (Fig. 35) bar-like, slightly wider basally, apex with short, lateral process, apex bearing setula, in lateral view (Fig. 36) wide basally, gently but distinctly arched, tapered to apical point.

#### Type material.

The holotype male is labeled “Berkeley Hills[,] Alameda Co.[,] IV,11,'08 [11 Apr 1908], Cal[ifornia]/♂/241/TYPE Allotrichoma, LASIOCERCUM, E.T. Cresson, Jr [red; species and generic names handwritten]/Loaned by A.N.S.P.” The holotype male is double mounted (minuten in a small rectangular card), is in good condition (abdomen removed, dissected, stored in glycerin in an attached microvial), and is deposited in the ANSP (6319). Cresson also listed two female paratopotypes (ANSP).

#### Type locality.

United States. California. Alameda: Berkeley Hills (37°53'N, 122°14'W).

#### Other specimens examined from the New World.

UNITED STATES. *CALIFORNIA. Inyo*: Tecopa Hot Spring (35°52.7'N, 116°13.9'W), 12 Apr 2003, W. N. Mathis, T. Zatwarnicki (1♀; USNM). *Marin*: Novato, San Jose Creek (38°06.5'N, 122°34.2'W; 45 m), 10 Jul 1971, M. M. and P. H. Arnaud, Jr. (1♂; CAS). *Mendocino*: Russian River near Hopland (38°58.4'N, 123°07'W), 4 May 1968, W. J. Turner (1♂; WSU). *Mono*: Mammoth lakes (37°36.3'N, 119°0.7'W), 29 Jul 1940, L. C. Kulters (1♂; KU); Mono Lake (38°01'N, 119°0.1'W), 16 Aug 1958 (1♂; USNM). *Riverside*: Martinez (33°33.8'N, 116°09.2'W), 11 Jul 1917, J. M. Aldrich (1♂; USNM). *San Bernardino*: Saratoga Spring (35°40.9'N, 116°25.3'W; 100 m), 31 Mar 2005, D. and W. N. Mathis (2♂, 7♀; USNM). *Santa Clara*: San Jose (37°20.4'N, 121°53.7'W), 8 Sep 1949, L. W. Quate (1♂, 1♀; USNM); San Jose, Guadalupe Creek (37°14.8'N, 121°52.3'W), 14 Jun 1966, R. E. Orth (1♂; UCR). *San Diego*: Carlsbad (33°09.5'N, 117°21'W), 1 Jun 1954, J. C. Hall (1♂; USNM).

#### Distribution.

**Figure 38.**
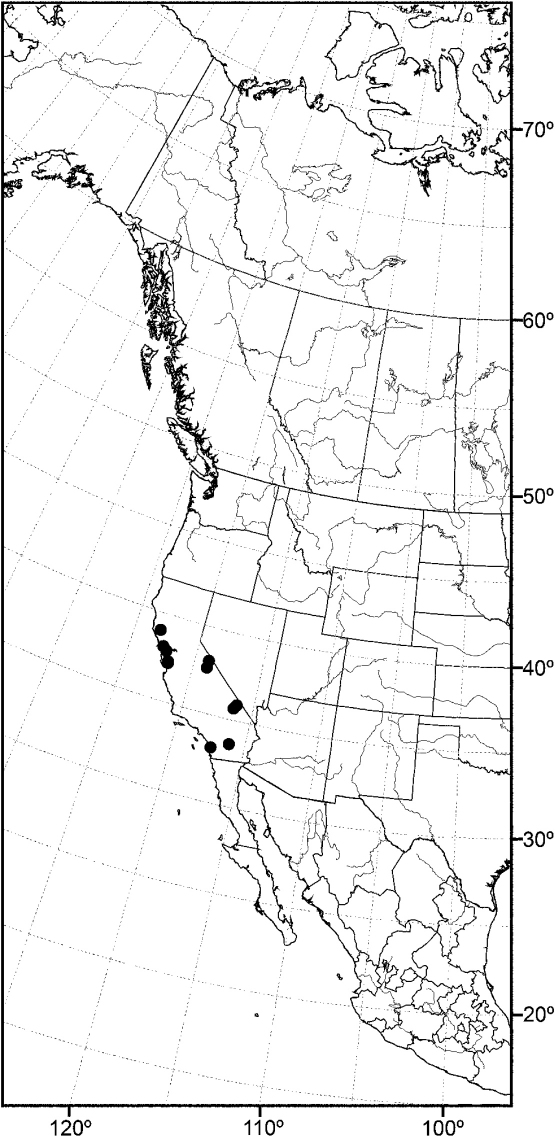
Distribution of *Allotrichoma lasiocercum* Cresson.

(Fig. 38) *Nearctic*: United States (California).

#### Remarks.

Cresson (1929: 176) suggested that a male from Vienna, Austria (Mik), was conspecific with this species. Cresson was probably mistaken and may have confused *Allotrichoma bezzii* with this species. A second Palearctic species, *Allotrichoma picenum* Canzoneri and Rampini (1990: 44; Italy), is similar to *Allotrichoma lasiocercum* but has shorter cerci.

### Allotrichoma (Allotrichoma) occidentale sp. n.

urn:lsid:zoobank.org:act:B2CC0144-B3D3-448B-A8CC-F45E9E9081EA

http://species-id.net/wiki/Allotrichoma_occidentale

Figs 39 44

#### Description.

This species is distinguished from congeners by the following combination of characters: Small to moderately small shore flies, body length 1.60–2.00 mm. *Head*: Medial facial carina above facial prominence shallow; labella broad, fleshy, shorter than mediproboscis; clypeus microtomentose, usually gray.

*Thorax*: Presutural supra-alar seta present. Pleural area lacking stripes. Wing with costal vein ratio 0.27–0.28; M vein ratio 0.41–0.50. Legs blackish brown to black; midtibia bearing numerous, erect setae along dorsal surface.

**Figures 39–43.**
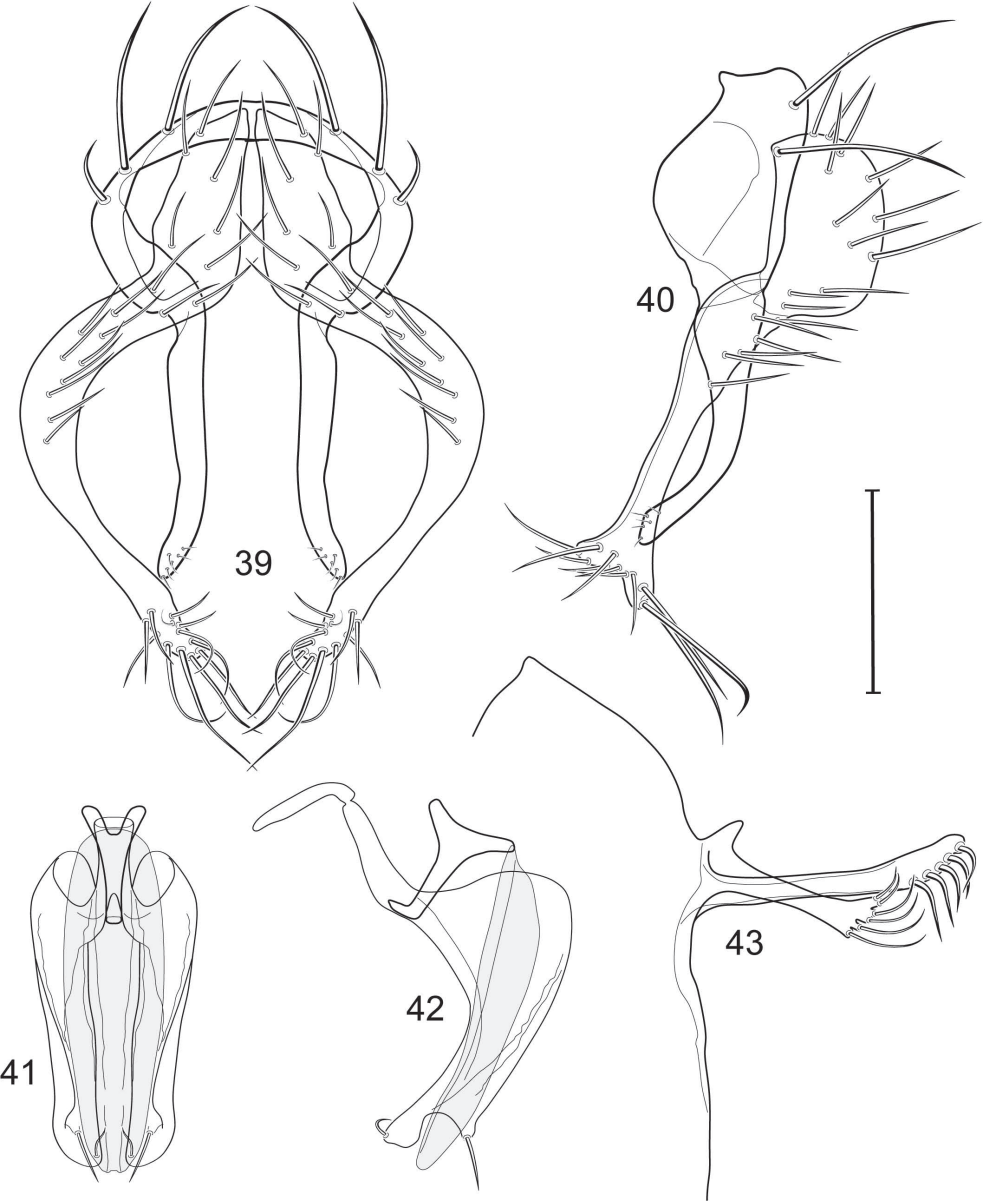
Illustration of *Allotrichoma occidentale* sp. n. (male) **39** epandrium, cerci, surstylus, posterior aspect **40** same, lateral aspect **41** aedeagus, phallapodeme, gonite, hypandrium, ventral aspect **42** same, lateral aspect **43** male 5th sternal flap and medial process, lateral aspect. Scale bar = 0.1 mm.

*Abdomen*: Male 5th sternal flap in lateral view (Fig. 43) narrow, elongate, slightly widened apically, bearing 5–6 apical setulae, some from short tubercles; 5th medial process in lateral view (Fig. 43) relatively narrow, very elongate, apex slightly widened, bearing 4–5 setulae along anteroapical surface. Male terminalia (Figs 39–42): Epandrium in posterior view (Fig. 39) ovately rounded on dorsal half; epandrium in lateral view (Fig. 40) with anterior and posterior margins sinuous, relatively wide dorsally; cercus in posterior view (Fig. 39) broadly pointed dorsally, gradually broadened ventrally then abruptly curved or arched laterally and ventrally as a slightly tapered, elongate, arched process that bears numerous moderately long setulae apically, apex shallowly sinuous; cercus in lateral view (Fig. 40) nearly straight, wider dorsally, ventral half nearly parallel sided, apex broadened and shallowly bifurcate, bearing moderately long setulae; surstyli (ventral extensions of epandrium) in posterior view as parallel, narrow processes that bear setulae on apical portion; surstylus in lateral view (Fig. 40) elongate, narrow, nearly parallel sided, apical portion bearing very short setulae anteriorly and apically, with a short, lacking an apical process; aedeagus in ventral view (Fig. 41) elongate, narrowly ovate, tapered apically, in lateral view (Fig. 42) elongate, somewhat lunate; phallapodeme in lateral view (Fig. 42) triangular, keel shorter than length, narrow, somewhat pointed; gonite in ventral view (Fig. 41) bar-like, slightly wider basally, apex with short, lateral process, apex bearing setula, in lateral view (Fig. 42) wide basally, gently but distinctly arched, tapered to apex, apex with rounded notch, producing a subapical, short, nipple-like process that bear a setula.

#### Type material.

The holotype male is labeled “**USA. OR[EGON].** Lake: Lakeview (27 mi E; Drake Creek; 42°11'N, 119°59.3'W), 29 Jul 2005, D. and W. N. Mathis/HOLOTYPE ♂ *Allotrichoma occidentale* W. Mathis & T. Zatwarnicki USNM [red]/USNM ENT 00117958 [plastic bar code label].” The holotype is double mounted (minuten in a block of plastic), is in excellent condition, and is deposited in the USNM. Twenty-two paratypes (14♂, 8♀; USNM) bear the same locality label as the holotype.

#### Type locality.

United States. Oregon. Lake: Lakeview (44 km E; Drake Creek; 42°11'N, 119°59.3'W).

#### Other specimens examined from the New World.

CANADA. *MANITOBA*. Carberry (13 km NE; 49°51.6'N, 99°20'W), 4 Jul 1984, R. S. and V. L. Zack (1♂; WSU).

*SASKATCHEWAN*. Waskesiu River (53°53.9'N, 106°05.9'W), Aug 1947, R. Coleman (1♂; USNM).

UNITED STATES. *ALASKA. Matanuska-Susitna*: Willow Creek (61°46.1'N, 150°04.2'W; 50 m), 10 Jul 2006, D. and W. N. Mathis (6♂, 7♀; USNM). *Valdez-Cordova (Census Area)*: Gulkana River (19.3 km N Glenallen; 62°16.1'N, 145°23.1'W), 9 Jul-7 Aug 2006, 2011, D. and W. N. Mathis (7♂, 10♀; USNM).

*ARIZONA. Cochise*: Douglas (31°27'N, 109°32.7'W), 8 Jul 1940, R. H. Beamer (1♂; KU).

*CALIFORNIA. Alpine*: Hope Valley (38°46.6'N, 119°56'W), 11 Sep 1938, M. Cazier (2♂, 1♀; USNM). *Calaveras*: Milton (38°01.9'N, 120°51.1'W), 21 Oct 1917, J. C. Bradley (4♂, 2♀; CU). *Fresno*: Coalinga (36°08.1'N, 120°20.4'W), 14 May 1938, M. Cazier (1♂; AMNH). *Imperial*: Calexico (32°40.7'N, 115°29.9'W), 16 Jul 1958, E. I. Schlinger (1♂; USNM). *Kern*: Rosamond (34°51.9'N, 118°11.7'W), 17 Oct 1956, A. H. Sturtevant (2♂; USNM). *Kings*: Lemoore (36°18'N, 119°47'W), 17 Oct 1956, A. H. Sturtevant (2♂; USNM). *Lake*: Clear Lake (38°57.5'N, 122°37.6'W), 11 Oct 1947, W. W. Wirth (1♂; USNM)*. Los Angeles*: Saugus (34°24.7'N, 118°32.4'W), 10 Apr 1953, A. H. Sturtevant (2♂, 1♀; USNM). *Mono*: Mono Lake (S shore; 38°01'N, 119°03.6'W), 12 Aug 1980, R. S. Zack (2♂, 3♀; WSU). *Orange*: Buena Park (33°52.1'N, 117°59.9'W), 19 May 1944, A. L. Melander (3♂; USNM); Capistrano Hot Spring (33°30.1'N, 117°39.7'W), 25 Jan 1945, A. L. Melander (1♂, 4♀; USNM). *Riverside*: Magnesium Spring Canyon (near Indio; 33°43.2'N, 116°13'W), 5 Apr 1945, A. L. Melander (3♂; USNM); Riverside (33°58.5'N, 117°19.7'W), 1 Feb-4 Oct 1934, 1935, A. L. Melander (8♂, 2♀; USNM). *San Bernardino*: Jenks Lake (30°09.9'N, 116°52.9'W), 18–24 Aug 1950, A. L. Melander (1♂, 3♀; USNM); Mountain Home (34°06'N, 117°W), 8 Oct 1956, A. L. Melander (1♂; USNM); Victorville (34°32.2'N, 117°17.5'W), 2 May 1953, R. O. Schuster (1♂; USNM). *San Diego*: Gavilan Hills (33°47'N, 117°22.1'W), 1 Oct 1952, A. L. Melander (1♂, 1♀; USNM). *San Luis Obispo*: Morro Bay (35°20.1'N, 120°51.1'W), 30 Aug 1945, A. L. Melander (4♂; USNM). *San Mateo*: Redwood City (37°29.1'N, 122°14.2'W), 16 Feb 1947, P. H. Arnaud, Jr. (1♂; USNM). *Tulare*: Three Rivers (36°26.3'N, 118°54.3'W), 5 Aug 1940, L. J. Lipovsky (3♂, 1♀; USNM). *Yolo*: Davis (38°32.7'N, 121°44.1'W), 20 Jun-15 Sep 1952, 1966, C. R. Kovacic, A. T. McClay, E. I. Schlinger (9♂, 2♀; USNM); Woodland (38°40.3'N, 121°45'W), 9 Aug 1953, A. T. McClay, E. I. Schlinger (2♂, 1♀; USNM).

*IDAHO. Benewah*: Chatcolet (47°22.3'N, 116°54.8'W), Aug 1915, A. L. Melander (1♂; USNM). *Oneida*: Curlew Valley Reservoir (42°04.5'N, 112°32.3'W), 27 Jun-22 Sep 1969, 1971, G. F. Knowlton (2♂, 1♀; USU); Holbrook (42°09.7'N, 112°39.2'W), 1 Sep 1971, G. F. Knowlton (1♀; USU); Stone (42°01'N, 112°41.7'W), 5 Aug 1969, G. F. Knowlton (1♂; USU); Stone (3.2 km N, 42°10'N, 112°41.7'W), 16 May 1969, G. F. Knowlton (1♂, 1♀; USU); Stone Reservoir (42°04.1'N, 112°41.7'W), 27 Jun 1969, G. F. Knowlton (1♀; USU). *Power*: Rock Creek (42°39.3'N, 113°01.1'W), 17 Jul 1972, G. F. Knowlton (1♀; USU).

*MICHIGAN. Iosco*: (44°20'N, 83°38'W), 3 Jul 1950, G. C. Steyskal (1♂; USNM).

*MONTANA. Granite*: Medicine Lake (48°28.6'N, 113°40.6'W), 9 Jun 1969, W. W. Wirth (1♂; USNM).

*NEBRASKA. Cherry*: Snake River (42°46.8'N, 100°47.7'W), 2 Jun 1960, W. W. Wirth (1♀; USNM); Twin Lakes (42°28.9'N, 100°58.6'W), 2 Jun 1960, W. W. Wirth (1♂; USNM).

*NEVADA. Lander*: Austin (39°28'N, 117°11.7'W), 2 Aug 1956, E. S. Ross (1♂; USNM).

*NORTH DAKOTA. Bowman*: Bowman-Haley Dam and Reservoir (45°59.5'N, 103°15.4'W; 840 m), 19 Jun 2008, D. and W. N. Mathis (8♂, 5♀; USNM).

*OREGON. Baker*: Goose Creek (35 km E Baker City; 44°49.2'N, 117°27.79'W; 825 m), 7 Jun 2006, T. Zatwarnicki (3♂; USNM). *Harney*: Burns (3.2 km E; 43°35.1'N, 119°0.1'W; 1320m), 16 Jun 1972, D. and W. N. Mathis (6♂, 8♀; USNM); King Mountain Lookout turnoff (43°50.6'N, 118°57.2'W; 1537 m), 8 Aug 2005, D. and W. N. Mathis (1♂; USNM); Malheur National Wildlife Refuge, Web Creek (43°16'N, 118°50.7'W), 29 Aug 1983, R. S. Zack (2♂; WSU); Mann Lake (42°46.3'N, 118°26.7'W), 12 Sep 1981, R. S. Zack (4♂; WSU); Page Springs (42°47'N, 118°51'W; 1300 m), 6 Aug 2005, D. and W. N. Mathis (9♂; USNM); Steens Mountain, Fish Lake Campground (42°44.3'N, 118°38.7'W; 2250 m), 6 Aug 2005, D. and W. N. Mathis (3♂; USNM); Wagontire (24 km N, 43°16'N, 119°52.5'W), 12 Jul 1989, R. S. Zack (2♂, 8♀; WSU). *Klamath*: Deming Creek, NE Bly (42°25.6'N, 121°04.4'W), 1 Sep 1959, J. Schuh (2♂, 4♀; USNM); Odell Lake (43°34.4'N, 121°60'W), 26 Jul 1988, R. Danielsson (1♂; ZIL). *Morrow*: Butter Creek (45°37.1'N, 119°26.3'W), 9 Jul 1991, R. S. Zack (3♂; WSU). *Union*: Whiskey Creek (45°19.4'N, 118°14.8'W; 1560 m), 29–31 Aug 1976, E. J. Davis (1♂; WSU).

*SOUTH DAKOTA. Lawrence*: Beaver Creek (44°30.8'N, 104°00.4'W), 15 Jun 1969, W. W. Wirth (2♂, 3♀; USNM).

*UTAH. Box Elder*: Corinne (41°33.1'N, 112°06.6'W; on celery), 8 Aug 1953, G. F. Knowlton (1♂, 1♀; USNM); Bear River Bay (41°21.5'N, 112°21.3'W), 16 Aug 1953, G. F. Knowlton (1♂; USNM). *Cache*: Logan (41°44.1'N, 111°50.1'W), 17 Oct 1943, G. F. Knowlton (1♂; UMSP). *Carbon*: Deadman Canyon (16 km NE Price; 39°41.7'N, 110°44'W; 2055 m), 14–19 Aug 2008, 2009, D. and W. N. Mathis (9♂, 1♀; USNM). *Davis*: Farmington (40°58.8'N, 111°53.3'W), 4 Sep, G. F. Knowlton (1♂; USNM). *Grand*: Thompson Spring (8.9 km N Thompson Springs; 39°02.3'N, 109°43.4'W; 1740 m), 20 Aug 2009, W. N. Mathis, T. Zatwarnicki (5♂; USNM). *Kane*: East Fork of Virgin River (26.6 km N Kanab; 37°12.2'N, 112°41.4'W) 18 May 2001, D. and W. N. Mathis (2♂; USNM). *Salt Lake*: Butterfield Canyon (40°29.2'N, 112°08.2'W; 1890 m), 13 Aug 2008, D. and W. N. Mathis (11♂; USNM); Draper (40°31.6'N, 111°55.1'W; Jordan River; 1320 m), 10 May 2007, D. and W. N. Mathis (3♂; USNM); Midvale (40°36.7'N, 111°54'W), 2 Sep 1953, G. F. Knowlton (1♂; USNM); Salt Lake City (40°45.6'N, 111°53.5'W), 13 Sep 1953, G. F. Knowlton (1♀; USNM). *Sanpete*: Moroni (39°31.6'N, 111°34.4'W), 27 Jun 1940, A. L. Melander (1♂; USNM). *Utah*: Benjamin (40°05.9'N, 111°43.9'W), 21 Jun 1945, G. F. Knowlton (1♂, 1♀; USNM); Goshen Springs (39°57.8'N, 111°51.2'W), 16 Apr 2003, W. N. Mathis, T. Zatwarnicki (5♂, 4♀; USNM); Lake Shore (40°06.9'N, 111°41.8'W; 1370 m), 11 May 2007, D. and W. N. Mathis (4♂; USNM).

*WASHINGTON. Adams*: Lower Crab Creek (46°54.6'N, 119°17.3'W), 28 Aug 1980, R. S. Zack (2♂; WSU). *Benton*: Plymouth (12 km W; 45°56.1'N, 119°24.6'W), 9 Jul 1978, R. S. Zack (2♀; WSU); Snively Spring, Hanford (46°27.1'N, 119°42.8'W), 24 Apr 1995, R. S. Zack (6♂, 11♀; WSU). *Franklin*: Handford Reach, Columbia River (46°24.6'N, 119°15.5'W), 20 Jul 2009, D. Mathis (1♂; USNM); Sacajawea State Park (46°12'N, 119°02.4'W; 100 m), 6 Jun 2006, D. and W. N. Mathis (2♂; USNM). *Grant*: Beverly (Columbia River; 46°50.4'N, 119°56.4'W), 29 May 2006, D. and W. N. Mathis (1♂, 1♀; USNM); Beverly Dunes (3.2 km E Beverly; 46°49.7'N, 119°53.7'W), 30 May 2006, D. and W. N. Mathis (2♂; USNM); Columbia National Wildlife Refuge (46°51.1'N, 119°32.2'W), 13 May 1959, H. F. Harwood (1♂; WSU); Crab Creek (4.8 km E Beverly; 46°50'N, 119°52'W), 30 May 2006, D. and W. N. Mathis (20♂, 7♀; USNM); Othello (3.2 km W; 46°49.6'N, 119°13.4'W), 25 Jul 1992, R. S. Zack (11♂; WSU); Royal City (8 km W; 46°54'N, 119°47'W), 13 Jul 1978, R. S. Zack (23♂, 21♀; WSU). *Kittitas*: Vantage (Columbia River; 46°56.5'N, 119°59.1'W), 29 May 2006, D. and W. N. Mathis (1♂; USNM). *Lincoln*: Wilbur Pond (47°45.6'N, 118°42.3'W), 8 Jul 1993, R. S. Zack (3♂; WSU). *Okanogan*: Little Goose Lake (48°16.5'N, 119°31'W), 22 Jul 1983, R. D. Akre, R. S. Zack (5♂; WSU); Okanogan (15 km SW, Soap Lake; 48°14.1'N, 119°39'W), 23 Jul 1983, R. D. Akre, R. S. Zack (6♂, 8♀; WSU). *Spokane*: Elokia Lake (48°01.6'N, 117°22.7'W), 24 Jun 1982, R. D. Akre, R. S. Zack (2♂; WSU). *Whitman*: Rock Lake (47°11.1'N, 117°40.9'W), 25 Jun 1982, R. D. Akre, R. S. Zack (1♂; WSU); Union Flat (46°49.6'N, 117°59.9'W), 3 Jul 1918 (1♂; USNM). *Yakima*: Prosser (7.6 km W; 46°11.3'N, 119°53'W), 14 Jun 1989, R. S. Zack (4♂, 3♀; WSU).

*WYOMING. Albany*: Laramie (41°18.5'N, 105°36.7'W), 9 Jul 1956 (1♂; USNM). *Carbon*: Elk Mountain (5 km W; 41°41.1'N, 106°26.4'W), 20 Aug 1982, R. S. Zack (2♂, 8♀; WSU).

#### Distribution.

**Figure 44.**
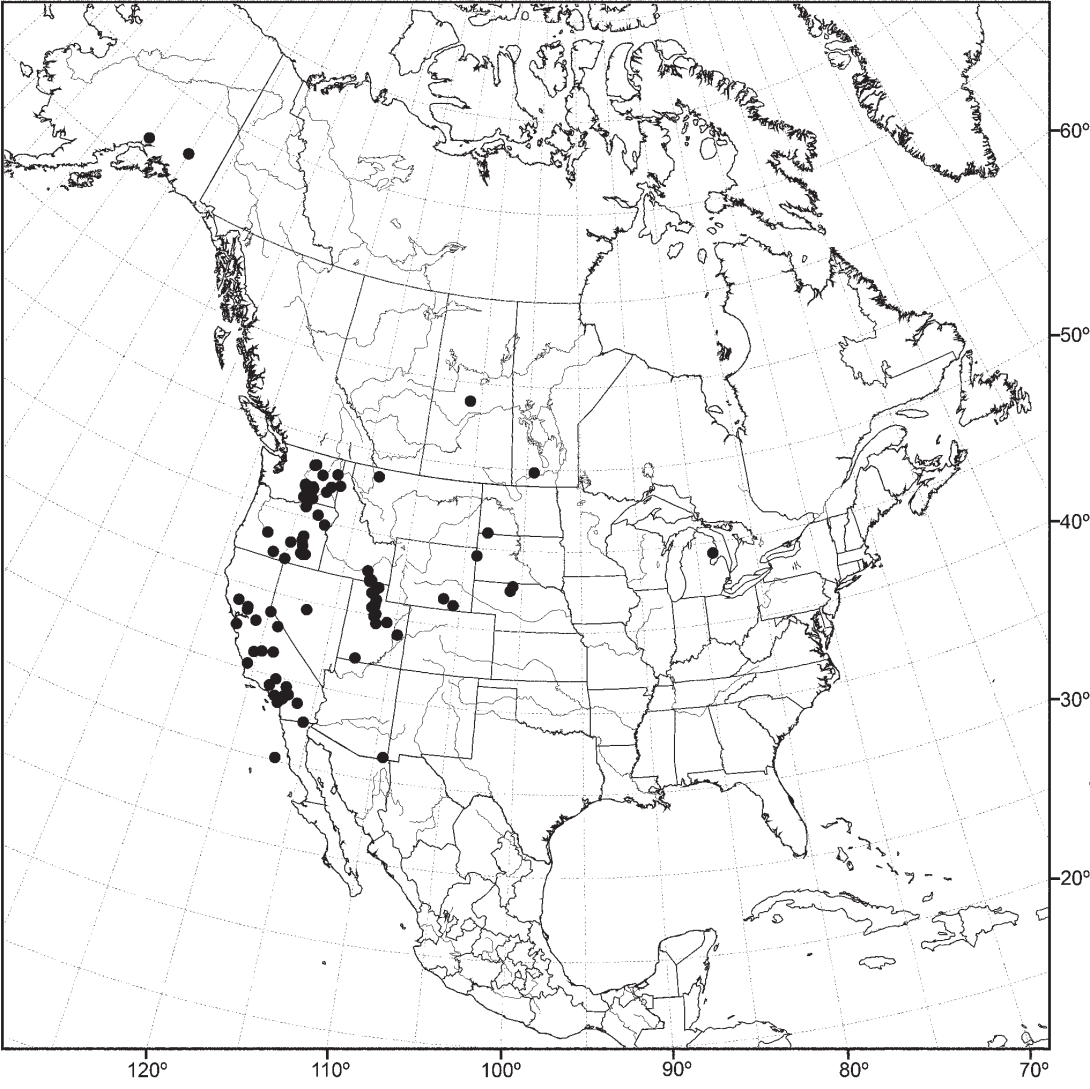
Distribution of *Allotrichoma occidentale* sp. n.

(Fig. 44) *Nearctic*: Canada (Manitoba, Saskatchewan), United States (Alaska, Arizona, California, Idaho, Michigan, Montana, Nebraska, Nevada, North Dakota, Oregon, South Dakota, Utah, Washington, Wyoming).

#### Etymology.

The species epithet, *occidentale*, refers to the western distribution of this species in the Nearctic Region.

#### Remarks.

This species is closely related to *Allotrichoma bifidum* Papp (1975), which was relatively recently described from specimens collected in Hungary. The morphological differences between these two species are relatively slight but consistent and are especially evident in the shape of the extended cercus. The distal apex of the cercus in *Allotrichoma occidentale* is only slightly flared; whereas in *Allotrichoma bifidum* the apex is conspicuously and consistently much wider. Given these consistent differences and its pronounced allopatry with *Allotrichoma bifidum*, we have elected to treat this species as separate and distinct.

### Allotrichoma (Allotrichoma) robustum sp. n.

urn:lsid:zoobank.org:act:57B87C6E-70B0-4D1F-B9F4-0F1FE887C307

http://species-id.net/wiki/Allotrichoma_robustum

Figs 45 50

Allotrichoma laterale of authors, not Loew 1860 [misidentification]. Becker 1896: 121 [generic combination]. Williston 1897: 4 [compared with *Allotrichoma abdominale* Williston]. Cresson 1942: 108 [list, west coast of United States]. Wirth and Stone 1956: 467 [key]. Wirth 1965: 736 [Nearctic catalog, in part]. Cole 1969: 397 [list]. Mathis and Zatwarnicki 1995: 151–152 [world catalog, in part].

#### Description.

This species is distinguished from congeners by the following combination of characters: Small to moderately small shore flies, body length 1.55–2.40 mm. *Head*: Medial facial carina above facial prominence shallow; labella broad, fleshy, shorter than mediproboscis; clypeus microtomentose, usually gray.

*Thorax*: Presutural supra-alar seta present. Pleural area lacking stripes. Wing with costal vein ratio 0.30–0.31; M vein ratio 0.37–0.42. Legs blackish brown to black; midtibia bearing numerous, erect setae along dorsal surface.

**Figures 45–49.**
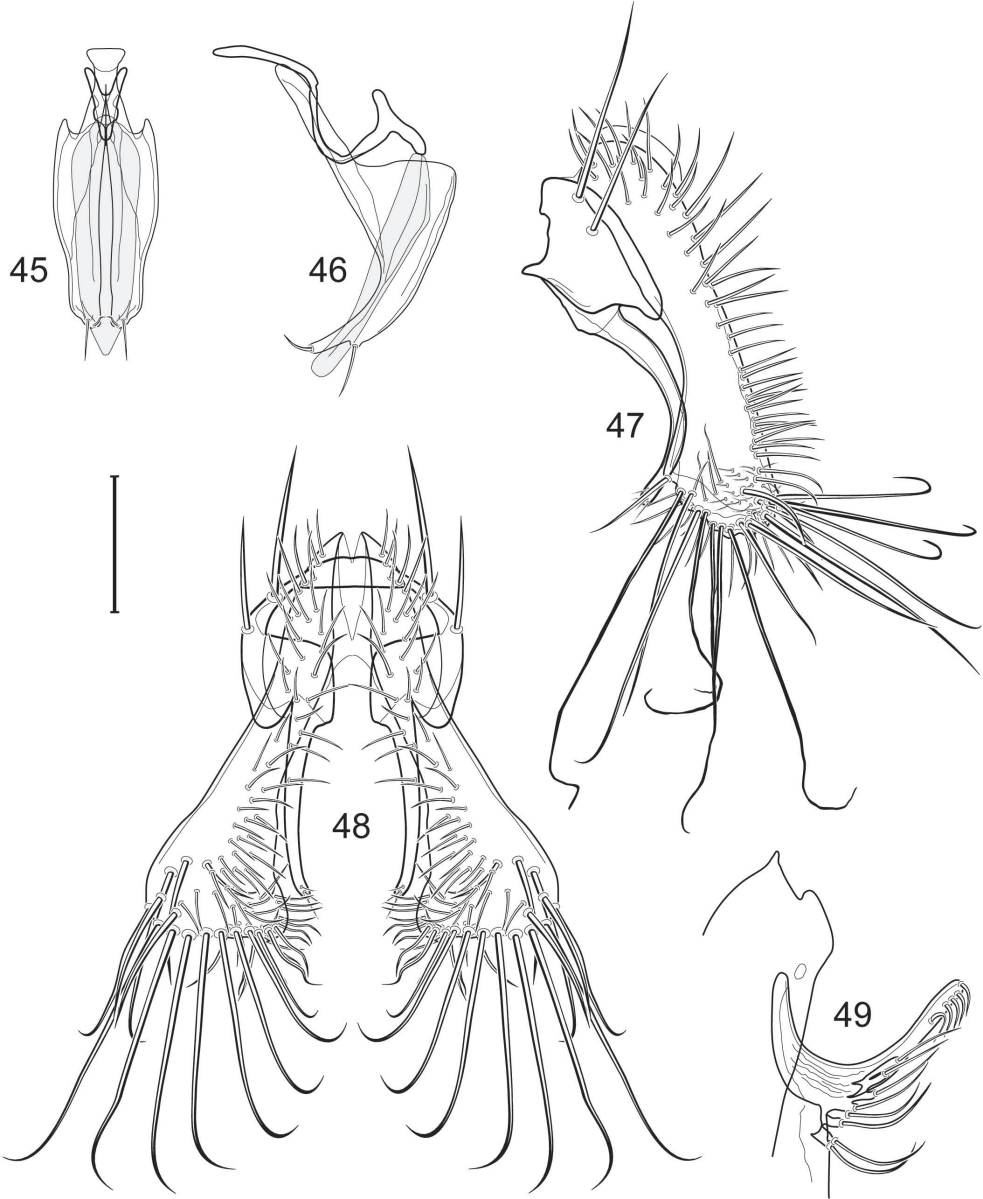
Illustration of *Allotrichoma robustum* sp. n. (male) **45** aedeagus, phallapodeme, gonite, hypandrium, ventral aspect **46** same, lateral aspect **47** epandrium, cerci, surstylus, lateral aspect **48** same, posterior aspect **49** male 5th sternal flap and medial process, lateral aspect. Scale bar = 0.1 mm.

*Abdomen*: Male 5th sternal flap in lateral view (Fig. 49) moderately shallow, longer posteriorly, bearing several, tuberculate setulae apically; 5th medial process in lateral view (Fig. 49) elongate, conspicuously curved, C-shaped, bearing 4–5 setulae anteroapically. Male terminalia (Figs 45–48): Epandrium in posterior view (Fig. 45) like an inverted, laterally rounded, almost oval U; epandrium in lateral view (Fig. 46) relatively narrow dorsally, becoming wider ventrally than tapered to a broad point; cercus very robust, in posterior view (Fig. 45) as a elongate, pendulous, ventrolaterally projected process, becoming wider ventrally, apex flared, truncate, bearing numerous, long to very long setulae, very long setulae apicolaterally; cercus in lateral view (Fig. 46) essentially straight, widest subapically, then narrowed to blunt, ventral apex, some very long setulae curved apically or irregularly contorted; surstyli (ventral extensions of epandrium) in posterior view as nearly parallel, shallowly arched medially, narrow processes that bears a few setulae apically, surstylus in lateral view (Fig. 46) conspicuously arched, narrow, very slightly tapered process, not expanded apically; aedeagus in ventral view (Fig. 47) elongate, narrowly ovate, slightly tapered apically, in lateral view (Fig. 48) elongate, narrow, nearly straight; phallapodeme in lateral view (Fig. 48) narrowly triangular, keel narrow, prong-like, shorter than length, almost digitiform; gonite in ventral view (Fig. 47) somewhat bar-like, lateral margin shallowly sinuous, notched basally and medially pointed apically, bearing a subapical, ventral setula and an apical setula; gonite in lateral view (Fig. 47) wide basally, conspicuously but gently curved and tapered toward apex, with a subapical, setula and an apical setula.

#### Type material.

The holotype male is labeled “Kern Riv. 4 - 1- [19]50 [handwritten by A. H. Sturtevant]/HOLOTYPE ♂ *Allotrichoma robustum* W. Mathis & T. Zatwarnicki USNM [red]/USNM ENT 00117959 [plastic bar code label].” The holotype male is double mounted (glued to a narrow, paper triangle), is in good condition (abdomen removed, dissected, stored in glycerin in an attached microvial), and is deposited in the USNM. Two paratypes are as follows: UNITED STATES. *CALIFORNIA. Kern*: Kern River (35°16.1'N, 119°18.4'W), 1 Apr 1950, A. H. Sturtevant (1♂; USNM). *Nevada*: Sagehen Creek (39°28.6'N, 120°07.9'W), 26 Jun 1974, R. L. Bugg (1♂; USNM). *OREGON. Harney*: (43°10'N, 119°0.1'W), 29 Jun 1953, A. B. Gurney (1♂; USNM).

#### Type locality.

United States. California. Kern: Kern River (35°16.1'N, 119°18.4'W).

#### Distribution.

**Figure 50.**
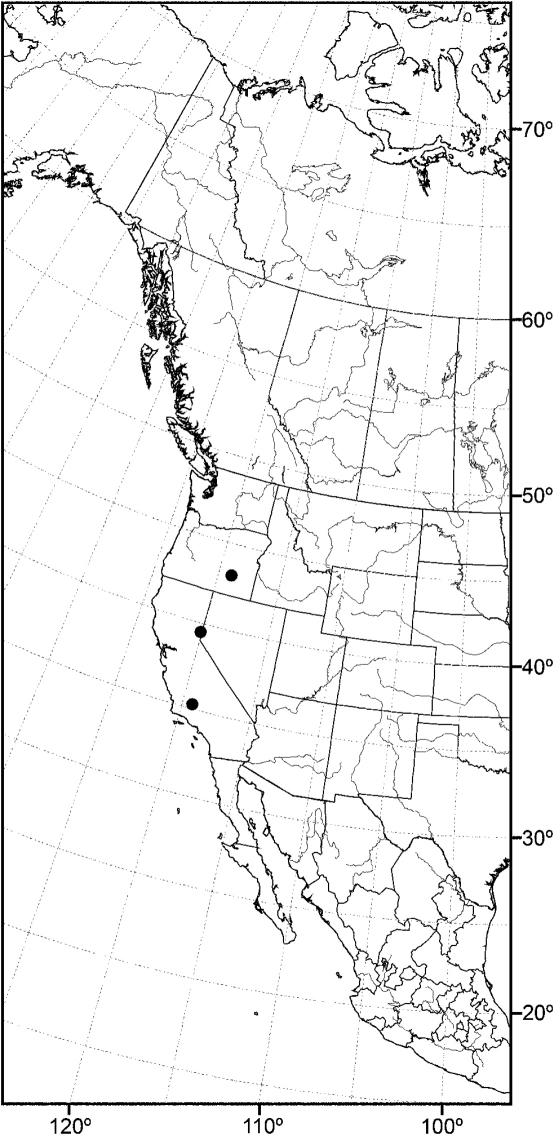
Distribution of *Allotrichoma robustum* sp. n.

(Fig. 50) *Nearctic*: United States (California, Oregon).

#### Etymology.

The species epithet, *robustum*, is of Latin derivation and refers to the very robust cerci of this species.

#### Remarks.

Among Nearctic species of *Allotrichoma*, *Allotrichoma robustum* has the most restricted distribution, being thus far known only from the Sierra Mountains in California and Harney County in Oregon.

### Allotrichoma (Allotrichoma) sabroskyi sp. n.

urn:lsid:zoobank.org:act:249D460F-3FAC-4C9B-8BC7-F49D95DD4360

http://species-id.net/wiki/Allotrichoma_sabroskyi

Figs 51 56

#### Description.

This species is distinguished from congeners by the following combination of characters: Small shore flies, body length 1.40–1.75 mm. *Head*: Medial facial carina above facial prominence shallow; labella broad, fleshy, shorter than mediproboscis; clypeus microtomentose, usually gray.

*Thorax*: Presutural supra-alar seta present. Pleural area lacking stripes. Wing with costal vein ratio 0.33–0.36; M vein ratio 0.43–0.44. Legs blackish brown to black; midtibia bearing numerous, erect setae along dorsal surface.

**Figures 51–55.**
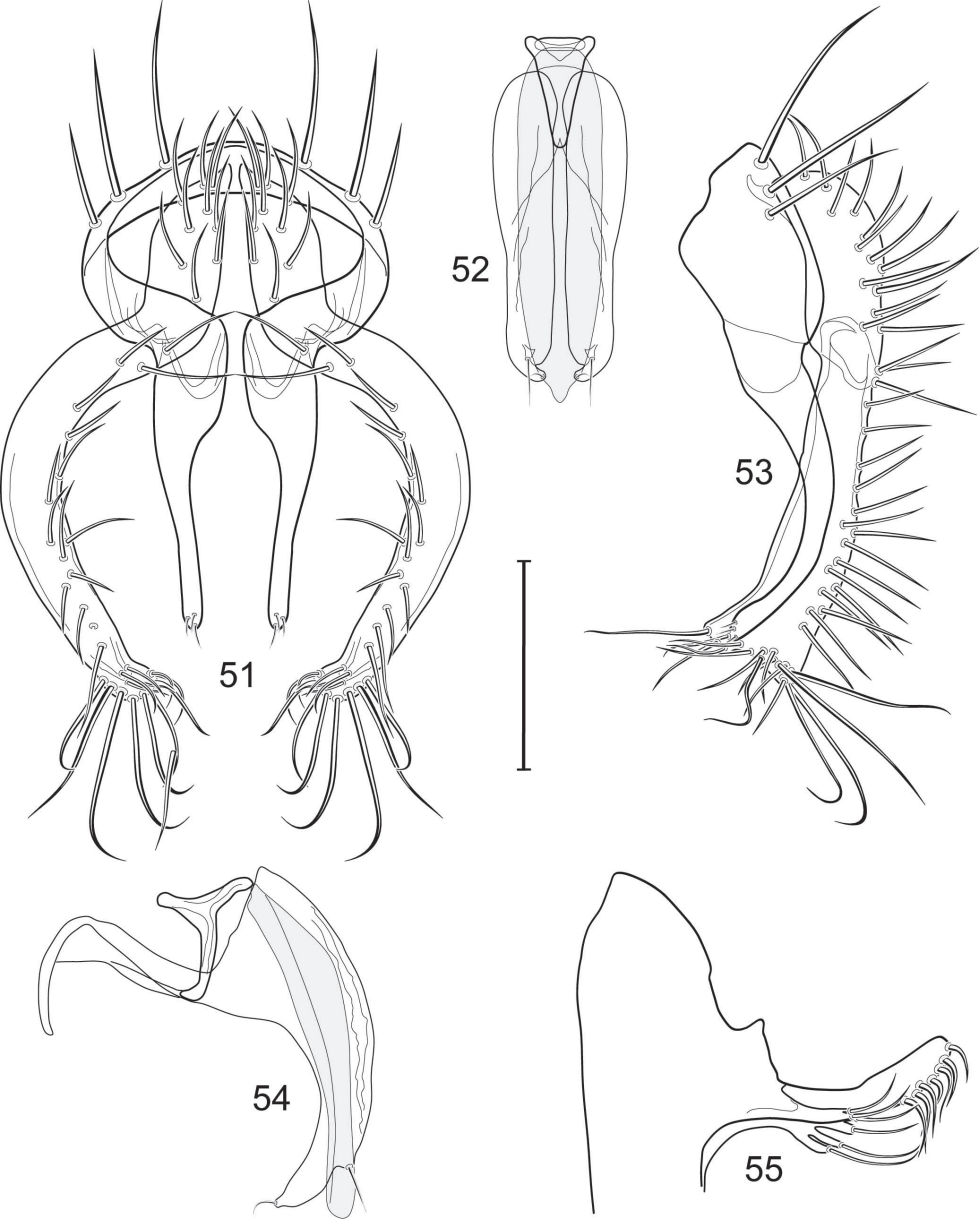
Illustration of *Allotrichoma sabroskyi* sp. n. (male) **51** epandrium, cerci, surstylus, posterior aspect **52** aedeagus, phallapodeme, gonite, hypandrium, ventral aspect **53** epandrium, cerci, surstylus, lateral aspect **54** aedeagus, phallapodeme, gonite, hypandrium, lateral aspect **55** male 5th sternal flap and medial process, lateral aspect. Scale bar = 0.1 mm.

*Abdomen*: Male 5th sternal process in lateral view (Fig. 55) moderately elongate, narrow, bearing 4–5 tuberculate setulae apically; 5th medial process in lateral view (Fig. 55) elongate, shallowly curved, bearing 7–8 setulae anteroapically. Male terminalia (Figs 51–54): Epandrium in posterior view (Fig. 51) ovately rounded on dorsal half, wider than high; epandrium in lateral view (Fig. 52) with anterior and posterior margins of dorsal half nearly straight and parallel sided; cercus robust, thick, in posterior view (Fig. 51) pointed dorsally, gradually broadened ventrally then rather abruptly curved laterally and ventrally as a slightly tapered, elongate process that bears numerous long setulae apically and along posterior margin; cercus in lateral view (Fig. 52) slightly curved, wider dorsally, ventral half nearly parallel sided, apex broadened and bearing setulae; surstyli in posterior view (Fig. 51) as parallel, narrow, elongate processes that bear setulae on apical portion, apex narrowly rounded; surstylus in lateral view (Fig. 52) shallowly curved, basal half slightly tapered, apical half almost parallel sided, apical portion bearing short setulae, apex tapered to narrow point; aedeagus in ventral view (Fig. 53) elongate, moderately narrowly ovate, tapered apically, in lateral view (Fig. 54) narrowly elongate, somewhat lunate; phallapodeme in lateral view (Fig. 54) very narrowly triangular, digitiform keel shorter than length, pointed; gonite in ventral view (Fig. 53) bar-like, very slightly wider basally, apex with short, lateral process, apex bearing setula, in lateral view (Fig. 54) wide basally, gently curved, tapered to apical point, with a subapical, short process that bears a setula.

#### Type material.

The holotype male is labeled “**USA. N[ew]M[exico].** Sandoval: LaCueva(Hhwys 126&4; 35°52'N, 106°38.4'W; 2342 m), 6 Aug 2007,D.&W.N.Mathis/ HOLOTYPE ♂ *Allotrichoma sabroskyi* W. Mathis & T. Zatwarnicki USNM [red]/USNM ENT 00117960 [plastic bar code label].” The holotype is double mounted (minuten in a block of plastic elastomer), is in excellent condition, and is deposited in the USNM. Forty-five paratypes (44♂, 1♀; USNM) bear the same locality label as the holotype.

#### Type locality.

United States. New Mexico. Sandoval: La Cueva (Junction of Highways 126 and 4; 35°52'N, 106°38.4'W; 2342 m).

#### Other specimens examined from the New World.

CANADA. *BRITISH COLUMBIA*. Wasa Lake (49°47.4'N, 115°44.1'W), 17 Jul 1974, P. H. Arnaud, Jr. (1♂, 1♀; CAS).

UNITED STATES. *ARIZONA. Coconino*: Flagstaff (35°N, 111°44.3'W), 10 Jun 1909 (1♂; USNM); Oak Creek (18 km N Sedona; 35°N, 111°44.3'W), 13 Apr 2003, W. N. Mathis, T. Zatwarnicki (9♂, 5♀; USNM).

*CALIFORNIA. Amador*: Buena Vista (38°17.4'N, 120°54.8'W), F. R. Cole (1♂; USNM). *Santa Clara*: San Jose (37°20.4'N, 121°53.7'W), 8 Sep 1949, L. W. Quate (2♀; USNM).

*COLORADO. Archuleta*: Pagosa Springs (37°15.8'N, 107°00.7'W), 27 May 1969, W. W. Wirth (2♂; USNM). *Dolores*: Cahone (9 km N, Dolores River; 37°38.9'N, 108°44.1'W; 1955 m), 3 Aug 2007, D. and W. N. Mathis (2♂; USNM).

*KANSAS. Rooks*: Webster Dam (39°24.5'N, 99°25.5'W), 1 Sep 1961, D. L. Deonier (1♂; USNM).

*MONTANA. Lake*: Big Fork (13 km NE; 47°50'N, 113°53'W), 9 Aug 1969, B. A. Foote (2♂; USNM); Dayton (47°52'N, 114°16.7'W), 13 Jul 1935, A. L. Melander (1♂; USNM).

*NEW MEXICO. Sandoval*: Valles Caldera National Preserve, Alamo Canyon (middle pond; 35°54.9'N, 106°35'W; 2645 m), 4 Aug 2008, D. and W. N. Mathis (1♂; USNM). *Taos*: Rio Grande (36°18.3'N, 105°46.1'W), 6 Jul 1953, W. W. Wirth (1♂; USNM).

*OREGON. Benton*: Corvallis, Dixon Creek (44°34.5'N, 123°15.2'W), 24 Aug 1974, W. N. Mathis (1♂; USNM).

*UTAH. Carbon*: Deadman Canyon (16 km NE Price; 39°41.7'N, 110°44'W; 2055 m), 19 Aug 2009, T. Zatwarnicki (1♂; USNM). *Kane*: Kanab (37°02.8'N, 112°31.6'W), 14 Jul 1948, G. F. Knowlton (1♂; USNM). *Salt Lake*: Butterfield Canyon (40°29.2'N, 112°08.2'W; 1890 m), 13 Aug 2008, D. and W. N. Mathis (1♂; USNM)*. San Juan*: Johnson Canyon (21 km W Blanding: 37°47.6'N, 109°30.7'W; 2335 m), 2 Aug 2007, D. and W. N. Mathis (1♂; USNM).

#### Distribution.

**Figure 56.**
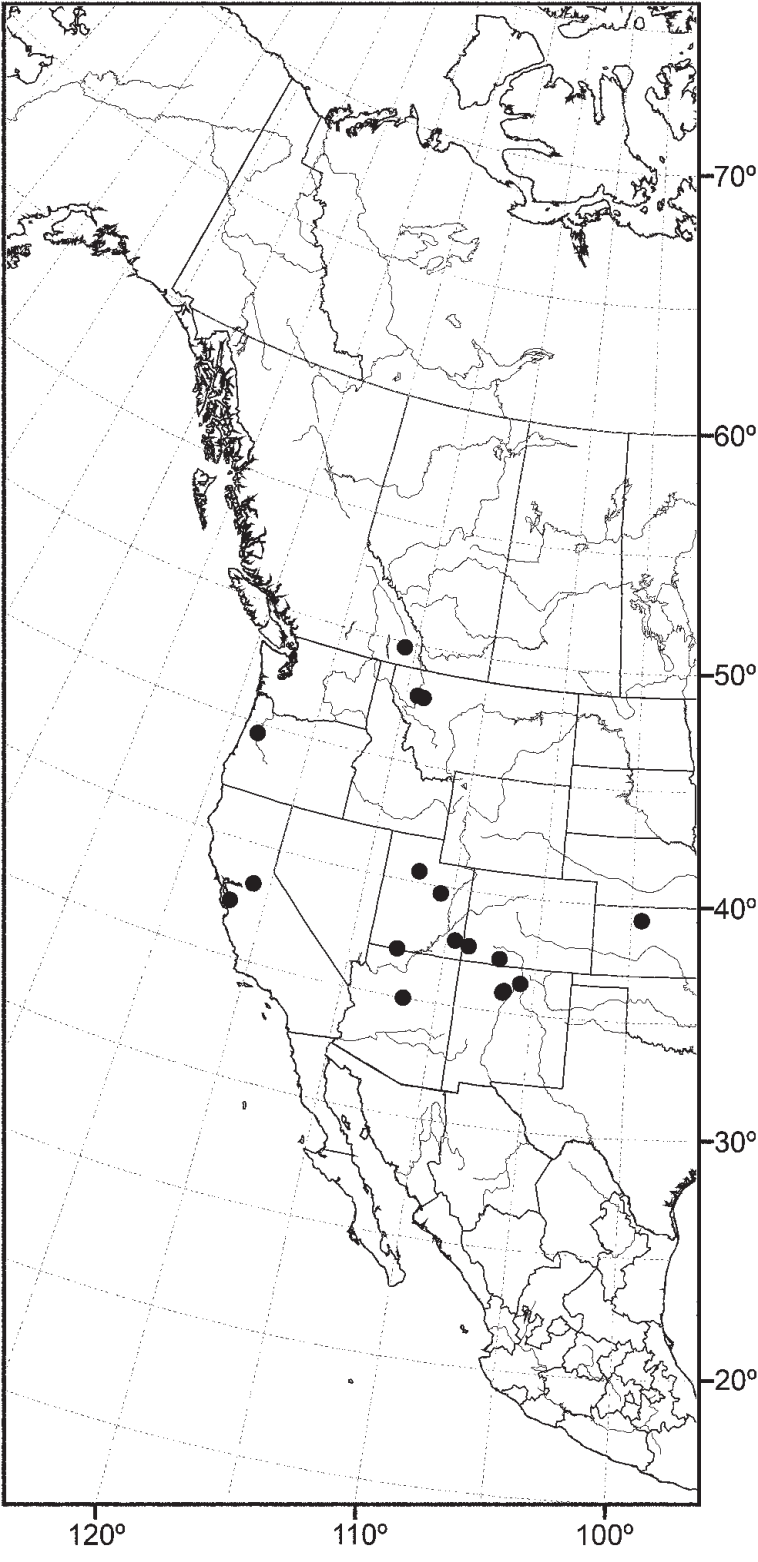
Distribution of *Allotrichoma sabroskyi* sp. n.

(Fig. 56) *Nearctic*: Canada (British Columbia), United States (Arizona, California, Colorado, Kansas, Montana, New Mexico, Oregon, Utah).

#### Etymology.

The species epithet, *sabroskyi*, is a genitive patronym to honor Curtis W. Sabrosky. Curt was our mentor and friend and always had time to assist and respond to our numerous questions.

### Allotrichoma (Allotrichoma) schumanni

Papp

http://species-id.net/wiki/Allotrichoma_schumanni

Figs 57 62

Allotrichoma laterale of authors, not Loew 1860 [misidentification in part]. Becker 1896: 121 [generic combination]. Williston 1897: 4 [compared with *Allotrichoma abdominale* Williston]. Cresson 1942: 108 [list, west coast of United States]. Wirth and Stone 1956: 467 [key]. Wirth 1965: 736 [Nearctic catalog, in part]. Cole 1969: 397 [list]. Mathis and Zatwarnicki 1995: 151-152 [world catalog, in part].
Allotrichoma schumanni Papp 1974: 405 [Hungary. Gyón; HT ♂, HNHM]. Mathis and Zatwarnicki 1995: 153 [world catalog].

#### Description.

This species is distinguished from congeners by the following combination of characters: Small to moderately small shore flies, body length 1.40–2.00 mm. *Head*: Medial facial carina above facial prominence shallow; labella broad, fleshy, shorter than mediproboscis; clypeus microtomentose, usually gray.

*Thorax*: Presutural supra-alar seta present. Pleural area lacking stripes. Wing with costal vein ratio 0.30–0.33; M vein ratio 0.37–0.46. Legs blackish brown to black; midtibia bearing numerous, erect setae along dorsal surface.

**Figures 57–61.**
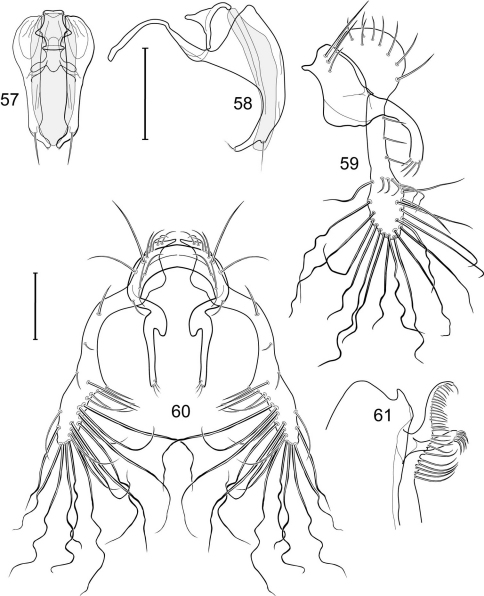
Illustration of *Allotrichoma schumanni* Papp (male) **57** aedeagus, phallapodeme, gonite, hypandrium, ventral aspect **58** same, lateral aspect **59** epandrium, cerci, surstylus, lateral aspect **60** same, posterior aspect **61** male 5th sternal flap and medial process, lateral aspect. Scale bar = 0.1 mm.

*Abdomen*: Male 5th sternal flap in lateral view (Fig. 61) moderately and irregularly elongate, bearing several, tuberculate setulae apically; 5th medial process in lateral view (Fig. 61) elongate, conspicuously curved, C-shaped, bearing 5–6 setulae anteroapically. Male terminalia (Figs 57–60): Epandrium in posterior view (Fig. 57) like an inverted, laterally rounded, almost oval U; epandrium in lateral view (Fig. 58) relatively narrow dorsally, becoming wider ventrally, slightly projected posteroventrally; cercus in lateral view (Fig. 58) essentially straight, wide dorsally, than narrowed but very gradually becoming wider ventrally, as wide subapically as dorsal width, then tapered more abruptly to narrowly rounded apex, apex bearing long and apically curved setulae especially evident on ventral margin; cercus in posterior view (Fig. 57) as a elongate, somewhat pendulous, ventrally projected process, gradually becoming wider ventrally, apex truncate to very shallowly emarginate at an oblique angle, apex slightly longer laterally than medially, apex bearing numerous, very long setulae; surstyli (ventral extensions of epandrium) in posterior view as parallel, narrow processes that bear setulae on apex, apex not broadened or curved; surstylus in lateral view narrow, elongate, conspicuously curved, apical portion not expanded, bearing setulae apically; aedeagus in ventral view (Fig. 59) elongate, narrowly ovate, slightly tapered apically, in lateral view (Fig. 60) elongate, shallowly and narrowly lunate; phallapodeme in lateral view (Fig. 60) narrowly triangular, keel shorter than length, almost digitiform; gonite in ventral view (Fig. 59) somewhat bar-like, lateral margin very shallowly concave, with short, subapical, medial process, apex of process bearing a setula, and a short, apical process that likewise bears a short setula; gonite in lateral view (Fig. 60) wide basally, gently curved and tapered toward apex, with a subapical, short process that bears a setula and an apical setula.

#### Type material.

The holotype male is labeled “Gyón [Hungary] Kertész [handwritten] on the reverse: “1904. VII. 8” [on reverse]/Allotrichoma n. sp. det. Becker [“Allotrichoma n. sp.” handwritten]/Holotypus Allotrichoma schumanni ♂ L. Papp [Holotypus red; species name handwritten; margin of label red].” The holotype is double mounted, is in good condition, and is deposited in the HNHM.

#### Type locality.

Hungary. Gyón (47°11'N, 19°19'E).

#### Other specimens examined from the New World.

UNITED STATES. *COLORADO*. *Archuleta*: Pagosa Springs (37°15.8'N, 107°00.7'W), 27 May 1969, W. W. Wirth (1♂, 4♀; USNM). *Boulder*: Middle Boulder Creek (16 km W Boulder; 40°00.3'N, 105°24.4'W; 2280 m), 8 Aug 1972, P. H. Arnaud, Jr. (6♂, 8♀; CAS, USNM).

#### Distribution.

**Figure 62.**
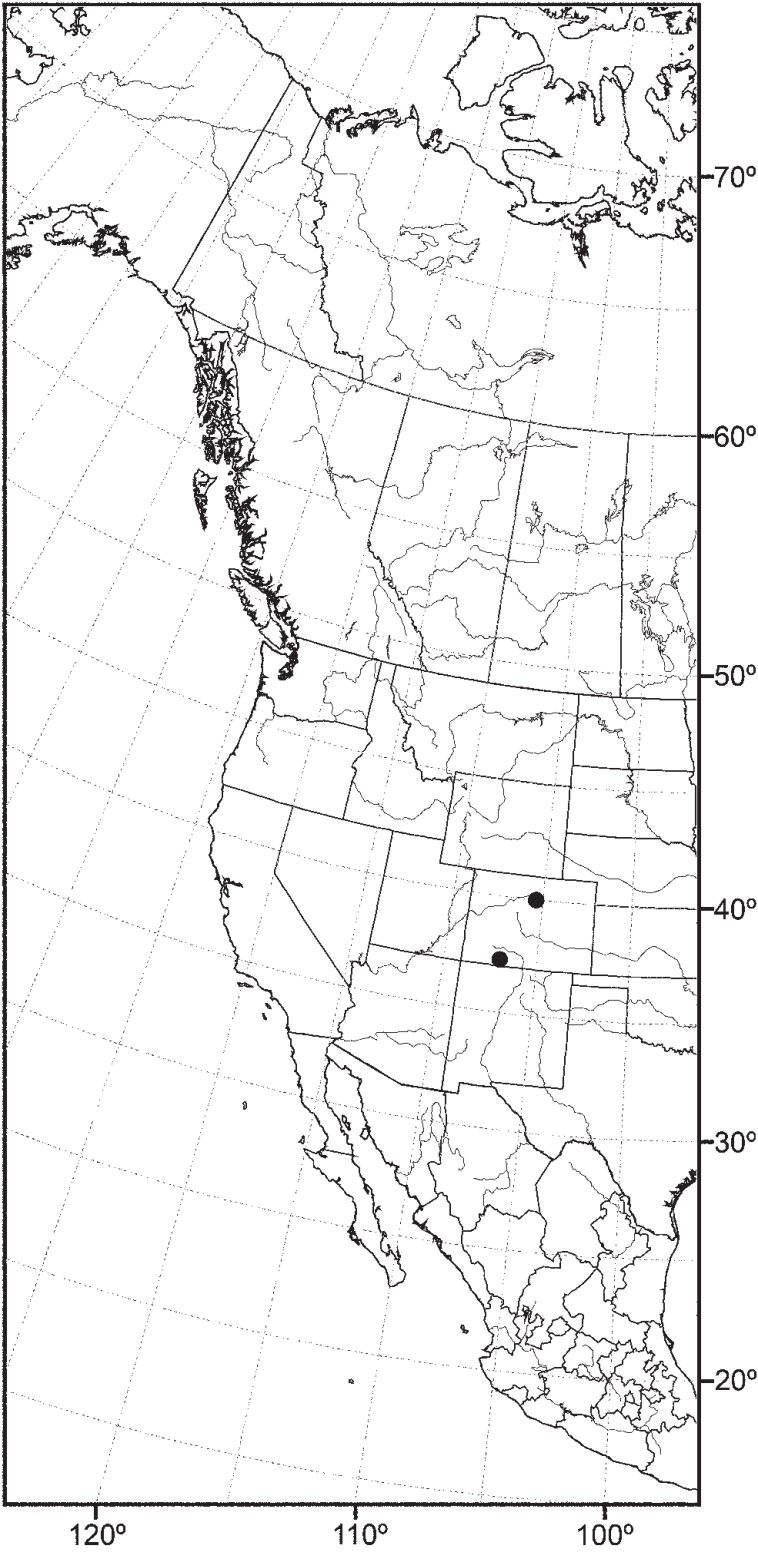
Distribution of *Allotrichoma schumanni* Papp.

(Fig. 62) *Nearctic*: United States (Colorado). Palearctic: Belgium, Czech Republic, Hungary, Italy, Poland, Switzerland.

### Allotrichoma (Allotrichoma) simplex

(Loew)

http://species-id.net/wiki/Allotrichoma_simplex

Figs 63 68

Discocerina simplex Loew 1861: 354. Osten Sacken 1878: 201 [Nearctic catalog]. Aldrich 1905: 626 [Nearctic catalog]. Jones 1906: 178 [key, list].
Allotrichoma simplex . Johnson 1925: 271 [generic combination; list, Massachusetts]. Cresson 1942: 108 [list, general distribution in North America]; 1949: 260 [synonymy]. Wirth and Stone 1956: 467 [key, California]. Deonier 1964: 120 [key, Iowa]; 1965: 501 [natural history]. Wirth 1965: 736 [Nearctic catalog]. Cole 1969: 397 [discussion, western United States]. Runyan and Deonier 1979: 123–137 [discussion, Figs of head and immature stages]. Mathis and Zatwarnicki 1995: 153 [world catalog]. Mathis and Mathis 2008: 181–182 [shore flies of Plummers Island].
Allotrichoma filiforme Becker 1896: 123 [Russia. “Sarepta” (= Volgograd-Krasnoarmejsk); LT ♂ (designated by Papp 1979: 98), ZMHU]; 1905: 189 [Palearctic catalog]. Krivosheina and Zatwarnicki 1997: 301–303 [revision]. New Synonym
Allotrichoma trispinum Becker 1896: 124 [Poland. “Oderwalde dei Maltsch, Schlesien” [= Malczyce, Lower Silesia]; LT ♂ (designated by Papp 1979: 99), ZMHU]; 1905: 189 [Palearctic catalog]. Krivosheina and Zatwarnicki 1997: 301 [synonymy with *Allotrichoma filiforme* Becker]. New Synonym
Allotrichoma dahli Beschovski 1966b: 937 [Bulgaria. Kap Galata; HT ♂, ZMS]. Papp 1979: 98 [synonymy with *Allotrichoma filiforme* Becker]. New Synonym
Allotrichoma trispinum of authors, not Becker, 1896 [misidentification]. Johnson 1925: 271 [list, Connecticut]. Wirth 1965: 736 [Nearctic catalog]. Wirth and Stone 1956: 467 [key]. Cole 1969: 397 [list]. Mathis and Zatwarnicki 1995: 154 [world catalog].

#### Description.

This species is distinguished from congeners by the following combination of characters: Small to moderately small shore flies, body length 1.75–2.15 mm. *Head*: Medial facial carina above facial prominence shallow; labella broad, fleshy, shorter than mediproboscis; clypeus microtomentose, usually gray.

*Thorax*: Presutural supra-alar seta present. Pleural area lacking stripes. Wing with costal vein ratio 0.25–0.31; M vein ratio 0.42–0.50. Legs blackish brown to black; midtibia bearing numerous, erect setae along dorsal surface.

**Figures 63–67.**
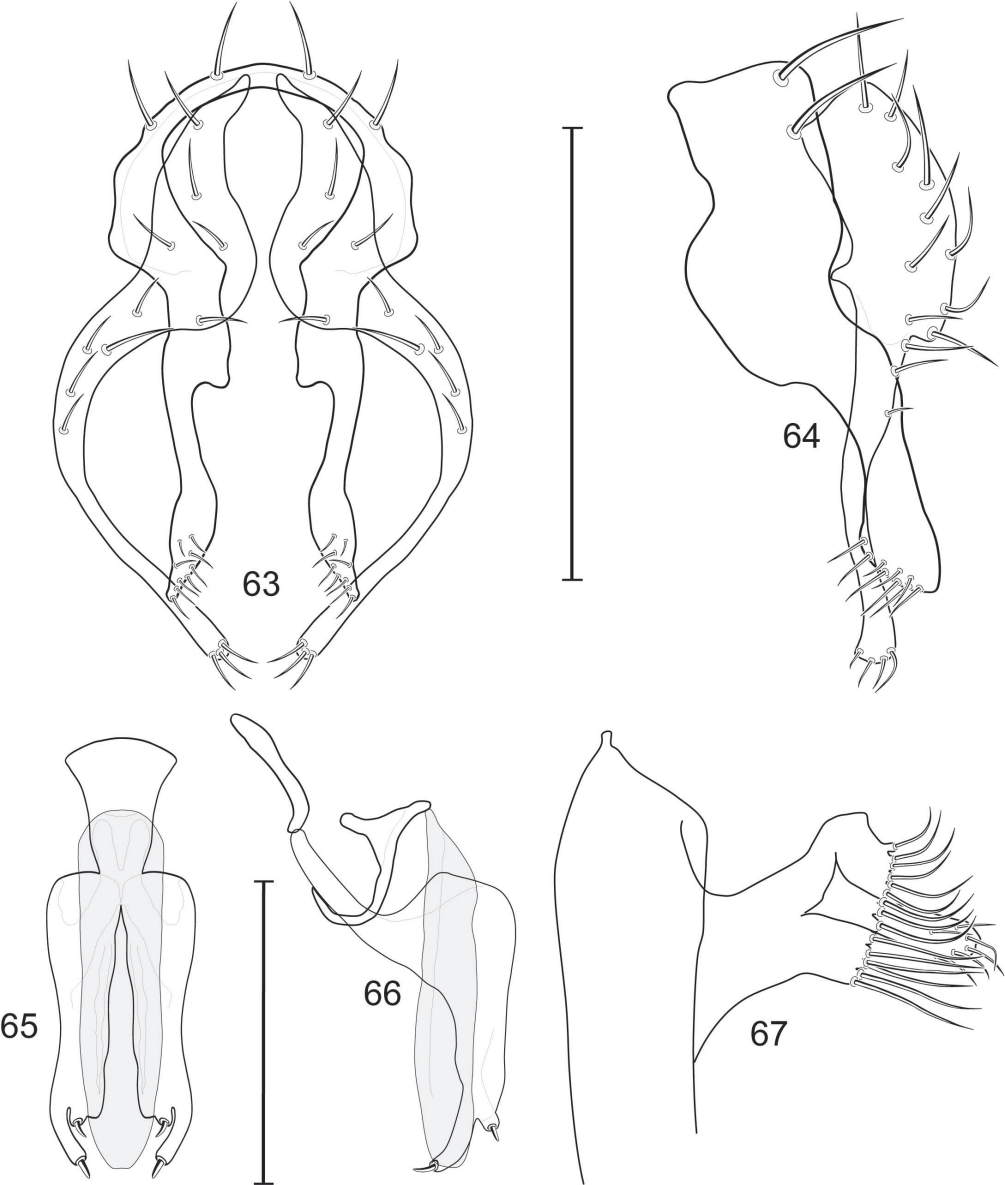
Illustration of *Allotrichoma simplex* (Loew) (male) **63** epandrium, cerci, surstylus, posterior aspect **64** same, lateral aspect **65** aedeagus, phallapodeme, gonite, hypandrium, ventral aspect **66** same, lateral aspect **67** male 5th sternal flap and medial process, lateral aspect. Scale bar = 0.1 mm.

*Abdomen*: Male 5th sternal flap in lateral view (Fig. 67) broadly developed to it apex, bearing approximately 15 apical setulae; 5th medial process in lateral view (Fig. 67) narrow, digitiform. Male terminalia (Figs 63–66): Epandrium in posterior view (Fig. 63) rounded dorsally, constricted laterally at base of surstyli; epandrium in lateral view (Fig. 64) somewhat rectangular dorsally with anterior and posterior margins parallel sided, ventral portion with an anterior bulge and a narrow, sharp incision at about level or base of surstylus; cerci in posterior view (Fig. 63) approximate dorsally then extended laterally to form a lyre-like structure that curves laterally than ventrally and medially, apex of cercus bearing 4 short setulae; cercus in lateral view (Fig. 64) shallowly sinuous, nearly parallel sided, apex slightly expanded, bluntly rounded; surstylus (ventral extensions of epandrium) in posterior view (Fig. 63) as relatively wide at base third, thereafter ventrally abruptly narrowed, especially along medial margin, to form an elongate process that is slightly spatulate apically, apical portion bearing several short setulae; surstylus in lateral view (Fig. 64) moderately narrow, slightly expanding with apex obliquely pointed and bearing several short setulae; aedeagus in ventral view (Fig. 65) elongate, narrowly ovate, very slightly tapered apically, in lateral view (Fig. 66) elongate, nearly parallel sided, apex bluntly rounded; phallapodeme in lateral view (Fig. 66) triangular, keel abruptly tapered to narrow, digitiform apex; gonite in ventral view (Fig. 65) bar-like, slightly wider basally, apex with short, lateral process, apex bearing setula, in lateral view (Fig. 66) wide basally, gently but distinctly arched, tapered to apical point.

#### Type material. 

The lectotype female of *Discocerina simplex* Loew, here designated to preserve stability and make more universal the use of this name, is labeled “[United States.] M[arylan]d./Loew Coll./simplex m[ihi]. [handwritten]/Type 11150 [red; number handwritten]/Prop. of Harvard Univ. [yellow]/LECTOTYPE ♀ *Discocerina simplex* Loew by Mathis & Zatwarnicki 2011 [red].” The lectotype is double mounted (minuten in a block of cork), is in good condition (abdomen removed, dissected, stored in glycerin in an attached microvial), and is deposited in the MCZ (11150).

#### Type locality.

United States. “Maryland.”

#### Other specimens examined from the New World.

CANADA. *ONTARIO*. Fort Erie (42°53.4'N, 78°55.5'W), 18 Apr 1909, M. C. Van Duzee (1♂; CAS).

UNITED STATES. *CONNECTICUT. Fairfield*: Putnam Park (41°20.3'N, 73°22.8'W), 18 Jul 1939, A. L. Melander (1♂; USNM).

*DELAWARE. Kent*: Woodland Beach (39°19.9'N, 75°28.3'W; beach), 18 May 2006, D. and W. N. Mathis (5♂, 3♀; USNM). *Sussex*: Rehoboth (38°43.2'N, 75°04.6'W), 25 Jun 1939, A. L. Melander (1♂; ANSP).

*DISTRICT OF COLUMBIA*. Rock Creek, Milkhouse Ford (38°57.9'N, 77°02.9'W), 18 May 2007, D. and W. N. Mathis (1♀; USU).

*ILLINOIS. McHenry*: Algonquin (42°09.9'N, 88°17.7'W), 1 Jun 1896 (2♂; USNM). *Peoria*: Peoria (40°45'N, 89°36.9'W), 28 Aug 1917, J. M. Aldrich (2♂; USNM). *Washington*: Ashley (38°19.8'N, 89°11.5'W), 7 Aug 1917 (1♂; USNM).

*INDIANA. Porter*: Dunes (near Porter; 41°37'N, 87°04'W), 19 Jul 1933, A. L. Melander (1♂, 1♀; USNM).

*IOWA. Allamakee*: Waterville (43°12.5'N, 91°17.8'W), 2 Aug 1960, D. L. Deonier (1♀; USNM). *Boone*: Fraser Dam (42°07.5'N, 93°58.4'W), 4 Sep 1960, D. L. Deonier (2♂; USNM); Ledges State Park (41°59'N, 93°53.2'W), 19 May–9 Oct 1949, 1955, 1958, 1960, D. L. Deonier, J. L. Laffoon (4♂, 4♀; USNM). *Dickenson*: Gull Point State Park (42°23.3'N, 95°09.9'W), 27 Jun 1960, D. L. Deonier (1♀; USNM). *Hamilton*: Little Wall Lake (42°16.2'N, 93°38.2'W), 13–14 Jul 1960, D. L. Deonier (3♂; USNM)*. Louisa*: Oakville (6.5 km N, 41°09.8'N, 91°08'W), 10 Aug 1980, D. L. Deonier (14♂, 4♀; USNM); Wapello (41°13.9'N, 91°07'W), 9 Aug 1960, D. L. Deonier (3♂, 6♀; USNM). *Monona*: Lewis and Clark State Park (42°02'N, 96°10'W), 6 Jun 1960, D. L. Deonier (1♂, 2♀; USNM). *Story*: Ames (42°02.1'N, 93°37.2'W), 12 May-3 Oct 1959, D. L. Deonier (5♂, 5♀; USNM); Gilbert (6.5 km E; 42°06.5'N, 93°37'W), 25 Jun–22 Jul 1960, 1961, D. L. Deonier (7♂, 4♀; USNM). *Warren*: Banner Mine (41°26.4‘N, 93°33.8‘W), 7 Aug 1960, D. L. Deonier (1♀; USNM).

*MARYLAND. Anne Arundel*: Bristol (3 km WSW; 38°47‘N, 76°40‘W; at Jug Bay), 19 Sep 1988, S. F. Larcher, W. E. Steiner (2♂; USNM). *Baltimore*: Hart-Miller Island (39°15.1‘N, 76°22.4‘W), 31 May 1987, W. E. Steiner, J. M. Swearingen (1♂, 1♀; USNM). *Calvert*: Calvert Beach (38°27.9'N, 76°28.6'W), 17 Sep 1989, J. M. Hill, W. E. Steiner, J. M. Swearingen (1♂; USNM); Chesapeake Beach (38°41.2'N, 76°32.1'W), 18 Aug 1922, J. R. Malloch (1♀; ANSP). *Carroll*: Eldersburg (39°24.2'N, 76°57'W), 2 Jun 1985, J. E. Lowry, W. E. Steiner (1♂; USNM). *Garrett*: Broadford Lake (39°24.7'N, 79°22.4'W; 750 m), 24 Aug 2006, D. and W. N. Mathis (23♂, 2♀; USNM). *Montgomery*: Clarksburg, Little Bennett Regional Park (39°16'N, 77°16.8'W), 21 Sep 1990, M. J. and R. Molineaux, W. E. Steiner (1♂; USNM); Kensington (39°01'N, 77°04.1'W), 13 Apr 2006, D. and W. N. Mathis (1♂; USNM); Little Bennett Regional Park (39°16'N, 77°16.8'W), 20 Apr 2005, D. and W. N. Mathis (1♂; USNM); Plummers Island (38°58.2'N, 77°10.6'W), 12 Apr 1914, W. L. McAtee (1♂, 1♀; ANSP). *Prince George's*: (38°50'N, 76°51'W), 4 Jul 1954, C. W. Sabrosky (1♂; USNM). *Talbot*: Wittman (38°47.6'N, 76°17.6'W), 19 May 1986, W. E. Steiner (1♀; USNM).

*MASSACHUSETTS. Barnstable*: Woods Hole (41°31.5'N, 70°40.3'W), 24 Aug 1922, A. H. Sturtevant (1♂; USNM).

*MICHIGAN. Monroe*: Monroe (41°55'N, 83°23.9'W), 2 Jul 1939, G. C. Steyskal (1♂, 4♀; USNM). *Newaygo*: (43°25.2'N, 85°48'W), 27 Jun 1953, R. R. Dreisbach (1♂; USNM). *Wayne*: Detroit (42°23'N, 83°06.1'W), 17 Sep 1939, G. C. Steyskal (1♂; USNM); Grosse Ile (42°07.7'N, 83°08.7'W), 8 Aug 1954, G. C. Steyskal (2♂; USNM).

*MINNESOTA. Becker*: Detroit Lake (46°49'N, 95°50.7'W), 9 Aug 1935, A. L. Melander (2♂; USNM). *Crow Wing*: Pequot Lakes (8 km SE; 46°36'N, 84°17'W), 6 Jul 1957, J. L. Laffoon (1♂; USNM). *Goodhue*: Lake Pepin (44°26'N, 92°10.5'W), 31 May 1951, R. Namba (2♂; USNM). *Lake*: Basswood Lake (48°04.5'N, 91°34.5'W), 17 Aug 1950, R. Namba (1♂, 2♀; USNM). *Mille Lacs*: Mille Lacs Lake (46°14'N, 93°39'W), 2 Jun 1937, D. G. Denning (1♂; USNM).

*NEW JERSEY. Burlington*: Crowley's Landing (39°37.6N, 74°37.2'W), 17 Jun 2004, D. and W. N. Mathis (3♂, 1♀; USNM). *Ocean*: Tuckerton (39°36.2'N, 74°20.8'W), 17 Jun 2004, D. and W. N. Mathis (19♂, 6♀; USNM).

*NEW YORK. Niagara*: Niagara Falls (43°05.7'N, 79°03.4'W), 26 Mar 1911, M. C. Van Duzee (2♂; CAS). *Tompkins*: Inlet Valley (42°24.6'N, 76°32.3'W), 15 May 1956, B. A. Foote (1♂; USNM). *Wyoming*: Portageville (42°34.2'N, 78°02.4'W), 13 Jun 1963, W. W. Wirth (1♂; USNM).

*OHIO. Ashtabula*: Geneva State Park (41°51.3'N, 80°58.3'W), 16 Apr 1976, D. L. Deonier (1♂; USNM). *Auglaize*: St. Marys Lake (= Grand Lake; 40°31.3'N, 84°25.3'W; sedge meadow), 26 May 1977, J. T. Runyan, B. A. Steinly (10♂, 4♀; USNM). *Brown*: Grant Lake (38°47.3'N, 83°54.9'W), 7 Jul 1975, J. Regensburg (1♂, 1♀; USNM). *Butler*: Hueston Woods State Park (Acton Lake; 39°34.3'N, 84°44.5'W), 16 Jul 1974, J. Regensburg (12♂, 15♀; USNM); Oxford, Peffer Park (39°29.7'N, 84°43.9'W), 1 Jul 1975, J. Regensburg (1♂; USNM). *Champaign*: Kiser Lake State Park (40°11.5'N, 83°58'W), 5 Aug 1976, B. A. Steinly (4♂, 7♀; USNM). *Clermont*: Stonelick State Park (39°13.6'N, 84°03.5'W), 1 Aug 1974, J. Regensburg (6♂, 5♀; USNM). *Delaware*: Delaware Wildlife Refuge (40°24.5'N, 83°02.7'W), 28 Jun 1977, B. A. Steinly (3♂, 7♀; USNM). *Fulton*: Harrison Lake (41°38.6'N, 84°22.3'W), 21 Aug 1977, B. A. Steinly (2♂, 5♀; USNM). *Hamilton*: Whitewater River (39°14.1'N, 84°47.1'W), 5 Aug 1974, J. Regensburg (2♀; USNM). *Hocking*: Hocking Lake (39°30.5'N, 82°27.2'W), 8 Nov 1974, J. Regensburg (3♂, 3♀; USNM). *Lake*: Mentor marsh (41°40'N, 81°20.3'W), 16 Jun 1976, D. L. Deonier (1♂; USNM). *Lawrence*: Vesuvius Lake (38°34.8'N, 82°37.5'W), 23 Aug 1974, J. Regensburg (1♀; USNM). *Lorain*: Amherst, Beaver Creek (40°29.3'N, 84°32.9'W), 22–24 Aug 1977, B. A. Steinly (10♂, 5♀; USNM); Vermillion River, Mill Hollow (41°22.9'N, 82°19'W), 22 Sep 1976, B. A. Steinly (1♂; USNM). *Lucas*: Presque Isle (41°41.7'N, 83°27.4'W), 6 May 1977, B. A. Steinly (2♂, 3♀; USNM). *Madison*: Madison Lake State Park (39°30.9'N, 83°21.3'W), 22 Jul 1974, J. Regensburg (1♂, 1♀; USNM). *Meigs*: Belleville Dam (39°05.7'N, 81°44.9'W), 20 Aug 1974, J. Regensburg (9♂, 14♀; USNM); Fork Run State Park (39°03.9'N, 81°46.3'W), 27 Aug 1974, J. Regensburg (4♂, 2♀; USNM). *Mercer*: Montezuma (40°29.3'N, 84°32.9'W), 26 May 1977, B. A. Steinly (10♂, 7♀; USNM); Montezuma Creek (40°29.6'N, 84°33'W), 11 Oct 1976, B. A. Steinly (2♂, 4♀; USNM); Windy Point (40°31'N, 84°32.2'W), 26 May 1977, B. A. Steinly (14♂, 9♀; USNM). *Morgan*: Burr Oak State Park (39°33.3'N, 82°02.8'W), 26 Aug 1974, J. Regensburg (5♂, 2♀; USNM); Muskingum River near Big Bottom State Memorial (39°31.1'N, 81°47.5'W), 26 Aug 1974, J. Regensburg (1♂, 2♀; USNM). *Ottawa*: South Bass Island (41°38.6'N, 82°50.3'W), 22 Jul-22 Aug 1977, B. A. Steinly (6♂, 5♀; USNM). *Portage*: Kent (41°09.2'N, 81°21.5'W), 25 Aug 1969, J. Scheiring (1♂; USNM); Kent (1.6 km E; 41°09.2'N, 81°21'W; dead snail), 2–11 Sep 1972, B. A. Foote (5♂, 1♀; USNM); Kent (5 km E; 41°09.2'N, 81°20'W; muskrat dung), 10 Sep 1975, B. A. Foote (1♂, 3♀; USNM); Ravenna (5 km E; 41°09.5'N, 81°13'W), 3 May 1969, J. Scheiring (3♂, 5♀; USNM). *Preble*: Four-Mile Creek, Route 725 (39°35'N, 84°45.8'W), 2 Jun-26 Sep 1978, 1979, B. A. Steinly (14♂, 13♀; USNM). *Stark*: Berlin Reservoir (40°58.9‘N, 81°06‘W), 7 Jul 1976, B. A. Steinly (1♂, 3♀; USNM).

*TEXAS. Brewster*: Big Bend National Park, Boquillas Canyon (29°12.7'N, 102°53.2'W), 20 Jun 1953, W. W. Wirth (1♂, 1♀; USNM).

*VERMONT. Chittenden*: Burlington (44°28.5'N, 73°12.7'W), 1 Sep 1957, A. L. Melander (6♂, 10♀; USNM).

*VIRGINIA. Accomack*: Assateague Island, near Refuge headquarters (37°54.5'N, 75°21.6'W), 3 Oct 2005, D. and W. N. Mathis (2♂, 1♀; USNM). *Arlington*: 4-Mile Run (38°50.4'N, 77°02.7'W), 28 May 1977, W. N. Mathis (2♂, 3♀; USNM). *Culpeper*: Lake Pelham (38°27.8'N, 78°02.7'W), 28 Apr 2006, D. and W. N. Mathis (4♂, 2♀; USNM). *Fairfax*: Annandale (38°49.8'N, 77°11.8'W), 28 Jul 1968, J. W. Adams (1♂; USNM); Great Falls (Clay Pond; 39°00.1'N, 77°15.4'W), 13 Jun-17 Aug 2006, W. N. Mathis, T. Zatwarnicki (11♂, 2♀; USNM); Great Falls (Patowmack Canal; 39°00.1'N, 77°15.2'W), 5 Jul 2007, D. and W. N. Mathis (1♂; USNM); Great Falls (Potomac River; 39°0.2'N, 77°15.2'W), 4 Oct 2007, D. and W. N. Mathis (1♂; USNM); Great Falls (quarry; 38°59.1'N, 77°14.8'W; 50 m), 13 Jun 2007, D. and W. N. Mathis (5♂; USNM); Great Falls (river trail; Potomac River; 38°59.1'N, 77°14.6'W; 60 m), 3 Apr 2007, D. and W. N. Mathis (1♂, 1♀; USNM); Scott Run (38°58.1'N, 77°12.2'W), 7 Jun, 1955, C. W. Sabrosky (2♀; USNM); Turkey Run (mouth; 38°57.9'N, 77°09.4'W), 22 May-7 Sep 2006, 2007, W. N. Mathis, T. Zatwarnicki (65♂, 9♀; USNM). *Giles*: Mountain Lake Bio Station (37°22.5'N, 80°31.3'W; 1176 m), 23 Sep 2007, D. and W. N. Mathis (1♂; USNM). *Grayson*: Galax (15 km S; Chestnut Creek; 36°34.9'N, 80°53.5'W), 28 Jun 1986, W. E. Steiner (1♂; USNM). *Henry*: Martinsville Reservoir (36°44.7'N, 79°52.2'W), 17 May 2005, D. and W. N. Mathis (13♂, 1♀; USNM). *Loudoun*: Lucketts (6 km N, 39°16.3'N, 77°32.9'W), 15 Apr 2005, D. and W. N. Mathis (1♂; USNM). *Louisa*: Lake Anna (38°05.8'N, 77°53.6'W; Christopher Run Campground), 4 May 2007, D. and W. N. Mathis (1♀; USNM). *Patrick*: Woolwine (36°47.4'N, 80°16.7'W; 300 m), 17 May 2005, D. and W. N. Mathis (4♂, 1♀; USNM). *Rappahannock*: Hazel River (NW Culpeper; 38°33.8'N, 78°11.6'W, 171 m), 28 Jun 2008, W. N. Mathis and T. Zatwarnicki (1♂; USNM). *Roanoke*: Salem (Roanoke River; 37°16.1'N, 80°02.2'W; 300 m), 23 Sep 2007, D. and W. N. Mathis (5♂, 1♀; USNM). *Smyth*: Saltville (36°52.3'N, 81°46.4'W, 1710 ft), 22 Jun 2007, D. and W. N. Mathis (1♀; USNM). *Spotsylvania*: Rappahannock River (38°18.8'N, 77°32.5'W), 15 Apr-10 Oct 2006, 2007, D. and W. N. Mathis (21♂, 7♀; USNM). *Stafford*: Aquia Harbour (38°27.9'N, 77°23.3'W), 15 May 2000, D. and W. N. Mathis (2♂; USNM); Aquia Harbour, Aquia Creek (38°27.8'N, 77°23.1'W), 12 Apr-2 Sep 2006, D. and W. N. Mathis (2♂, 1♀; USNM); Aquia Harbour, Lions Park (38°27'N, 77°23.3'W), 10 Mar-18 Dec 2004, 2005, 2006, 2007, D. and W. N. Mathis (27♂, 14♀; USNM); Aquia Landing (38°23.2'N, 77°19'W), 7 May-8 Sep 2003, 2005, D. and W. N. Mathis (12♂, 4♀; USNM); Curtis Park Lake (38°26'N, 77°33.3'W), 30 Mar-2 Jul 2005, 2007, D. Mathis (16♂, 4♀; USNM); Falmouth (38°19.2'N, 77°28.1'W; Rappahannock River; 9 m), 11 Apr-30 Jun 2007, 2008, D. and W. N. Mathis (23♂, 2♀; USNM). *York*: Maury Lake (ca. James River; 37°02.5'N, 76°29.2'W), 22 Mar-19 Aug 2006, 2007, D. and W. N. Mathis (1♂, 1♀; USNM). *Westmoreland*: Potomac Beach (38°16.4'N, 76°59.6'W; Potomac River), 8 Jun 2007, D. and W. N. Mathis (1♀; USNM). *Independent City*: Fredericksburg (Rappahannock River; 38°18.3'N, 77°27.5'W), 14 Apr 2006, D. and W. N. Mathis (1♀; USNM).

*WEST VIRGINIA. Cavell*: Culloden (38°25.2'N, 82°03.3'W), Jul 1954, M. R. Wheeler (1♂; USNM). *Hardy*: Baker (39°02.5'N, 78°44.9'W; 405 m), 20 Jun-13 Jul 2007, D. and W. N. Mathis (13♂; USNM); Lost City (1.6 km N, 38°56.5'N, 78°48.9'W; 460 m), 12 Jul 2007, D. and W. N. Mathis (1♀; USNM); Mathias (38°52.6'N, 78°52'W; 465 m), 13 Jul 2007, D. and W. N. Mathis (1♂; USNM); Trout Pond (38°57.4'N, 78°44.2'W; 595 m), 13 Jul 2007, D. and W. N. Mathis (1♂; USNM). *Mercer*: Ceres (Kee Reservoir; 37°18.4'N, 81°10.4'W; 757 m), 24 Sep 2007, D. and W. N. Mathis (5♂; USNM). *Morgan*: Great Cacapon (39°37.2'N, 78°17.6'W), 3 Jul 1977, W. W. Wirth (1♂; USNM). *Perkins*: Silver Lake (39°15.3'N, 79°29.8'W; 762 m), 21 Jun 2007, D. and W. N. Mathis (3♂; USNM). *Pocahontas*: Marlinton (Greenbrier River; 38°13.5'N, 80°05.7'W), 21 Jun 2007, D. and W. N. Mathis (3♂; USNM); Marlinton, Knapp Creek (38°13'N, 80°06'W), 29 Jun 1982, W. N. Mathis (2♀; USNM). *Richie*: North Bend State Park (39°13.4'N, 81°06.6'W), 23 Jun 1970, G. C. Steyskal (1♂♀; USNM). *Roanoke*: Salem (Roanoke River; 37°16.1'N, 80°02.2'W; 300 m), 23 Sep 2007, D. and W. N. Mathis (4♂, 1♀; USNM). *Summers*: Hinton (37°41.8'N, 80°53'W; New River; 427 m), 26 Sep 2007, D. and W. N. Mathis (1♂, 2♀; USNM).

#### Distribution.

**Figure 68.**
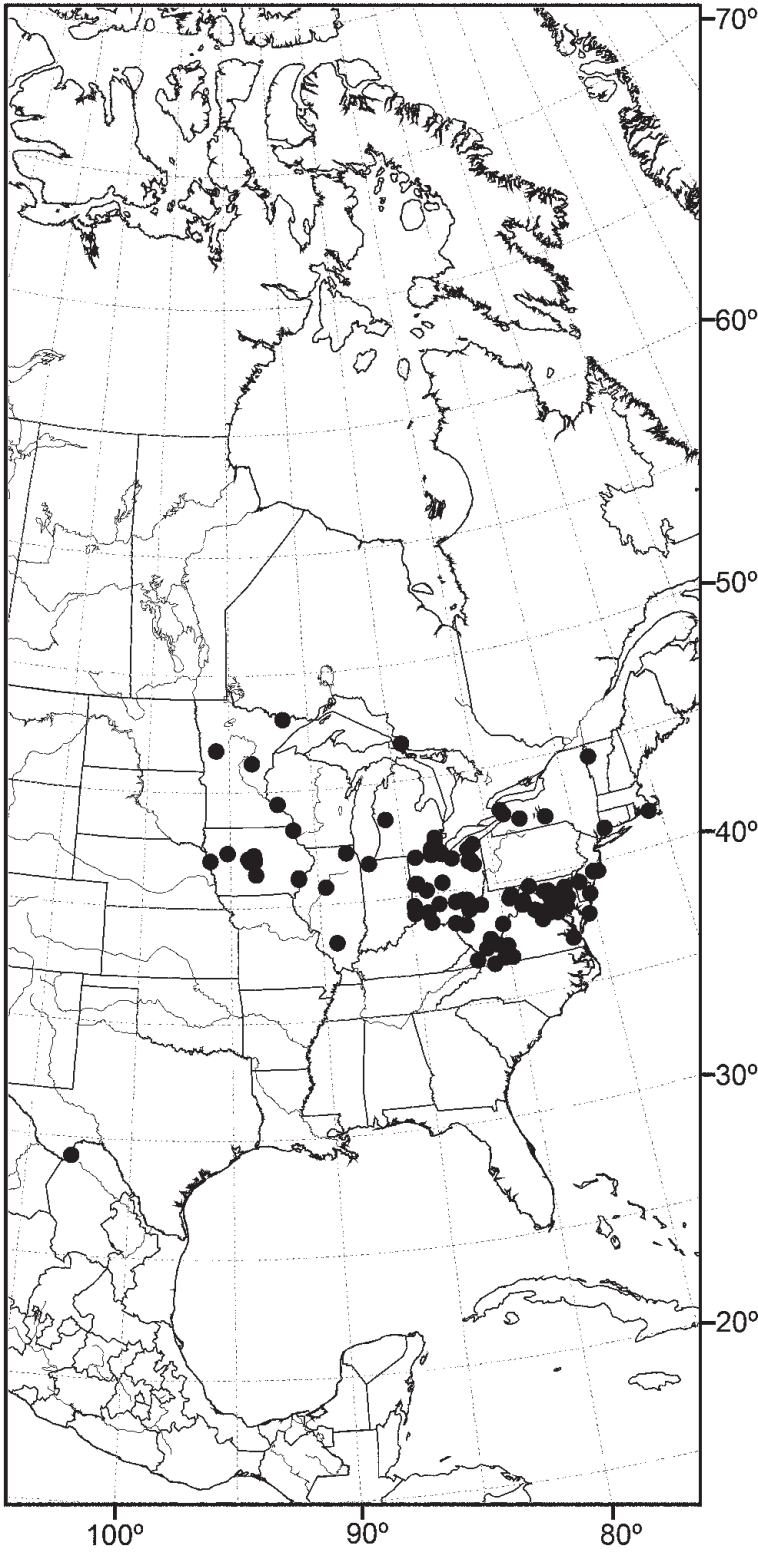
Distribution of *Allotrichoma simplex* (Loew).

(Fig. 68) *Nearctic*: Canada (Ontario), United States (Connecticut, Delaware, District of Columbia, Illinois, Indiana, Iowa, Maryland, Massachusetts, Michigan, Minnesota, New Jersey, New York, Ohio, Texas, Vermont, Virginia, West Virginia). *Palearctic*: Austria, Bulgaria, China (Tibet), Czech Republic, France, Hungary, Israel, Italy, Morocco, Poland, Spain, Russia (European Territory), Switzerland, Yugoslavia.

#### Natural history.

Deonier (1965: 501) found this species to be “occasional” in mud shore and sand shore habitats but rare in sedge meadow and *Eragrostis* mat habitats. Runyan and Deonier (1979) produced the only illustrations of immature stages, including an egg, the cephalopharyngeal skeleton of the third instar larva, and the puparium.

#### Remarks.

This species has a Holarctic distribution, and Palearctic specimens have been named at least three different times, with *Allotrichoma simplex* being the senior synonym. The junior synonyms in chronological order are: *Allotrichoma filiforme* Becker (1896), *Allotrichoma trispinum* Becker (1896), and *Allotrichoma dahli* Beschovski (1966a). In Europe, there is also a very similar species, *Allotrichoma strandi* Duda, that differs only slightly but apparently consistently from this species. In males of *Allotrichoma simplex*, the cercus has four apical setulae, compared to three in *Allotrichoma strandi*, and the mesonotal stripes are more evident in *Allotrichoma strandi* than in *Allotrichoma simplex*.

In the Nearctic Region, this is a common species that may have the most widespread distribution.

### Allotrichoma (Allotrichoma) wallowa sp. n.

urn:lsid:zoobank.org:act:AAB69BB1-D8DC-45EE-8EDA-05A1CDEFE80A

http://species-id.net/wiki/Allotrichoma_wallowa

Figs 69 74

#### Description.

This species is distinguished from congeners by the following combination of characters: Small shore flies, body length 1.30–1.95 mm. *Head*: Medial facial carina above facial prominence shallow; labella broad, fleshy, shorter than mediproboscis; clypeus microtomentose, usually gray.

*Thorax*: Presutural supra-alar seta present. Pleural area lacking stripes. Wing with costal vein ratio 0.32–0.38; M vein ratio 0.43–0.50. Legs blackish brown to black; midtibia bearing numerous, erect setae along dorsal surface.

**Figures 69–73.**
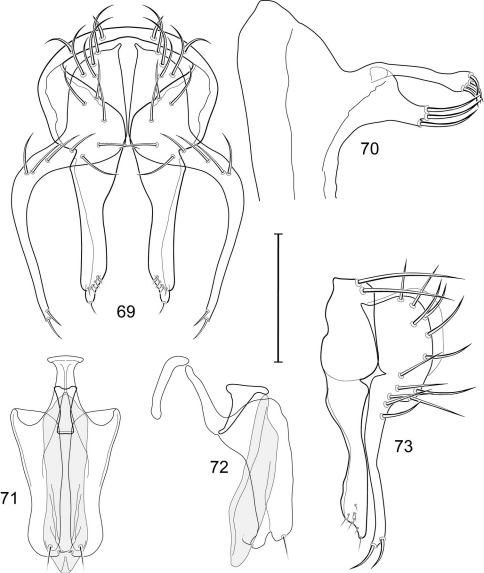
Illustration of *Allotrichoma wallowa* sp. n. (male) **69** epandrium, cerci, surstylus, posterior aspect **70** male 5th sternal flap and medial process, lateral aspect **71** aedeagus, phallapodeme, gonite, hypandrium, ventral aspect **72** same, lateral aspect **73** epandrium, cerci, surstylus, lateral aspect. Scale bar = 0.1 mm.

*Abdomen*: Male 5th sternal flap in lateral view (Fig. 70) moderately elongate, bearing 3 long setulae apically; 5th medial process in lateral view (Fig. 70) very elongate, nearly straight, apex widened, bearing 4–5 apical setulae. Male terminalia (Figs 69, 71–73): Epandrium in posterior view (Fig. 69) rounded dorsally, constricted laterally at base of surstyli; epandrium in lateral view (Fig. 73) somewhat rectangular dorsally with anterior and posterior margins parallel sided, ventral portion with an anterior bulge and a narrow, sharp incision at about level of base of surstylus; cerci in posterior view (Fig. 69) approximate dorsally then extended laterally to form a lyre-like structure that curves laterally than ventrally, apex of cercus bearing 2 short setulae; cercus in lateral view (Fig. 73) shallowly sinuous, nearly parallel sided, apex not expanded, bluntly rounded; surstyli (ventral extensions of epandrium) in posterior view as relatively wide at base third, thereafter ventrally abruptly narrowed, especially along medial margin, to form an elongate process that is slightly spatulate apically, apical portion bearing several short setulae; surstylus in lateral view (Fig. 73) moderately narrow, slightly expanding with apex obliquely pointed, anteroventral surface of apex shallowly emarginate, bearing several, very short setulae; aedeagus in ventral view (Fig. 71) elongate, narrowly ovate, very slightly tapered apically, in lateral view (Fig. 72) elongate, nearly parallel sided, apex bluntly rounded; phallapodeme in lateral view (Fig. 72) triangular, keel abruptly tapered to narrow, digitiform apex; gonite in ventral view (Fig. 71) almost bar-like, wider basally, apex broadly rounded, with short, medial process, apex bearing setula, in lateral view (Fig. 72) only slightly wider basally, nearly straight, slightly tapered to blunt apex that is medially notched, producing a shallowly bifurcate apex, anterior and posterior process of shallow bifurcation bearing a single setula.

#### Type material.

The holotype male is labeled “**USA. OREGON.** Baker: Goose Creek [35 km E Baker City] (44°49.2'N, 117°27.79'W; 825 m), 7Jun2006, D. & W. N. Mathis/HOLOTYPE ♂ *Allotrichoma wallowa* W. Mathis & T. Zatwarnicki USNM [red]/USNM ENT 00117961 [plastic bar code label].” The holotype male is double mounted (minuten in a block of plastic elastomer), is in excellent condition, and is deposited in the USNM. Thirty-seven paratypes (32♂, 5♀; USNM) bear the same locality label as the holotype. Other paratypes are as follows: *OREGON. Harney*: Page Springs Campground, Blitzen River (42°47'N, 118°51'W; 1300 m), 6 Aug 2005, D. and W. N. Mathis (49♂, 7♀; USNM); Pikes Creek (42°34.5'N, 118°31.7'W; 1320 m), 7 Aug 2005, D. and W. N. Mathis (3♂, 4♀; USNM).

#### Type locality.

United States. Oregon Baker: Goose Creek (35 km E Baker City; 44°49.2'N, 117°27.79'W; 825 m).

#### Other specimens examined from the New World.

CANADA. *ALBERTA*. Okotoks, Sheep River (50°44.4'N, 113°51.3'W), 27 Jun 1968, W. W. Wirth (2♂; USNM).

MEXICO. *BAJA CALIFORNIA*. Sierra San Pedro Martir (La Grulla; 31°38'N, 116°26'W; 2100 m), 12 Jun 1953, P. H. Arnaud, Jr. (1♂, 2♀; USNM).

*OAXACA*. Oaxaca (16°58'N, 96°44'W), 2 May 1965, H. V. Weems (1♂, 1♀; USNM).

UNITED STATES. *ARIZONA. Maricopa*: Tempe (33°24.9'N, 111°54.6'W), 11 May 1942, A. L. Melander (2♂; USNM). *Pima*: Sabino Canyon (32°18.6'N, 110°49.3'W), 5 May 1942, A. L. Melander (2♂; USNM). *Santa Cruz*: Nogales (31°20.4'N, 110°56'W), 28 Jun 1953, W. W. Wirth (1♂, 1♀; USNM).

*CALIFORNIA. Calaveras*: Milton (38°01.9'N, 120°51.1'W), 21 Oct 1917, J. C. Bradley (5♂; USNM). *Del Norte*: Panther Flat, Middle Fork Smith River (41°50.6'N, 123°56'W), 3 Jun 2009, D. and W. N. Mathis (3♂; USNM). *Humboldt*: Grizzly Creek Redwoods State Park (40°29'N, 123°54.4'W), 11 Aug 1953, P. H. Arnaud, Jr. (1♂; USNM). *Imperial*: Brawley (32°58.7'N, 115°31.8'W), 7 Apr 1959, E. I. Schlinger (2♂, 1♀; USNM). *Kern*: Onyx (35°41.4'N, 118°13'W), 23 Jul 1939, D. E. Hardy (2♂, 1♀; USNM). *Los Angeles*: Littlerock (37°31.3'N, 117°59'W), 30 Mar 1953, A. H. Sturtevant (1♂; USNM); Palmdale (34°34.8'N, 118°07'W), 30 Sep 1953, A. H. Sturtevant (1♂; USNM); Saugus (34°24.7'N, 118°32.4'W), 10 Apr 1953, A. H. Sturtevant (1♂; USNM). *Lake*: Clear Lake (39°03.7'N, 122°49.6'W), 18 Jun–11 Oct 1935, 1947, A. L. Melander, W. W. Wirth (4♂, 7♀; USNM). *Mendocino*: Russian River near Hopland (32°10.4'N, 116°51.8'W), 4 May 1968, W. J. Turner (11♂, 9♀; USNM, WSU). *Mono*: Mammoth lakes (37°36.3'N, 119°0.7'W), 29 Jul 1940, L. J. Lipovsky (1♂; USNM); Mono Lake (S shore; 38°01'N, 119°03.6'W), 12 Aug 1980, R. S. Zack (2♂; WSU). *Napa*: Monticello Dam (38°30.8'N, 122°06.2'W), 3 Nov 1962, M. E. Irwin (1♂; USNM). *Orange*: Silverado Canyon (33°45.4'N, 117°40.6'W), 12 Oct 1963, M. E. Irwin (2♂; USNM). *Riverside*: Agua Caliente (33°49.4'N, 116°32.7'W), 25 Feb 1970, P. H. Arnaud, Jr. (2♂; USNM); Andreas Canyon (33°45.7'N, 116°32.2'W), 25 Feb 1954, A. L. Melander (1♂; USNM); Cathedral Canyon (33°46.8'N, 116°27.9'W), 3 Apr 1945, A. L. Melander (1♂; USNM); Magnesium Spring Canyon (near Indio; 33°43.2'N, 116°13'W), 5 Apr 1945, A. L. Melander (1♂; USNM); Morongo Valley (33°57.6'N, 116°31.5'W), 26 Sep 1944, A. L. Melander (1♂; USNM); Riverside (33°58.8'N, 117°22.4'W), 24 May 1950, A. L. Melander (1♂; USNM); Temecula (33°29.6'N, 117°08.9'W), 25 May 1975, T. W. Fisher (1♂, 1♀; USNM); Thousand Palms (33°48.7'N, 116°22.6'W), 18 Mar 1964, P. Rauch (1♂; USNM). *Sacramento*: Isleton (38°09.7'N, 121°36.7'W), 15 Oct 1967, M. S. Wasbauer (1♀; USNM). *San Bernardino*: Barton Store (32°10.4'N, 116°51.8'W), 23 Aug 1952, A. L. Melander (1♀; USNM); Chino (33°59'N, 117°39.9'W), 6 Nov 1928, A. H. Sturtevant (1♀; USNM); Jenks Lake (34°09.9'N, 116°52.9'W), 7 Sep 1950, A. L. Melander (1♂; USNM); Saratoga Spring (35°40.9'N, 116°25.3'W; 100 m), 31 Mar 2005, D and W.N. Mathis (2♂, 7♀; USNM); Victorville (34°32.2'N, 117°17.5'W), 10 May 1952, A. H. Sturtevant (2♂; USNM). *San Diego*: Borrego (33°13.2'N, 116°20.1'W), 2 May 1945, A. L. Melander (1♂; USNM); El Cajon (32°47.7'N, 116°57.8'W), 8 Aug 1944, A. L. Melander (2♂, 1♀; USNM); El Capitan (32°52.1'N, 116°55'W), 25 Sep 1955, P. H. Arnaud, Jr. (10♂, 6♀; USNM); Gavilan Hills (33°47'N, 117°22.1'W), 1 Oct 1952, A. L. Melander (3♀; USNM); Pala (33°21.8'N, 117°04.5'W), 6 Jun 1945, A. L. Melander (1♂, 1♀; USNM); Palm Canyon (33°13.2'N, 116°20.1'W), 27 Feb 1935, A. L. Melander (1♂, 1♀; USNM). *Shasta*: Shasta Lake (40°40.8'N, 122°22.3'W), 8 Apr 1959, M. T. James (3♂, 4♀; USNM). *Ventura*: Santa Paula (34°21.3'N, 119°03.6'W), 8 May 1952, A. H. Sturtevant (1♂; USNM). *Yolo*: Davis (38°32.7'N, 121°44.1'W), 11 Jul 1953, E. I. Schlinger (1♂; USNM); Elkhorn Ferry (38°42.5'N, 121°37.1'W), 16 Apr 1952, E. I. Schlinger (1♂; USNM).

*COLORADO. Montezuma*: McPhee Reservoir (37°29.7'N, 108°33'W; 2115 m), 3 Aug 2007, D. and W. N. Mathis (1♂, 2♀; USNM).

*IDAHO. Idaho*: Little Salmon River (42°25'N, 116°18.9'W), 26 Jun 1986, R. S. and V. L. Zack (2♂, 1♀; WSU). *Kootenai*: Fernan lake (47°40.4'N, 116°44.7'W), 15 Jul 1968, W. N. Mathis (1♂, 1♀; USNM). *Latah*: Potlatch River, 1.6 km N Kendrick; 46°55.3'N, 116°53.9'W), 8 Jun 1989, R. S. Zack (5♂, 4♀; WSU); Strychnine Creek (NE Harvard; 46°56.6'N, 116°38.4.7'W), 29 Apr-14 Jul 1980, 1981, R. S. Zack (2♂, 1♀; WSU). *Nez Pierce*: Hatwai Creek (8 km E Lewiston; 46°26'N, 116°54.8'W), 31 Jul 1984, W. J. Turner (1♂; WSU)*. Oneida*: Curlew Valley Reservoir (42°04.5'N, 112°32.5'W), G. F. Knowlton (1♂, 2♀; USNM).

*MINNESOTA. Le Suer*: Ottawa (44°22.9'N, 93°56.8'W), 18–19 Jul 1922, W. E. Hoffmann (1♂; USNM).

*MONTANA. Gallatin*: Three Forks (43°53.6'N, 111°33.1'W), 1 Aug 1948, A. L. Melander (1♂; USNM).

*NEVADA. Clark*: Henderson (36°2.3'N, 114°58.9'W), 15 May 2004, D. Mathis (1♂, 4♀; USNM). Las Vegas Wash (36°05.2'N, 114°58.8'W), 11 Apr-11 May 2001, 2005, D. and W. N. Mathis (14♂, 6♀; USNM); Red Rock Canyon (36°09.8'N, 115°31.1'W), 12 May 2001, D. and W. N. Mathis (1♂; USNM). *Nye*: Ash Meadows National Wildlife Refuge (36°26.7'N, 116°21'W), 12 May 2001, D. Mathis (6♂, 3♀; USNM).

*NEW MEXICO. Catron*: Gila River (33°13.6'N, 106°15.1'W; 1750 m), 15 Aug 2007, D. and W. N. Mathis (7♂; USNM). *Cibola*: Bluewater Lake State Park (35°18.1'N, 108°06.6'W; 2220 m), 9 Aug 2007, D. and W. N. Mathis (3♂; USNM). *Colfax*: Cimarron (36°30.6'N, 104°55'W), 26 May 1969, W. W. Wirth (1♂, 3♀; USNM). *Grant*: Mimbres River (NM Hwy. 61 and Royal John Mine Road; 32°43.8'N, 107°52'W; 1665 m), 2–22 Aug 2007, 2008, 2009, D. and W. N. Mathis (54♂, 1♀; USNM); Mimbres River (NM Hwy. 35; 19 km N NM Hwy 152; 32°56.9'N, 108°01.1'W; 1912 m), 2 Aug 2008, D. and W. N. Mathis (17♂; USNM); Silver City (Big Ditch; 32°46.4'N, 108°16.5'W; 1790 m), 14 Aug 2007, D. and W. N. Mathis (1♂; USNM). *San Juan*: Navajo Dam (San Juan River; 36°48.4'N, 107°36.8'W; 1743 m), 4 Aug 2007, D. and W. N. Mathis (1♂; USNM). *Sandoval*: La Cueva (Junction of Highways 126 and 4; 35°52'N, 106°38.4'W; 2342 m), 7 Aug 2007, D. and W. N. Mathis (1♂; USNM). *Socorro*: Socorro (34°04.3'N, 106°54.4'W), S. W. Williston (1♂; USNM).

*OREGON. Benton*: Cary's Grove (44°22.6'N, 123°36.1'W), 2 Sep 1974, W. N. Mathis (2♂, 3♀; USNM). *Douglas*: North Umpqua River (42°43.2'N, 122°60'W; 250 m), 31 Jul 2005, D. and W. N. Mathis (12♂, 9♀; USNM). *Harney*: (43°10'N, 119°0.1'W), 29 Jun 1953, A. B. Gurney (1♂, 1♀; USNM); Mickey Springs (42°40.6'N, 118°20.7'W; 1235 m), 12 Sep 1981, R. S. Zack (1♂; WSU); Page Springs Campground, Blitzen River (42°47'N, 118°51'W; 1300 m), 6 Aug 2005, D. and W. N. Mathis (6♂, 4♀; USNM); Steens Mountain, Fish Lake Campground (42°44.3'N, 118°38.7'W; 2250 m), 6 Aug 2005, D. and W. N. Mathis (1♂; USNM). *Josephine*: Grants Pass (42°26.3'N, 123°19.7'W), 4 Aug 1952, H. A. Scullen (4♂, 2♀; OSU). *Klamath*: Klamath River (12 km W Keno; 42°07.6'N, 121°56'W), 27 Jul 1988, R. Danielsson (1♂; ZIL); Odell Lake (43°34.4'N, 121°60'W), 26 Jul 1988, R. Danielsson (1♂; ZIL). *Lake*: Lakeview (43.5 km E; Drake Creek; 42°11'N, 119°59.3'W), 29 Jul 2005, D. and W. N. Mathis (2♂; USNM). *Malheur*: Nyssa (43°52.6'N, 116°59.7'W), 16 Jul 1940, H. A. Scullen (1♂; OSU). *Union*: Ladd Canyon (45°12.8'N, 118°01.6'W; 1295 m), 30 Jul 1977, E. J. Davis, R. S. Zack (1♂; WSU).

*SOUTH DAKOTA. Mellette*: Little White River (43°43.7'N, 100°39.6'W), 4 Jun 1969, W. W. Wirth (2♂, 3♀; USNM).

*TEXAS. Blanco*: Miller Creek (30°15.2'N, 98°31.7'W; 410 m), 3 Jun 2004, W. N. Mathis (16♂, 3♀; USNM); Twin Sisters (30°00.2'N, 98°24.3'W), 25 May 1918, J. C. Bradley (1♂; USNM). *Kimble*: Junction (30°29.4'N, 99°46.3'W), 4 Jul 1986, R. S. Zack (1♂; WSU); Junction (South Llano River; 30°29.5'N, 99°45.4'W), 4–6 Jul 1986, R. S. Zack (10♂, 13♀; WSU); Junction (South Llano River; 30°29.6'N, 99°45.1'W; 510 m), 4 Jun 2004, W. N. Mathis (6♂, 1♀; USNM). *Kinney*: Del Rio (30 km E; Pinto Creek; 29°20'N, 100°31.6'W), 5 Jun 2004, W. N. Mathis (1♂, 1♀; USNM). *Llano*: Enchanted Rock (30°30.4'N, 98°49.1'W), 15 Jun 1953, W. W. Wirth (2♂; USNM). *Travis*: Austin (Zilker Park; 30°15.8'N, 97°46.3'W), 2 Jun 2004, W. N. Mathis (5♂, 3♀; USNM). *Val Verde*: Comstock (16 km N, Pecos River; 29°53.7'N, 101°09.1'W), 4 Jun 2004, W. N. Mathis (1♂; USNM).

*UTAH. Carbon*: Deadman Canyon (16 km NE Price; 39°41.7'N, 110°44'W; 2055 m), 19 Aug 2009, T. Zatwarnicki (1♂; USNM). *Box Elder*: Brigham City (41°30.6'N, 112°0.9'W), 26 May 1943, G. F. Knowlton (1♂; USNM). *Emery*: Green River (Green River; 38°59.6'N, 110°08.5'W; 1240 m), 31 Jul 2007, D. and W. N. Mathis (14♂, 1♀; USNM); Green River (3.3 km N, 39°01.7'N, 110°09.7'W; 1253 m), 30 Jul-16 Aug 2007, 2008, D. and W. N. Mathis (32♂, 3♀; USNM); San Rafael River (22.5 km SW Green River; 38°55.7'N, 110°24.5'W; 1270 m), 31 Jul 2007, D. and W. N. Mathis (7♂; USNM). *Garfield*: Boulder Mail Trail/Upper Calf Creek Falls (37°52.3'N, 111°28.3'W), 12 Jul 2000, M. Moody, C. R. Nelson (7♂; BYU); Last Chance Creek Junction, Smoky Mountain Road (37°20.8'N, 111°31.6'W; 1920 m), 19 Jun 2000, E. C. Green, W. N. Mendel (1♂; BYU); Upper Calf Creek Falls (37°02.6'N, 111°26.3'W), 2 Aug 2000, E. C. Green, M. Moody (1♂; BYU); Willow Tank-Hurricane Wash (37°23.2'N, 111°08'W; stock tank), 22 May 2001, D. and W. N. Mathis (1♂; USNM); 40-Mile Spring/Tank (69 km SE Escalante; 37°21'N, 111°4.9'W), 22 May 2001, D. and W. N. Mathis (2♂; USNM). *Grand*: Crystal Geyser (14.5 km SE Green River; 38°56.3'N, 110°08.1'W), 15 Aug 2008, D. and W. N. Mathis (4♂; USNM); Swasey Beach (15.3 km N Green River; 39°07'N, 110°06.6'W; Green River; 1255 m), 29 May-15 Aug 2007, 2008, D. and W. N. Mathis (31♂, 8♀; USNM); Thompson Spring (8.9 km N Thompson Springs; 39°02.3'N, 109°43.4'W; 1740 m), 1–20 Aug 2007, 2008, 2009, D. and W. N. Mathis, T. Zatwarnicki (16♂; USNM). *Kane*: Drip Tank Canyon (37°19.4'N, 111°31.8'W), 15 May 2001, D. and W. N. Mathis (15♂, 2♀; USNM); Drip Tank Canyon (37°19.4'N, 111°32'W), 5–6 Jul 2000, C. R. Nelson (3♂; BYU); East Fork of Virgin River (26.6 km N Kanab; 37°12.2'N, 112°41.4'W) 18 May 2001, D. and W. N. Mathis (10♂, 2♀; USNM); Kanab Canyon (6.5 km N Kanab; 37°08.7'N, 112°32.4'W), 14 May 2001, D. and W. N. Mathis (3♂; USNM); Seaman Wash at spring (37°07'N, 112°15'W; 1980 m) 13 Jul 2000, E. C. Green, W. N. Mendel (2♂; USNM); White House Spring (71 km E Kanab; 37°04.8'N, 111°53.4'W; 1250 m) 19 May 2001, D. and W. N. Mathis (2♂; USNM). *Salt Lake*: Midvale (40°36.6'N, 111°53.4'W), 29 Sep 1951, G. F. Knowlton (1♂; USNM). *Washington*: Beaver Dam Wash, Lytle Ranch (35°53.7'N, 113°55.3'W), 16 May 1987, D. E. Hardy (1♂; BYU); Shivwits (37°10.9'N, 113°45.4'W), 18 Jul 1970, W. J. Hanson (2♂, 2♀; USU).

*WASHINGTON. Adams*: Lower Crab Creek (46°54.6'N, 119°17.3'W), 28 Aug 1980, R. S. Zack (3♂, 4♀; WSU); Washtucna (19.5 km W; Sand Hills Coulee Park; 46°43.7'N, 118°39.7'W), 29 Mar 1978, R. S. Zack (2♂; WSU). *Asotin*: Asotin Creek (46°20.6'N, 117°03.2'W), 4 Jun 1930, J. M. Aldrich (2♂; USNM); Fields Spring State Park (46°04.9'N, 117°10.2'W), 25 Jul 1980, R. S. Zack (1♂; WSU). *Benton*: Rattlesnake Ridge (46°24.4'N, 119°36.3'W spring), 29 Aug 1994, R. S. Zack (1♂, 3♀; WSU); Rattlesnake Spring (46°30.4'N, 119°41.9'W), 21 Jun-17 Oct 1955, 1994, J. J. Davis, R. S. Zack (3♂, 6♀; USNM, WSU); Snively Spring, Hanford (46°27.1'N, 119°42.8'W), 24 Apr 1995, R. S. Zack (1♂, 16♀; WSU); West Richland (46°18.3'N, 119°21.7'W), 20 Jul 1973, N. E. Woodley (1♀; WSU). *Chelan*: Lake Chelan (47°50'N, 120°0.8'W), 30 Jul 1919, A. L. Melander (2♂, 1♀; USNM). *Franklin*: Handford Reach, Columbia River (46°24.6'N, 119°15.5'W), 20 Jul 2009, D. Mathis (10♂, 5♀; USNM); Levey (Snake River; 46°05'N, 118°52'W), 27 Jul 1992, D. Mathis (20♂; USNM); Palouse Falls (46°39.8'N, 118°13.4'W), 22 May 1971, W. N. Mathis (1♂; USNM); Pasco (3.2 km E; 46°51.1'N, 119°04.3'W), 8 Aug 1975, W. N. Mathis (2♂, 1♀; USNM); Pasco (NW; 46°21.5'N, 119°15.5'W), 29 Jul 1998, W. N. Mathis (17♂; USNM); Ringold (NW Pasco; 46°30.4'N, 119°15.3'W), 30 Jul 1998, W. N. Mathis (9♂, 1♀; USNM); Sacajawea State Park (46°12'N, 119°02.4'W; 100 m), 6 Jun 2006, D. and W. N. Mathis, T. Zatwarnicki (21♂, 9♀; TZ, USNM). *Grant*: Beverly (Columbia River; 46°50.4'N, 119°56.4'W), 29 May 2006, D. and W. N. Mathis (1♂, 1♀; USNM); Columbia National Wildlife Refuge (46°51.1'N, 119°32.2'W), 13 May 1959, H. F. Harwood (1♂, 5♀; USNM, WSU); Columbia National Wildlife Refuge (Mallard Lake; 46°51.1'N, 119°32.2'W), 12 Jul 1977, R. S. Zack (1♂; WSU); Crab Creek (4.8 km E Beverly; 46°50'N, 119°52'W), 30 May 2006, D. and W. N. Mathis (9♂; USNM); Crab Creek, lower, near Black Lake (46°50'N, 119°52'W), 28 Aug 1980, R. S. Zack (1♂; WSU); Othello (3.2 km W; 46°49.6'N, 119°13.4'W), 25 Jul 1992, R. S. Zack (3♂; WSU); Royal City (8 km W; 46°54'N, 119°47'W), 13 Jul 1978, R. S. Zack (3♂; WSU); Soap Lake (47°23.5'N, 119°29.1'W), 30 May 2006, D. and W. N. Mathis (1♂, 1♀; USNM); O'Sullivan Dam (46°59'N, 119°17.4'W), 23 May 1954, M. T. James (11♂, 15♀; USNM, WSU). *Kittitas*: Vantage (Columbia River; 46°56.5'N, 119°59.1'W), 29 May 2006, D. and W. N. Mathis (2♂; USNM). *Okanogan*: Brewster (48°05.8'N, 117°25'W), 17 Aug 1940, A. L. Melander (3♂, 1♀; USNM); Okanogan (15 km SW, Soap Lake; 48°14.1'N, 119°39'W), 23 Jul 1983, R. D. Akre, R. S. Zack (5♂; WSU). *Pend Oreille*: Indian Island near Furport (48°14.4'N, 117°10'W), 23 Jun 1982, R. S. Zack (5♂, 3♀; WSU); No Name Lake (48°17.8'N, 117°08.2'W), 23 Jun 1983, R. S. Zack (3♂; WSU). *Spokane*: Spokane (47°40.4'N, 117°25'W), 28 Aug 1911 (1♂; USNM). *Stevens*: Chewelah (48°16.6'N, 117°42.9'W), 30 Jun 1972, M. T. James (1♂; WSU). *Whitman*: Almota (46°42.2'N, 117°28.2'W), 2–28 Jun 1918, 1975, A. L. Melander, W. J. Turner (2♂; USNM, WSU); Pampa pond, 8 km W LaCrosse (46°34.6'N, 118°05'W), 7 Jul 1993, R. S. Zack (1♂; WSU); Steptoe Canyon (46°27.1'N, 117°12.4'W), 24Apr-3 Aug 1974, 1981, W. J. Turner, R. S. Zack (3♂; WSU); Wawawai (46°38.2'N, 117°22.8'W), 20 May 1911, 25 Jul-12 Aug 1957, R. H. Alvarado (6♂, 7♀; USNM, WSU); Willow Creek (13 km W LaCrosse; 46°34.6'N, 118°10'W), 11 Aug 1993, R. S. Zack (2♂, 2♀; WSU). *Yakima*: Prosser (7.6 km W; 46°11.3'N, 119°53'W), 14 Jun 1989, R. S. Zack (1♂; WSU); Toppenish (46°22.7'N, 120°18.5'W), 24 Apr 1968, J. Turner (3♂; WSU).

#### Distribution.

**Figure 74.**
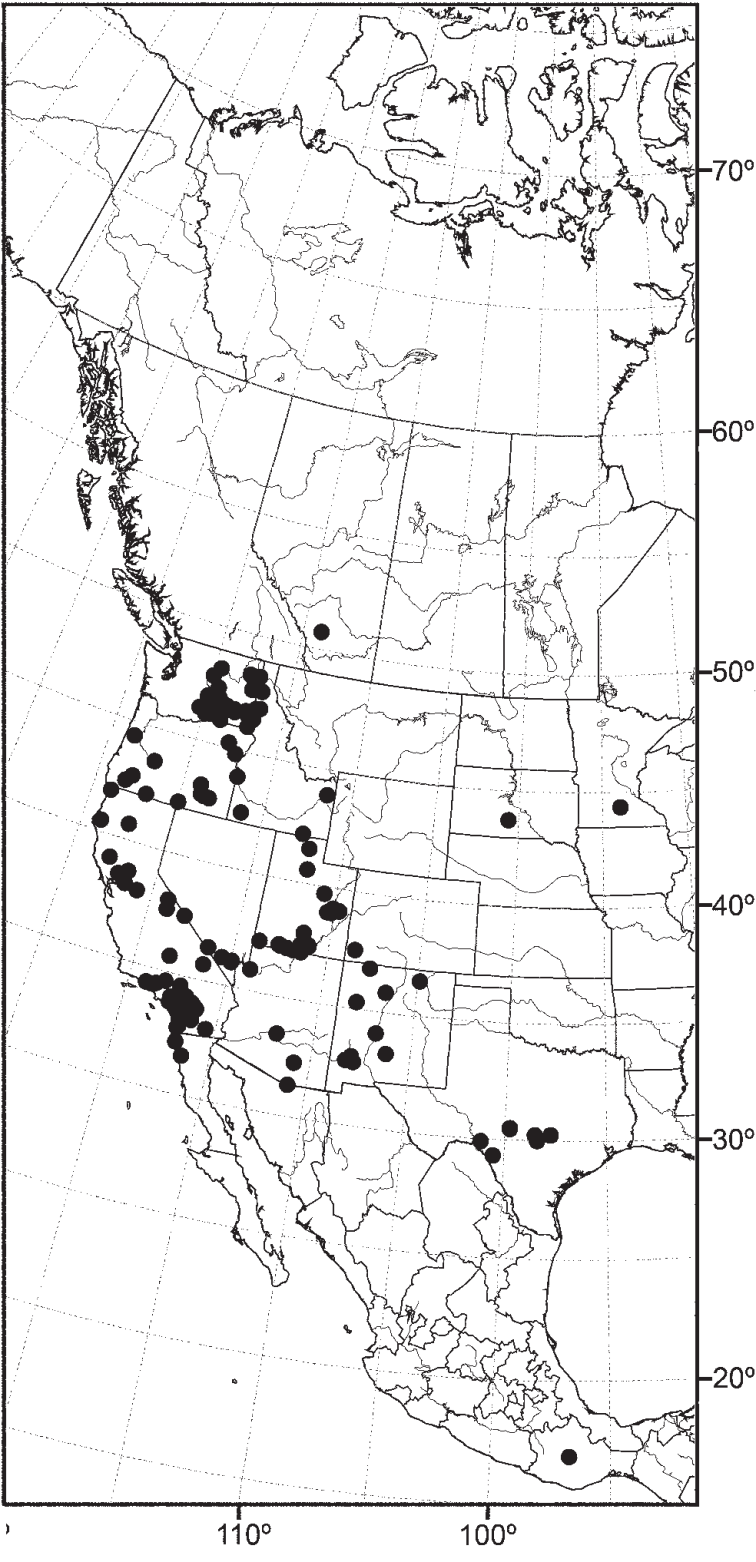
Distribution of *Allotrichoma wallowa* sp. n.

(Fig. 74) *Nearctic*: Canada (Alberta), Mexico (Baja California, Oaxaca), United States (Arizona, California, Colorado, Idaho, Montana, Minnesota, Nevada, New Mexico, Oregon, South Dakota, Texas, Utah, Washington).

#### Etymology.

The species epithet, *wallowa*, is a noun in apposition and refers to the Wallowa Mountains in northeastern Oregon. The headwaters of Goose Creek are in the Wallowas.

#### Remarks.

This species is similar and closely related to *Allotrichoma simplex*, differing from that species in the shape of the surstylar apex and especially in the shape of the male 5th sternal flap and process.

### Neotrichoma subgen. n.

urn:lsid:zoobank.org:act:E1775F62-BDBF-4F45-8806-9DD61B227C11

http://species-id.net/wiki/Neotrichoma

#### Type species.

*Allotrichoma atrilabre* Cresson, by present designation.

#### Diagnosis.

Minute to small to moderately small shore flies, body length 0.85–1.45 mm.

*Head*: Frons mostly unicolorous, at most with narrow, anterior fronto-orbits slightly lighter in color, lacking distinctively colored ocellar triangle; pseudopostocellar setae subequal in length to ocellar setae. Pedicel with well-developed, proclinate, dorsal seta. Facial coloration sexually dimorphic, males unicolorous and darker; face with dorsal 2/3 between antennal grooves shallowly carinate, becoming more prominent ventrad of facial grooves, slightly tuberculate; clypeus mostly bare, black, subshiny; palpus blackish.

*Thorax*: Mesonotum generally dark brown; chaetotaxy generally well developed; prescutellar acrostichal setae much larger than other acrostichal setae and more widely set apart; presutural supra-alar seta well developed, length subequal to notopleural setae; katepisternum with 2 setae, 2nd seta smaller and inserted below larger seta. Wing mostly milky white; veins behind costa brownish; alular marginal setulae short, less than 1/2 alular height. Legs: tibiae dark, concolorous with femora.

*Abdomen*: 5th tergite of male short, about 1/2 length of 4th tergite; 5th sternite unmodified. Male terminalia: cerci normally developed, not extended ventrally beyond ventral margin of epandrium; aedeagus and phallapodeme attached.

#### Discussion.

*Neotrichoma* is more closely related to the subgenus *Pseudohecamede* than to *Allotrichoma* sensu stricto. The structures of the male terminalia are especially diagnostic in this regard (unmodified 5th sternite, shorter 5th tergite, unmodified cerci).

#### Key to Males of the Subgenus *Neotrichoma*

**Table:** 

1	Abdominal tergites moderately to densely microtomentose, gray to grayish brown	*Allotrichoma (Neotrichoma) atrilabre* Cresson
–	Abdominal tergites mostly bare, shiny black	2
2	Face spotted (Florida, Brazil, Peru)	*Allotrichoma (Neotrichoma) baliops* sp. n.
–	face uniformly colored, not spotted (Dominica, Grenada)	*Allotrichoma (Neotrichoma) insulare* sp. n.

### Allotrichoma (Neotrichoma) atrilabre

Cresson

http://species-id.net/wiki/Allotrichoma_atrilabre

Figs 75 79

Allotrichoma atrilabre Cresson 1926: 252; 1942: 108 [list, eastern United States]. Deonier 1964: 120 [key, Iowa]; 1965: 501 [natural history]. Wirth 1965: 736 [Nearctic catalog]. Cole 1969: 397 [list]. Mathis and Zatwarnicki 1995: 150 [world catalog].
Allotrichoma yosemite Cresson 1926: 252; 1942: 108 [list, Rocky Mountains and westward of United States]. Wirth 1965: 736 [Nearctic catalog]; 1968: 5 [Neotropical catalog]. Cole 1969: 397 [list]. Mathis and Zatwarnicki 1995: 154 [world catalog]. New Synonym.

#### Description.

This species is distinguished from congeners by the following combination of characters: Small shore flies, body length 1.15–1.45 mm. *Head*: Frons mostly unicolorous, at most with narrow, anterior fronto-orbits slightly lighter in color, lacking distinctively colored ocellar triangle; pseudopostocellar setae subequal in length to ocellar setae. Pedicel with well-developed, proclinate, dorsal seta. Facial coloration sexually dimorphic, males unicolorous and darker; face with dorsal 2/3 between antennal grooves shallowly carinate, becoming more prominent ventrad of facial grooves, slightly tuberculate; clypeus mostly bare, black, subshiny; maxillary palpus blackish.

*Thorax*: Mesonotum generally dark brown; chaetotaxy generally well developed; prescutellar acrostichal setae much larger than other acrostichal setae and more widely set apart; presutural supra-alar seta well developed, length subequal to notopleural setae; katepisternum with 2 setae, 2nd seta smaller and inserted below larger seta. Wing mostly milky white; veins behind costa brownish; alular marginal setulae short, less than 1/2 alular height; costal vein ratio 0.34–0.44; M vein ratio 0.33–0.36. Legs: tibiae dark, concolorous with femora.

**Figures 75–78.**
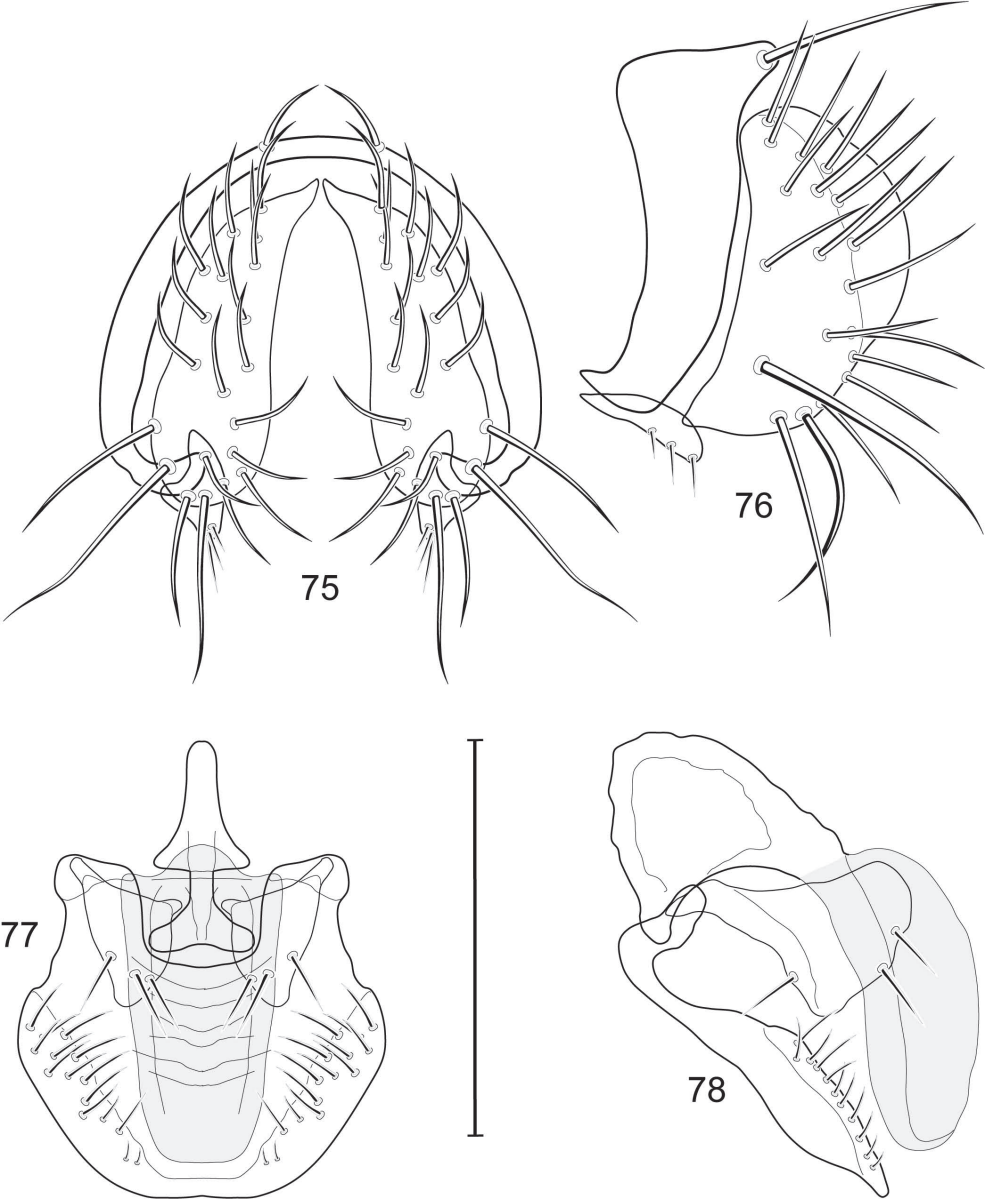
Illustration of *Allotrichoma atrilabre* Cresson (male) **75** epandrium, cerci, surstylus, posterior aspect **76** same, lateral aspect **77** aedeagus, phallapodeme, gonite, hypandrium, ventral aspect **78** same, lateral aspect. Scale bar = 0.1 mm.

*Abdomen*: 5th sternite transversely narrow, shallowly arched, bearing numerous setulae. Male terminalia (Figs 75–78): Epandrium in posterior view (Fig. 75) uniformly and broadly as an inverted U, slightly broader ventrally and ventral apex abruptly curved medially; in lateral view (Fig. 76) moderately narrow, height 3X width, widest dorsally, dorsal surface slightly produced posteriorly, thereafter ventrally slightly tapered and shallowly curved anteriorly, ventral margin produced anteriorly as a short, pointed extension; cercus in posterior view (Fig. 75) becoming broader ventrally, ventral margin broadly rounded, dorsal margin slightly produced and pointed medially, in lateral view (Fig. 76) with anterior margin shallowly curved, posterior margin rounded, slightly narrower dorsally than ventrally; surstylus a rod-like, nearly horizontal, slender process that bears prominent setulae posteriorly; aedeagus in ventral view (Fig. 77) elongate, narrowly rectangular, almost parallel sided, base rounded, not cleft apically, in lateral view (Fig. 78) very shallowly curved, broadly elliptical, apex broadly rounded; phallapodeme in lateral view (Fig. 78) well developed, keel broadly triangular, apex broadly but asymmetrically rounded, in ventral view (Fig. 77) as a double T (2 cross bars); gonite in lateral view (Fig. 78) short, as high or higher than long, apical margin irregularly truncate, bearing 2–3 setulae, in ventral view (Fig. 77) short, more or less triangular; hypandrium in ventral view (Fig. 77) broadly U-shaped, arms angled posterolaterally, in lateral view (Fig. 78) with base slightly hooked, becoming wider apically, bearing numerous setulae along apical half.

#### Type material.

The holotype male of *Allotrichoma atrilabre* Cresson is labeled “Lansdale [Montgomery County] P[ennsylvani]a[,] VII, 12, 1908 [12 Jul 1908]/♂/TYPE, Allotrichoma ATRILABRIS E.T. Cresson, Jr. [red; species and generic names handwritten]/Loaned by A.N.S.P.” The holotype is double mounted (glued to a paper triangle), is in poor condition (fore- and midlegs on right side and left wing missing; right wing, left 1st flagellomere, and right hindleg removed and glued to a second paper triangle; abdomen removed, dissected, structures stored in an attached microvial of glycerin), and is deposited in the ANSP (6320). Cresson (1926) also listed two male and one female paratopotypes (ANSP).

The holotype male of *Allotrichoma yosemite* Cresson is labeled “Yosemite Valley[,] V,22,'08, [22 May 1908] Cal[ifornia]./♂/TYPE Allotrichoma YOSEMITE E.T.Cresson,Jr. [red; species and generic names handwritten]/Loaned by A.N.S.P.” The holotype is double mounted (minuten in a rectangular card), is in good condition (right wing torn; abdomen removed, dissected, the parts stored in an attached microvial of glycerin), and is deposited in the ANSP (6321). Cresson (1926) also listed one male and eight female paratopotypes (ANSP).

#### Type locality.

United States. Pennsylvania. Montgomery: Lansdale (40°14.5'N, 75°17.1'W).

#### Other specimens examined from the New World.

CANADA. *BRITISH COLUMBIA*. Langley (49°04.4'N, 122°34.4'W), 9 Aug 1917, A. L. Melander (1♂; USNM). Savona (50°45.2'N, 120°50.6'W), 19 Jul 1988, R. Danielsson (1♂; ZIL).

*ONTARIO*. Hearst (75 km W; 49°52'N, 85°21.8'W), 9 Jul 1954, A. H. Sturtevant (1♂; USNM).

MEXICO. *BAJA CALIFORNIA*. Arroyo Grande (28°43.3'N, 113°57'W), 27 Mar 1964, M. E. Irwin (♂, 1♀; UCR). San Felipe (5 km N, 31°03'N, 114°49'W), 25 Aug 1964, J. C. Ball (1♀; UCR). Sierra San Pedro Martir (31°02.7'N, 115°28'W), 15 Jun 1953, P. H. Arnaud, Jr. (6♂; USNM).

*GUERRERO*. Chilpancingo (13 km E; 17°33.1'N, 99°30'W), 7 Aug 1962, N. Marston (4♂, 1♀; KSU).

*JALISCO*. Lagos de Moreno (4.5 km E; 21°21.5'N, 101°55.8'W), 27 Jul 1962, N. Marston (1♂, 4♀; KSU).

*MICHOACAN*. Morelia (18 km N, 19°41.9'N, 101°11.2'W), 28 Jul 1962, N. Marston (1♂; KSU).

*MORELOS*. Cuernavaca (18°56’N, 99°13.9’W), 4 Aug 1938, L. J. Lipovsky (1♀; KU).

*NUEVO LEON*. China (16 km NE; 25°47.3'N, 99°12.5'W), 18 Jul 1954 (1♀; KU).

*SINALOA*. Culiacán (24°48.2'N, 107°23.3'W), 21 Jan 1951, A. A. Hubert (1♀; KU). Los Mochis (26°15.6'N, 109°03.1'W), 24 Aug 1964, E. I. Schlinger (1♂; UCR).

UNITED STATES. *ALABAMA. Mobile*: Theodore (30°32.9'N, 88°10.5'W), 21 Jun 1917 (1♀; CU).

*ARIZONA. Cochise*: Apache (21 km S; 31°38'N, 109°11'W), 13 Aug 1970, J. G. Rozen (1♀; AMNH); Portal (8 km W; 31°53'N, 109°12.3'W), Southwest Research Station, 25 Jun 1955, M. Statham (1♀; AMNH). *Coconino*: Flagstaff (35°11.9'N, 111°39.1'W), 11 Jun 1909, F. C. Pratt (2♂, 3♀; USNM). *Pima*: Box Canyon, Santa Rita Mountains (31°48.1'N, 110°48.5'W), 12 Jun 1968, A. Menke, O. S. Flint (1♂; USNM). *Yavapai*: Prescott (34°32.4'N, 112°28.1'W), 15 Jun 1959, A. H. Sturtevant (1♀; USNM).

*ARKANSAS. Franklin* County (35°35'N, 93°50'W), 3 Jun 1965, H. R. Dodge (1♀; USNM).

*CALIFORNIA. Calavaras*: Milton (38°01.9'N, 120°51.1'W), 21 Oct 1917, J. C. Bradley (8♂, 3♀; CU). *Humbolt*: Scotia (5.5 km S; 40°28'N, 124°06'W), 10 Jun 1965, T. W. Fisher (2♂; UCR). *Inyo*: Bishop (37°21.8'N, 118°23.7'W), 24 Jul 1940, L. J. Lipvosky (1♂; KU)*. Lassen*: Mt. Home (1 km N, Forest Service Road 18; 40°41.9'N, 121°18.7'W), 15 Jul 1989, R. S. Zack (19♂, 103♀; WSU). *Lassen*: Hallelujah Junction (39°46.5'N, 120°02.3'W), 22 Jun 1964, M. E. Irwin (2♂; UCR). *Mono*: Mammoth lakes (37°36.3'N, 119°0.7'W), 29 Jul 1940, L. C. Kultert (12♂, 5♀; KU). *Monterey*: Arroyo Seco (32°54.3'N, 116°35'W), 1 Jul 1948, W. W. Wirth (1♂; USNM). *Orange*: Irvine Lake (33°46.6'N, 117°43.1'W), 12 Oct 1963, M. E. Irwin (1♀; UCR); San Juan Creek (33°30'N, 117°39'W), 14 Jul 1965, R. Orth (2♀; UCR). *Riverside*: Hemet Lake (33°39.7'N, 116°42.4'W), 19 May 1965, T. W. Fisher (1♂, 1♀; UCR); Massacre Canyon (33°50.6'N, 116°59.8'W), 4 May 1965, M. E. Irwin (1♀; UCR); Riverside (33°57.2'N, 117°23.8'W), 25 Aug-4 Oct 1962, 1963, M. E. Irwin, E. I. Schlinger (7♀; UCR); Santa Ana River (33°57.7'N, 117°28.2'W), 26 Jun 1965, G. R. Ballmer (1♀; UCR); Temecula (33°29.3'N, 117°08.5'W), 29 Jun 1965, R. Orth (1♀; UCR); Vail Lake (33°29.4'N, 116°58.1'W), 29 Jun 1965, R. Orth (1♀; UCR). *San Bernardino*: Echo (35°18'N, 116°48.3'W), 10 Aug 1940, L. J. Lipovsky (2♂, 3♀; KU). *San Diego*: Jacumba Spring (32°37'N, 116°11.4'W), 28 Jun 1917, J. M. Aldrich (1♂; USNM); Lake Henshaw (8 km W; 33°14'N, 116°43'W), 19 Jun 1965, G. R. Ballmer (1♀; UCR). *San Luis Obispo*: Cayucos Creek (8 km W; 35°28.6'N, 120°52.5'W), 5 Aug 1962, E. I. Schlinger (1♀; UCR). *Santa Clara*: Livermore (48 km S; 37°35.7'N, 121°43.5'W), 10 Jul 1971, W. J. Turner (4♂, 16♀; WSU). *Shasta*: Logan Lake (18 km S Hat Creek; 40°39.8'N, 121°29'W), 14 Jul 1989, R. S. Zack (9♂, 9♀; WSU); Redding (40°35.2'N, 122°23.5'W), 12 Jun 1965 (1♀; UCR); Viola (40°31.1'N, 121°40.7'W), 19 May 1941 (1♀; KU). *Siskiyou*: Klamath River (5 km E; 41°51.7'N, 122°50'W), 28 Jul 1988, R. Danielsson (1♂; ZIL); Taylor Lake (41°21.7'N, 122°58.1'W; 1980 m), 28 Jul 1968, H. B. Leech (17♂, 6♀; CAS); Yreka (10 km NW; 41°45'N, 122°39'W), 11 Jun 1984, R. Danielsson (1♂; ZIL). *San Bernardino*: Echo (35°18'N, 116°48.3'W), 10 Aug 1940, L. J. Lipovsky (2♂, 3♀; KU). *Santa Barbara*: Santa Ynez River (34°36.5'N, 120°04.7'W), 23 Jun 1965, M. E. Irwin (1♀; UCR). *Tulare*: Terminus Reservoir (36°25'N, 119°01'W), 12 May 1965, T. W. Fisher (1♀; UCR); Three Rivers (36°26.3'N, 118°54.3'W), 5 Aug 1940, L. J. Lipovsky (1♀; KU).

*COLORADO. Larimer*: Fort Collins (near; 40°35.1'N, 105°05.1'W; stream), 11 Jun-8 Jul 1938, 1986, M. T. James, R. S. and V. L. Zack (2♀; CSU, WSU). *Moffat*: Hells Canyon (10 km W; 40°57'N, 108°43'W), 21 Jun 1946, M. T. James (2♀; CSU).

*DELAWARE. Sussex*: Lewes Angola Neck Park (38°40.3'N, 75°09.1'W), 21 Sep 2007, G. A. Foster, A. M. Welch (1♀; USNM).

*IDAHO. Benewah*: Chatcolet (47°22.3'N, 116°54.8'W), Aug 1915, A. L. Melander (2♂; USNM). *Latah*: Bear Creek (46°37.8'N, 116°32'W), 28 Aug 1990, R. S. Zack (2♂; WSU); Big Meadow Creek (46°44'N, 116°45.5'W), 31 Jul 1979, R. S. Zack (2♂, 3♀; WSU); Helmer (1.6 km W; 46°48'N, 116°29'W; farm pond), 28 Aug 1990, R. S. Zack (4♂; WSU).

*ILLINOIS. Champaign*: Mahomet (40°11.7'N, 88°24.3'W), 6 Aug 1914 (1♀; INHS); Urbana (40°06.6'N, 88°12.4'W), 22 Jul-21 Nov 1913, 1915 (1♂, 1♀; INHS). *Morgan*: Meredosia (39°49.9'N, 90°33.6'W), 19 Aug 1917 (1♀; INHS).

*IOWA. Boone*: Fraser Dam (42°07.7'N, 93°58.7'W), 4 Sep 1960, D. L. Deonier (1♂; USNM).

*KANSAS. Reno*: (37°57'N, 98°05'W), 22 Sep (1♂; KSU). *Riley*: Manhattan (39°11'N, 96°34'W), 14 Jul 1962, N. Marston (1♂; KSU).

*MICHIGAN. Cheboygan*: Bryant's Bog (45°32.8'N, 84°40.3'W), 16 Jun 1933, G. Grant (1♀; USNM).

*MINNESOTA. St. Louis*: Eagles Nest (47°50.4'N, 92°05.8'W), 26 Aug 1959, W. V. Balduf (1♀; INHS).

*MONTANA. Flathead*: Big Fork (13 km NE; 48°05.7'N, 113°58.2'W), 9 Aug 1969, B. A. Foote (1♀;USNM).

*NEVADA. Humboldt*: Golconda (40°57.2'N, 117°29.4'W), 11 Jul 1968, G. E. Bohart (1♂; USU). *Nye*: Springdale (3 km S; 37°0.7'N, 116°43.5'W), 26 May 1954, M. Cazier (1♀; AMNH). *Washoe*: Pyramid (40°04.5'N, 119°42.1'W), 4–5 Jul 1947, R. L. Usinger (1♂; USNM); Reno (39°33.1'N, 119°51.1'W), 2 Jul 1927, E. P. Van Duzee (1♂; USNM); Verdi (39°31.1'N, 119°59.3'W), 27 Jun 1966, R. L Brumley (3♂, 2♀; USNM).

*NEW JERSEY. Ocean*: Tuckerton (39°36.2'N, 74°20.8'W), 17 Jun 2004, D. and W. N. Mathis (1♂, 4♀; USNM).

*NEW MEXICO. Catron*: Gila River (33°13.6'N, 106°15.1'W; 1750 m), 15 Aug 2007, D. and W. N. Mathis (6♂; USNM). *Chaves*: Roswell (9 km E; Pecos River; 33°23.8'N, 104°24'W; 1060 m), 10 Aug 2007, D. and W. N. Mathis (3♂, 2♀; USNM). *Eddy*: Black River (near Rattlesnake Springs; 32°05.2'N, 104°28.3'W; 1110 m), 11 Aug 2007, D. and W. N. Mathis (1♀; USNM). *Grant*: Mangas Springs (32°50.5'N, 108°30.7'W), 25 Jun 1953, W. W. Wirth (7♂, 5♀; USNM); Mimbres River (NM Highways 61 and Royal John Mine Road; 32°43.8'N, 107°52'W; 1665 m), 2–22 Aug 2007, 2008, 2009, D. and W. N. Mathis (10♂, 4♀; USNM); Mimbres River (NM Hwy. 35; 19 km N NM Hwy 152; 32°56.9'N, 108°01.1'W), 2 Aug 2008, D. and W. N. Mathis (9♂; USNM). *Lincoln*: Ruidoso (33°19.9'N, 105°40.4'W), 26 Jun 1940, L. J. Lipovsky (4♀; KU). *Sandoval*: La Cueva (Junction of Highways 126 and 4; 35°52'N, 106°38.4'W; 2342 m), 6 Aug 2007, D. and W. N. Mathis (1♀; USNM).

*OREGON. Baker*: Goose Creek (35 km E Baker City; 44°49.2'N, 117°27.8'W; 825 m), 7 Jun 2006, D. and W. N. Mathis, T. Zatwarnicki (17♂, 5♀; TZ, USNM). *Benton*: Finley Wildlife Refuge (44°24.6'N, 123°19.5'W), 18 Jun 1976, W. N. Mathis (1♂; USNM). *Deschutes*: Tumalo Reservoir (44°10.9'N, 121°19.8'W), 23 Jun 1953, G. F. Knowlton (1♂, 1♀;KU). *Douglas*: North Umpqua River (42°43.2'N, 122°60'W; 250 m), 31 Jul 2005, D. and W. N. Mathis (4♂, 1♀; USNM). *Harney*: King Mountain Lookout turnoff (43°50.6'N, 118°57.2'W; 1537 m), 8 Aug 2005, D. and W. N. Mathis (11♂, 5♀; USNM); Malheur National Wildlife Refuge, Dredger Pond (43°16'N, 118°50.7'W), 29 Aug 1983, R. S. Zack (1♀; WSU); Page Springs Campground, Blitzen River (42°47'N, 118°51'W; 1300 m), 6 Aug 2005, D. and W. N. Mathis (3♂; USNM); Pikes Creek (42°34.5'N, 118°31.7'W; 1320 m), 7 Aug 2005, D. and W. N. Mathis (11♂, 3♀; USNM); Steens Mountain, Fish Lake Campground (42°44.3'N, 118°38.7'W; 2250 m), 6 Aug 2005, D. and W. N. Mathis (7♂, 3♀; USNM). *Jackson*: Prospect (42°45'N, 122°29.3'W), 10 Aug 1951, A. H. Sturtevant (2♂, 1♀; USNM). *Klamath*: Klamath River (12 km W Keno; 42°07.6'N, 121°56'W), 27 Jul 1988, R. Danielsson (2♂; ZIL). *Lake*: Hart Mountain National Antelope Refuge (42°30'N, 119°36.1'W), 27 Aug 1983, R. S. Zack (2♂, 2♀; WSU); Lakeview (43.5 km E; Drake Creek; 42°11'N, 119°59.3'W), 29 Jul 2005, D. and W. N. Mathis (16♂, 9♀; USNM). *Lane*: Hand Creek (43°56.3'N, 123°51.9'W), 11 Jul 1972, W. N. Mathis (1♂; USNM). *Umatilla*: McNary Dam (45°56.2'N, 119°17.9'W), 17 Apr 1993, R. S. Zack (1♂; WSU). *Union*: Ladd Canyon (45°12.8'N, 118°01.6'W; 1295 m), 30 Jul 1977, E. J. Davis, R. S. Zack (4♂, 2♀; WSU). *Wasco*: Maupin (45°10.5'N, 121°04.9'W), 10 Aug 1950, A. H. Sturtevant (1♂; USNM).

*TEXAS. Brewster*: Boquillas Canyon, Big Bend National Park (29°12.7'N, 102°53.2'W), 20 Jun 1953, W. W. Wirth (2♂; USNM). *Blanco*: Miller Creek (30°15.2'N, 98°31.7'W; 410 m), 3 Jun 2004, W. N. Mathis (7♂, 6♀; USNM). *Cameron*: Southmost (25°51.2'N, 97°23.4'W), 13 Jun 1958 (1♀; KU). *Kimble*: Junction (South Llano River; 30°29.5'N, 99°45.4'W), 4–6 Jul 1986, R. S. Zack (1♂, 2♀; WSU); Junction (South Llano River; 30°29.6'N, 99°45.1'W; 510 m), 4 Jun 2004, W. N. Mathis (3♂; USNM). *Kinney*: Del Rio (30 km E; 29°20'N, 100°31.6'W), 5 Jun 2994, W. N. Mathis (7♂, 5♀; USNM). *Sabine*: Yellowpine, Toledo Bend, Lakeview Campground (31°15.6'N, 93°49.5'W), 30 Jun 1986, R. Danielsson (1♂; ZIL). *San Patricio*: Mathis (6.5 km S; Nueces River; 28°02.8'N, 97°51.8'W; 18 m), 5 Jun 2004, W. N. Mathis (1♂; USNM). *Val Verde*: Comstock (16 km N, Pecos River; 29°53.7'N, 101°09.1'W), 4 Jun 2004, W. N. Mathis (1♂; USNM).

*UTAH. Cache*: Providence (41°42.4'N, 111°49'W), 19 Aug 1954, G. F. Knowlton (1♀; KU). *Emery*: Green River (Green River; 38°59.6'N, 110°08.5'W; 1240 m), 31 Jul 2007, D. and W. N. Mathis (2♂; USNM). *Garfield*: Boulder Mail Trail/Upper Calf Creek Falls (37°52.3'N, 111°28.3'W), 12 Jul 2000, M. Moody, C. R. Nelson (13♀; BYU);. *Grand*: Crystal Geyser (14.5 km SE Green River; 38°56.3'N, 110°08.1'W; 1000 m), 15 Aug 2008, D. and W. N. Mathis (1♂; USNM); Swasey Beach (15.3 km N Green River; 39°07'N, 110°06.6'W; Green River; 1255 m), 30 Jul-15 Aug 2007, 2008, D. and W. N. Mathis (7♂, 1♀; USNM); Thompson Spring (8.9 km N Thompson Springs; 39°02.3'N, 109°43.4'W; 1740 m), 1 Aug 2007, D. and W. N. Mathis (2♂; USNM). *Kane*: Last Chance Creek Junction, Smoky Mountain Road (37°20.8'N, 111°31.6'W; 1920 m), 19 Jun 2000, E. C. Green, W. N. Mendel (1♂, 1♀; BYU). *Kane*: Kanab (37°02.8'N, 112°31.6'W), 14 Jul 1948, G. F. Knowlton (1♂, 1♀; USNM). *Millard*: Delta (39°21.1'N, 112°34.6'W), 14 Aug 1940, L. J. Lipovsky (1♂; KU). *Salt Lake*: Magna (40°42.5'N, 112°06.1'W), 2 Jun 1954, G. F. Knowlton (1♀; KU). *San Juan*: Dry Wash Reservoir (19 km W Blanding: 37°46.5'N, 109°32.4'W; 2355 m), 2 Aug 2007, D. and W. N. Mathis (1♂; USNM); Johnson Canyon (21 km W Blanding: 37°47.6'N, 109°30.7'W; 2335 m), 2 Aug 2007, D. and W. N. Mathis (1♂; USNM).

*VIRGINIA. Fairfax*: Fairfax (38°50.5'N, 77°18.5'W), Jul 1954, M. R. Wheeler (2♂; USNM); Great Falls (Patowmack Canal; 39°00.1'N, 77°15.2'W), 25 Jul-29 Aug 2006, 2007, D. and W. N. Mathis (3♂; USNM); Great Falls (quarry; 38°59.1'N, 77°14.8'W; 50 m), 13 Jun 2007, D. and W. N. Mathis (1♂, 1♀; USNM); Great Falls (river trail; Potomac River; 38°59.1'N, 77°14.6'W; 60 m), 3 Apr 2007, D. and W. N. Mathis (1♂, 2♀; USNM); Turkey Run (mouth; 38°57.9'N, 77°09.4'W), 22 May-17 Sep 2006, 2007, D. and W. N. Mathis, H. B. Williams, T. Zatwarnicki (13♂, 5♀; USNM). *Roanoke*: Salem (Roanoke River; 37°16.1'N, 80°02.2'W; 300 m), 23 Sep 2007, D. and W. N. Mathis (1♂, 1♀; USNM). *Spotsylvania*: Rappahannock River (38°18.8'N, 77°32.5'W), 3 Jul-10 Oct 2007, D. and W. N. Mathis (13♂, 1♀; USNM). *Stafford*: Aquia Harbour, Lions Park (38°27'N, 77°23.3'W), 1 Jun 2007, D. and W. N. Mathis (1♂; USNM); Falmouth (38°19.2'N, 77°28.1'W; Rappahannock River; 9 m), 18 Apr-30 Jun 2007, 2008, D. and W. N. Mathis (7♂, 2♀; USNM). *York*: Maury Lake (ca. James River; 37°02.5'N, 76°29.2'W), 19 Aug 2006, D. and W. N. Mathis (7♂, 1♀; USNM).

*WASHINGTON. Adams*: Lower Crab Creek (46°54.6'N, 119°17.3'W), 28 Aug 1980, R. S. Zack (6♂, 10♀; USNM). *Benton*: near Hanford townsite (46°35'N, 119°23'W), 20 Apr 1994, R. S. Zack (1♂; WSU); Snively Spring, Hanford (46°27.1'N, 119°42.8'W), 11 Apr 1994, R. S. Zack (1♂; WSU). *Franklin*: Levey (Snake River; 46°05'N, 118°52'W), 27 Jul 1992, D. Mathis (2♂; USNM). *Grant*: O'Sullivan Dam (46°59'N, 119°17.4'W), 22 May 1954, M. T. James (1♂; WSU); Othello (3.2 km W; 46°49.6'N, 119°13.4'W), 25 Jul 1992, R. S. Zack (4♀; WSU). *Lincoln*: Wilbur Pond (47°45.6'N, 118°42.3'W), 8 Jul 1993, R. S. Zack (1♀; WSU). *Okanogan*: Cameron Lake (48°18'N, 119°32.6'W), 22 Jul 1983, R. D. Akre, R. S. Zack (3♂, 2♀; WSU); Conconully (29 km NW, Tiffany Meadow; 48°41.3'N, 119°58.1'W; 1920 m), 4 Jun 1975, N. E. Woodley (1♀; WSU); Cook Lake (47°33.6'N, 119°28'W), 22 Jul 1983, R. D. Akre, R. S. Zack (12♂, 19♀; WSU); Okanogan (15 km SW, Soap Lake; 48°14.1'N, 119°39'W), 23 Jul 1983, R. D. Akre, R. S. Zack (10♂, 13♀; WSU); Penley Lake (about 8 km SE Okanogan; 48°17.6'N, 119°32.5'W), 22 Jul 1983, R. D. Akre, R. S. Zack (54♂, 78♀; WSU). *Pend Oreille*: Indian Island near Furport (48°14.4'N, 117°10'W), 23 Jun 1982, R. S. Zack (5♂, 3♀; WSU); Usk (11 km E; 88°18.4'N, 117°14'W), 23 Jun 1982, R. S. Zack (3♂, 1♀; WSU). *Pierce*: Cayuse Pass (46°52'N, 121°32.3'W), 8 Aug 1979, R. S. Zack (5♂, 4♀; WSU); Sunrise Point, Mount Rainier National Park (1.6 km W; 46°55.2'N, 121°35.2'W), 8 Aug 1979, R. S. Zack (1♂; WSU). *Whitman*: Almota (46°42.2'N, 117°28.2'W), 27 Apr 1972, W. J. Turner (1♀; WSU); Pampa pond, 8 km W LaCrosse (46°34.6'N, 118°05'W), 7 Jul 1993, R. S. Zack (4♀; WSU). *Spokane*: Elokia Lake (48°01.6'N, 117°22.7'W), 24 Jun 1982, R. D. Akre, R. S. Zack (1♂; WSU).

**Figure 79.**
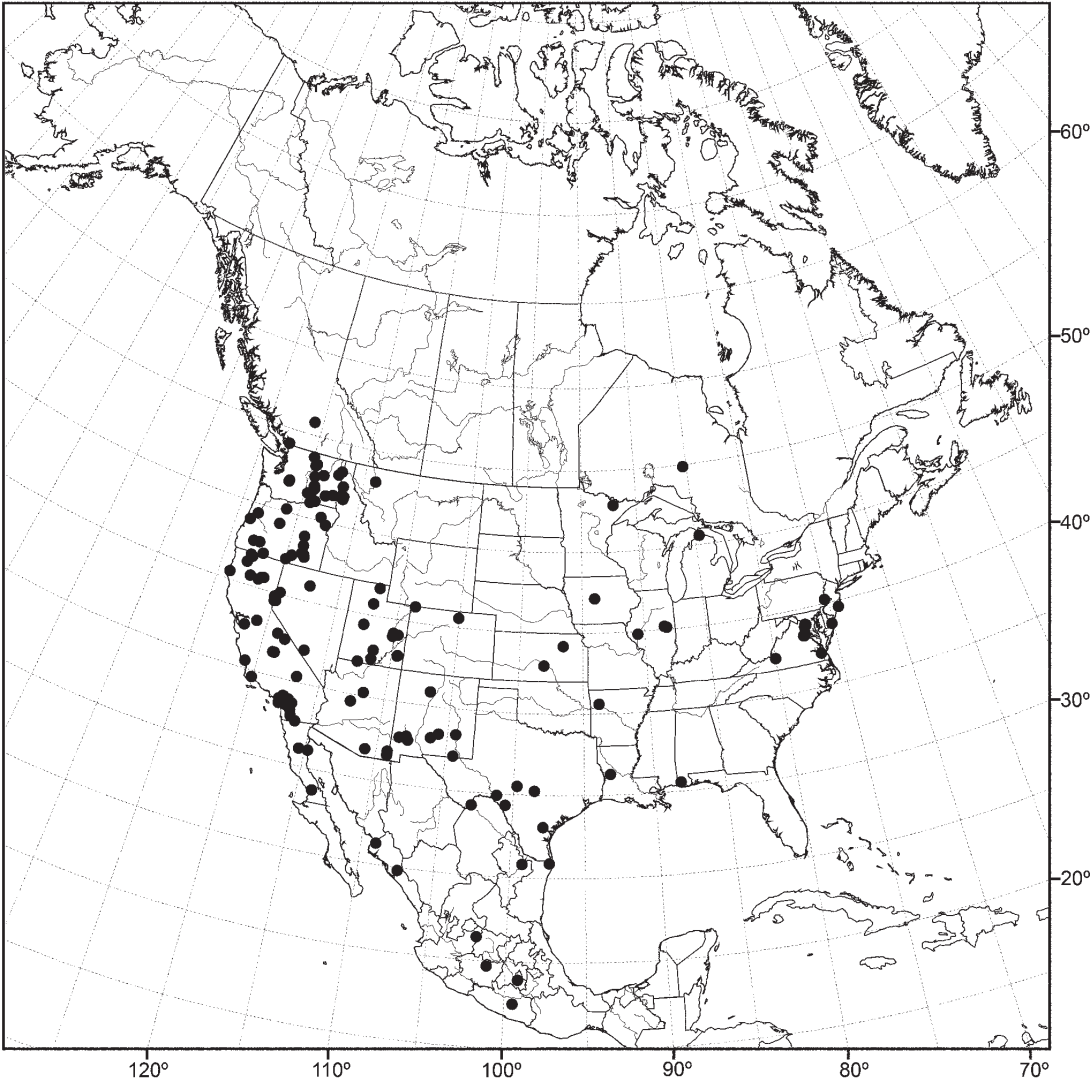
Distribution of *Allotrichoma atrilabre* Cresson.

Distribution (Fig. 79). *Nearctic*: Canada (British Columbia), United States (Alabama, Arizona, Arkansas, California, Colorado, Delaware, Idaho, Illinois, Iowa, Kansas, Michigan, Minnesota, Montana, Nevada, New Jersey, New Mexico, Oregon, Pennsylvania, Texas, Utah, Virginia, Washington). *Neotropical*: Mexico (Guerrero, Jalisco, Michoacan, Morelos, Nuevo Leon, Sinaloa).

#### Natural history.

Deonier (1965: 501) found this species to be rare in limnic wrack, mud shore, and sand shore habitats in Iowa. Runyan and Deonier (1979) illustrated the egg of this species.

### Allotrichoma (Neotrichoma) baliops sp. n.

urn:lsid:zoobank.org:act:C1DBEAF7-6271-444B-8FB3-3F22D91A6C65

http://species-id.net/wiki/Allotrichoma_baliops

**Figure 80.**
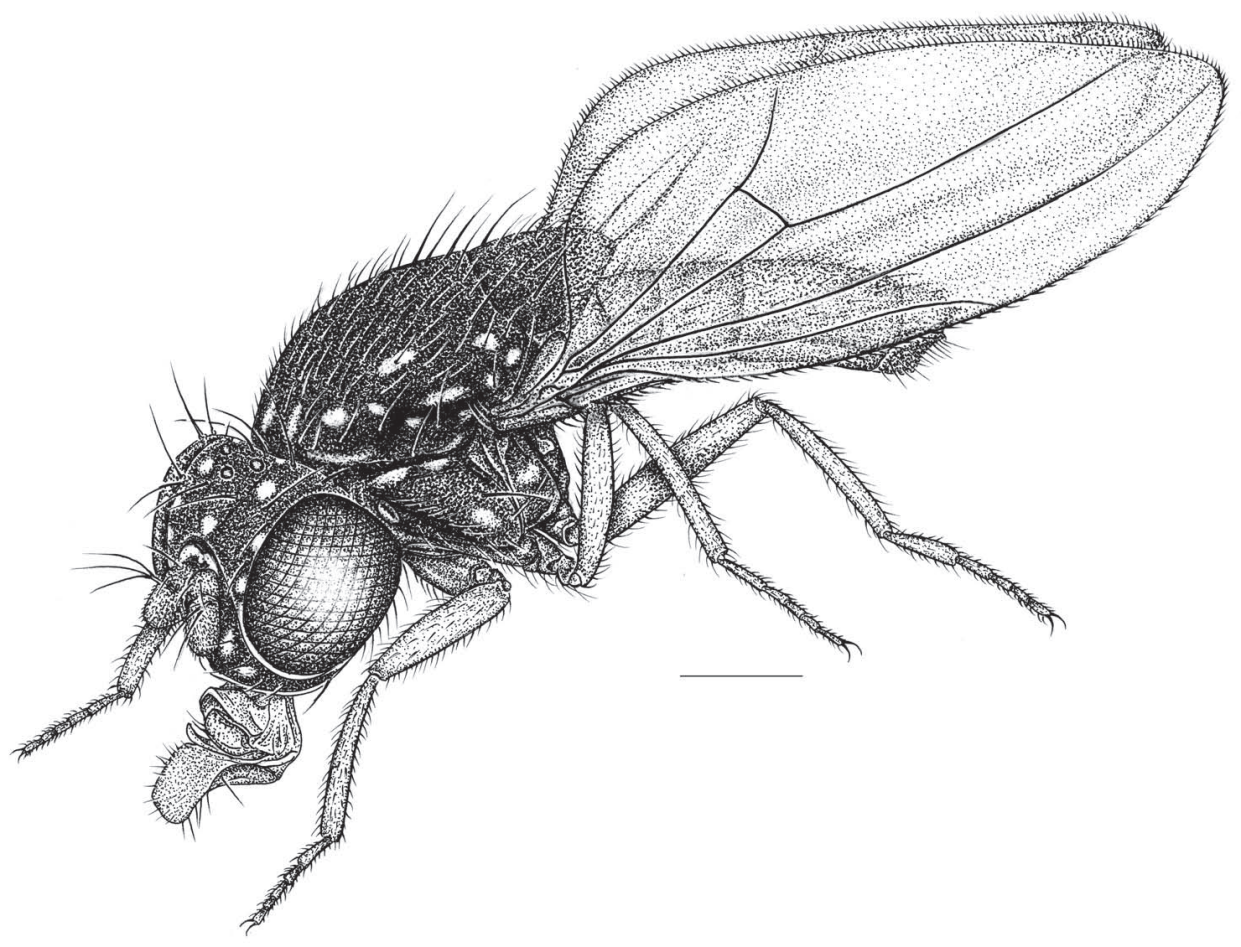
Habitus of *Allotrichoma baliops* sp. n.

Fig. 80 [Fig F30] 85

#### Description.

This species is distinguished from congeners by the following combination of characters: Small shore flies, body length 1.00–1.35 mm (Fig. 80). *Head*: Frons distinctly 2-toned, mostly uniformly blackish brown but with distinct gray, microtomentose spots at anterior apex of triangular mesofrons and at each posterior angle near vertex, 2 gray, microtomentose spots on anterior fronto-orbits; pseudopostocellar setae subequal in length to ocellar setae. Pedicel with well-developed, proclinate, dorsal seta. Facial coloration distinctly 2-toned, background blackish brown to black, with silver to silvery gray spots dorsad of antennal bases, at dorsal apex of pedicel, in antennal grooves, 2 spots on lateral portion of face, along epistomal margin; parafacial silvery gray; face with dorsal 2/3 between antennal grooves shallowly carinate, becoming more prominent ventrad of facial grooves, slightly tuberculate; clypeus silver to silvery gray; maxillary palpus blackish.

*Thorax*: Mesonotum generally dark brown, with silvery gray, microtomentose spots medioanteriorly just lateral of acrostichal setulae, 4–5 spots lateral spots in irregular row from anterior portion to near posterior margin, postpronotum with posteromedial border with silvery gray stripe; notopleuron with silvery gray spotx ventrally, and posteriorly; anepisternum with 2 spots along dorsal margin and medially near posterior margin; katepisternum with dorsal, elongate spot; chaetotaxy generally well developed; prescutellar acrostichal setae much larger than other acrostichal setulae and more widely set apart; presutural supra-alar seta evident, length subequal to notopleural setae; katepisternum with 2 setae, 2nd seta smaller and inserted ventrad of larger seta. Wing mostly hyaline; veins behind costa brownish; alular marginal setulae short, less than 1/2 alular height; costal vein ratio 0.63–0.83; M vein ratio 0.35–0.40. Legs: tibiae dark, concolorous with femora.

**Figures 81–84. F30:**
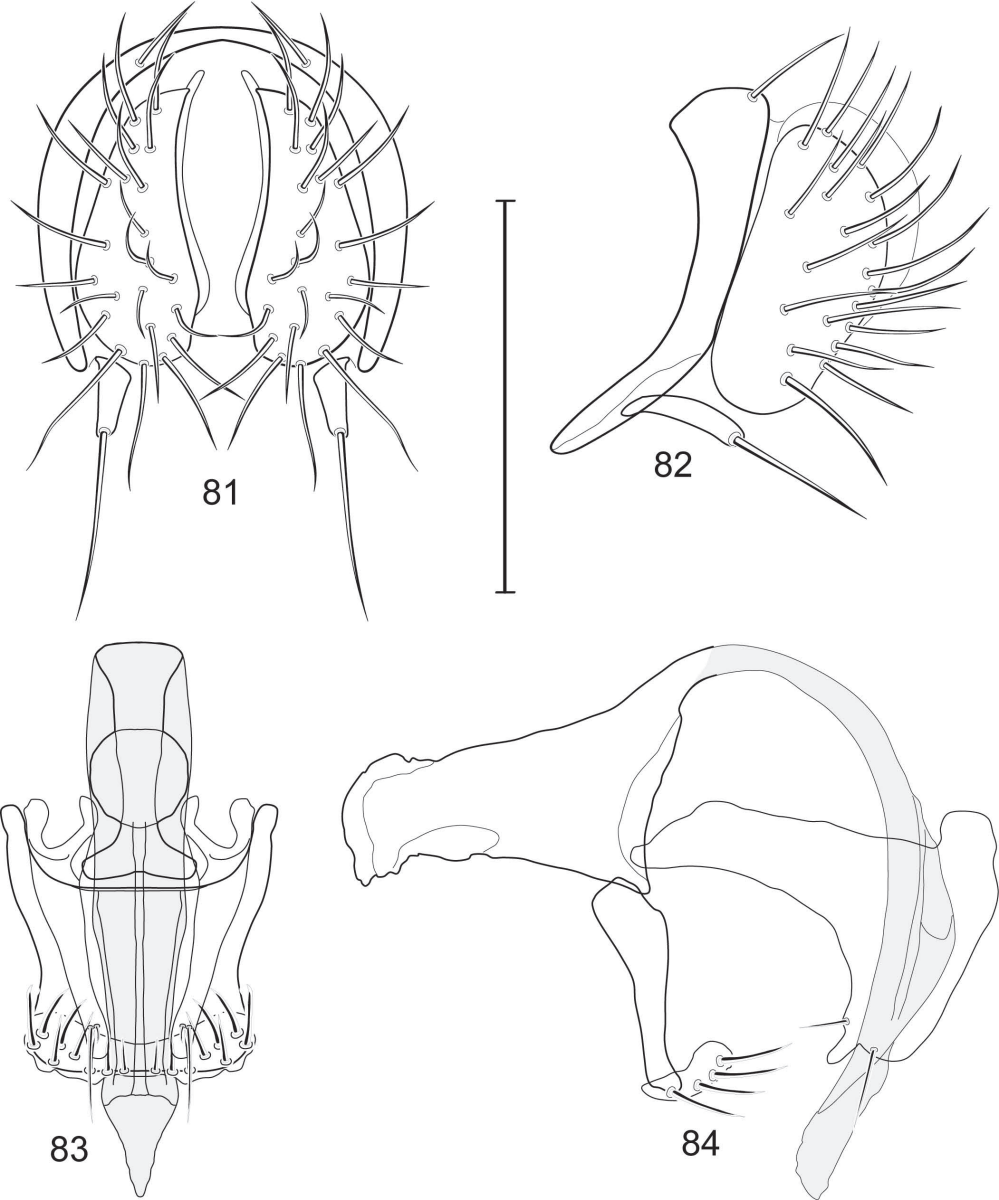
Illustration of *Allotrichoma baliops* sp. n. (male) **81** epandrium, cerci, surstylus, posterior aspect **82** same, lateral aspect **83** aedeagus, phallapodeme, gonite, hypandrium, ventral aspect **84** same, lateral aspect. Scale bar = 0.1 mm.

*Abdomen*: 5th sternite transversely narrow, slightly expanded laterally, bearing numerous setulae. Male terminalia (Figs 81–84): Epandrium in posterior view (Fig. 81) uniformly U-shaped, in lateral view (Fig. 82) narrow, elongate, curved anteriorly on ventral half; cercus in posterior view (Fig. 81) becoming broader ventrally, ventral margin rounded, dorsal margin pointed medially, in lateral view (Fig. 82) with anterior margin nearly straight, posterior margin rounded, slightly narrower ventrally than dorsally; surstylus a rod-like, slender process that bears an apical, prominent setula; aedeagus in ventral view (Fig. 83) elongate, slightly narrower apically, apex pointed, in lateral view (Fig. 84) gently arched, broadened subapically, thereafter tapered to a sharp point; phallapodeme in lateral view (Fig. 84) semirectangular, slightly tapered toward apex of keel, in ventral view (Fig. 83) T-shaped; gonite in lateral view (Fig. 84) very broad basally, abruptly narrowed to form a relatively short, posteroapical extension that apically bears 2 setulae and a short, digitiform process, in ventral view (Fig. 83) elongate, unevenly tapered toward apex, base irregularly excavated at basal margin; hypandrium in ventral view (Fig. 83) subquadrate with rounded corners and with posterior, short projections, in lateral view (Fig. 84) leg-like, elongate, broader basally, shallowly angulate.

#### Type material.

The holotype male *Allotrichoma baliops* is labeled “**USA. FL.** Monroe: Key West (Willie Ward Park; 24°32.9'N, 81°47.9'W), 12 Jan 2007, D. & Wayne N. Mathis/HOLOTYPE ♂ *Allotrichoma baliops* W. Mathis & T. Zatwarnicki USNM [red]/USNM ENT 00117962 [plastic bar code label].” The holotype male is double mounted (minuten in a block of plastic elastomer), is in excellent condition, and is deposited in the USNM. Twelve paratypes (6♂, 6♀; USNM) bear the same locality data as the holotype (dates from 12 Jan-11 Feb 2000, 2007). Other paratypes are as follows: *FLORIDA. Monroe*: Key West (Dog Beach; 24°32.8'N, 81°47.7'W), 12 Jan 2007, D. and W. N. Mathis (5♂, 2♀; USNM).

#### Type locality.

United States. Florida. Monroe: Key West (Willie Ward Park; 24°32.9'N, 81°47.9'W).

#### Other specimens examined from the New World.

Neotropical: *BRAZIL. PARANÁ*. Antonina (25°28.4'S, 48°40.9'W; beach/mangal), 9 Apr 2010, D. and W. N. Mathis (1♂; USNM).

*PERU. Lima*: Callao (12°04.1'S, 77°08.7'W), 17 Nov 1950, A. E. Michelbacher, E. S. Ross (1♂; CAS).

#### Distribution.

**Figure 85.**
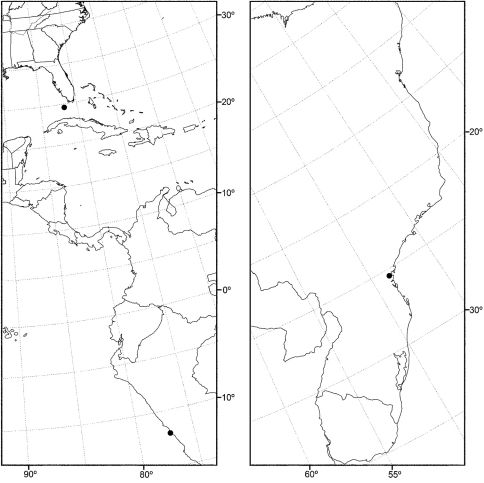
Distribution of *Allotrichoma baliops* sp. n.

(Fig. 85) *Nearctic*: United States (Florida). *Neotropical*: Brazil (Paraná), Peru (Lima).

#### Etymology.

The species epithet, *baliops*, is of Greek derivation, meaning spotted, and is a noun in apposition that alludes to the spotted face of this species.

#### Remarks.

The known distribution of this species is obviously disjunct, which is undoubtedly the result of sampling error, i.e., a lack of field work and collecting. Although specimens are apparently uncommonly collected, we suggest that this species will eventually be found at localities between and beyond those presently indicated. More field work by competent collectors is needed.

### Allotrichoma (Neotrichoma) insulare sp. n.

urn:lsid:zoobank.org:act:2102BF97-5479-409D-840D-B7C885B8D6F4

http://species-id.net/wiki/Allotrichoma_insulare

Fig. 86 90

#### Description.

This species is distinguished from congeners, especially those in *Neotrichoma*, by the following combination of characters: Minute to small shore flies, body length 0.86–1.35 mm. *Head*: Frons moderately 2–3-toned, mostly brown but with gray, microtomentose areas toward anterior apex of triangular mesofrons and at each posterior angle near vertex, whitish gray, microtomentose areas anteriorly on fronto-orbits; pseudopostocellar setae subequal in length to ocellar setae. Pedicel with well-developed, proclinate, dorsal seta. Facial coloration variegated from tan to gray to golden ventromedially; face with dorsal 2/3 between antennal grooves shallowly carinate, becoming more prominent ventrad of facial grooves, slightly tuberculate; clypeus mostly bare, black, subshiny; maxillary palpus blackish.

*Thorax*: Mesonotum generally brown with gray stripe anteriorly on either side of acrostichal setulae and more faintly through portion of dorsocentral track; chaetotaxy generally well developed; prescutellar acrostichal setae much larger than other acrostichal setulae and more widely set apart; presutural supra-alar seta well developed, length subequal to notopleural setae; katepisternum with 2 setae, 2nd seta smaller and inserted below larger seta. Wing mostly hyaline; veins behind costa brownish; alular marginal setulae short, less than 1/2 alular height; costal vein ratio 0.41–0.55; M vein ratio 0.44–0.45. Legs: tibiae dark, concolorous with femora.

**Figures 86–89.**
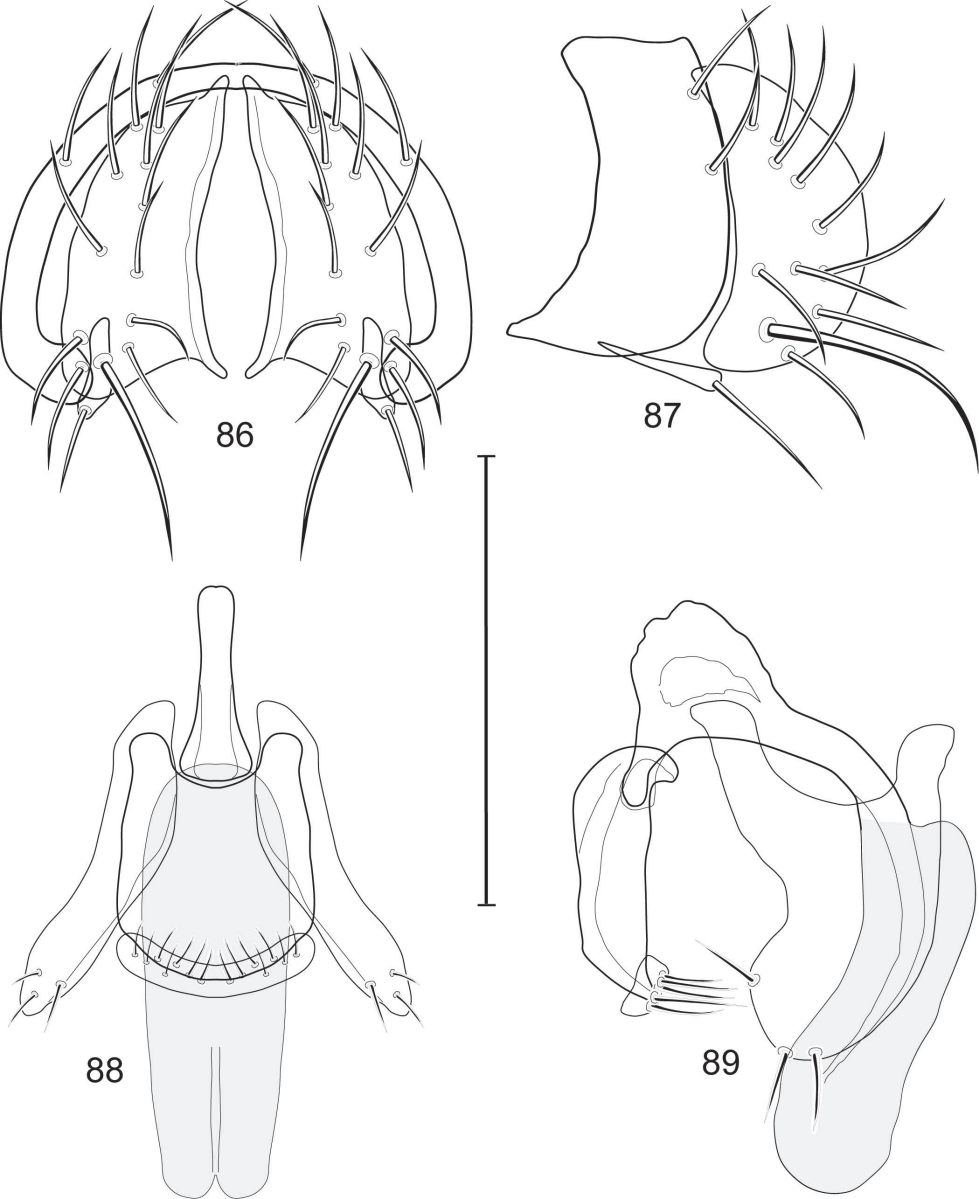
Illustration of *Allotrichoma insulare* sp. n. (male) **86** epandrium, cerci, surstylus, posterior aspect **87** same, lateral aspect **88** aedeagus, phallapodeme, gonite, hypandrium, ventral aspect **89** same, lateral aspect. Scale bar = 0.1 mm.

*Abdomen*: Abdomen shiny, sparsely microtomentose, black; 5th tergite short, length less than 1/2 4th; 5th sternite unmodified, lacking ventral process, transversely narrow, shallowly arched, bearing numerous setulae. Male terminalia (Figs 86–89): Epandrium in posterior view (Fig. 86) uniformly and broadly U-shaped, in lateral view (Fig. 87) moderately narrow, height twice width, slightly curved anteriorly at ventral margin; cercus in posterior view (Fig. 86) becoming broader ventrally, ventral margin sinuous with a short, medial projection, dorsal margin pointed medially, in lateral view (Fig. 87) with anterior margin shallowly curved, posterior margin rounded, slightly narrower dorsally than ventrally; surstylus a rod-like, slender process that bears an apical, prominent setula; aedeagus in ventral view (Fig. 88) elongate, almost parallel sided, base rounded, apical 1/3 deeply cleft, in lateral view (Fig. 89) gently sinuous, slightly broader apically; phallapodeme in lateral view (Fig. 89) with relatively short keel, irregularly rounded, in ventral view (Fig. 88) rod-like, slightly broader basally; gonite in lateral view (Fig. 89) with basal margin broadly excavated, forming 2 processes, thereafter apically slightly angulate, broadly formed to broadly rounded apex that bears 3 setulae, in ventral view (Fig. 88) elongate, almost parallel sided, curved laterally; hypandrium moderately deeply emarginate along posterior margin, forming a receptacle for base of phallapodeme, longer than wide, anterior margin broadly rounded.

#### Type material.

The holotype male *Allotrichoma insulare* is labeled “DOMINICA. Cabrits [Swamp] (15°35'N, 61°29'W)[,] 19 June 1991[,] W. N. & D. Mathis /HOLOTYPE ♂ *Allotrichoma insulare* W. Mathis & T. Zatwarnicki USNM [red]/USNM ENT 00117963 [plastic bar code label].” The holotype is double mounted (minuten in a block of plastic), is in excellent condition, and is deposited in the USNM. Seven paratypes (3♂, 4♀; USNM) are as follows: DOMINICA. Macoucheri (seashore), 1 Feb 1965, W. W. Wirth.

#### Type locality.

Dominica. Cabrits Swamp (15°35'N, 61°29'W).

#### Other specimens examined from the New World.

GRENADA. *St. John*: Palmiste (12°08.7'N, 61°44.4'W), 21 Sep 1996, W. N. Mathis (1♀; USNM). *St. Patrick*: Bathway Beach (12°12.6'N, 61°36.7'W), 18–20 Sep 1996, W. N. Mathis (1♂; USNM).

#### Distribution.

**Figure 90.**
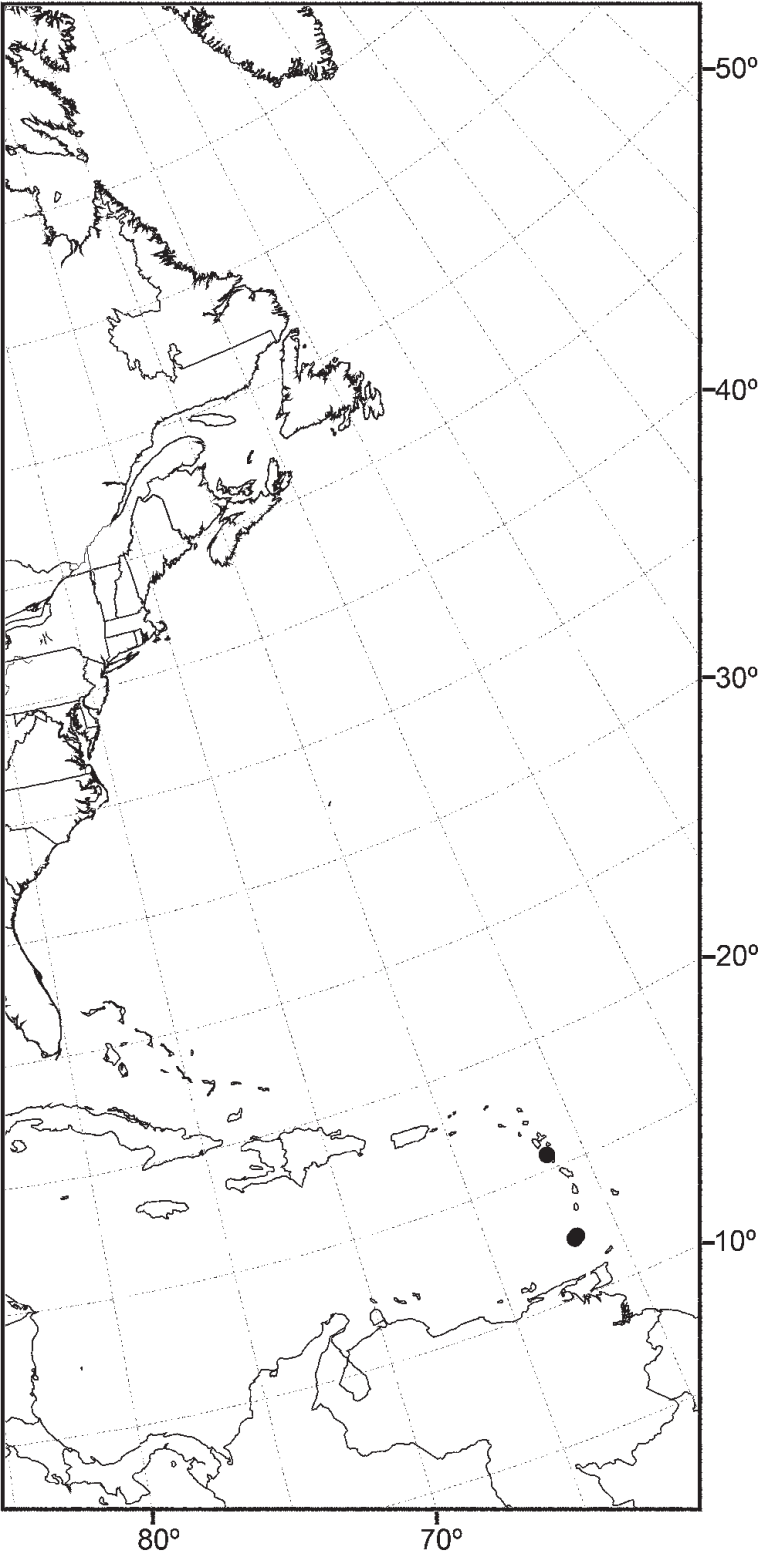
Distribution of *Allotrichoma insulare* sp. n.

(Fig. 90)*Neotropical*: West Indies (Dominica, Grenada).

#### Etymology.

The species epithet, *insulare*, refers to the island habitat of this species in the West Indies.

#### Remarks.

This species is distinguished from congeners, especially those in *Neotrichoma*, by the mostly dark brown mesonotum with faint grayish longitudinal stripes laterad of acrostichal setulae and a subshiny to shiny, blackish brown abdomen that is sparsely microtomentose.

## Supplementary Material

XML Treatment for Hecamedini

XML Treatment for Allotrichoma

XML Treatment for Allotrichoma

XML Treatment for Allotrichoma (Allotrichoma) bezzii

XML Treatment for Allotrichoma (Allotrichoma) bifurcatum

XML Treatment for Allotrichoma (Allotrichoma) deonieri

XML Treatment for Allotrichoma (Allotrichoma) dynatum

XML Treatment for Allotrichoma (Allotrichoma) lacteum

XML Treatment for Allotrichoma (Allotrichoma) lasiocercum

XML Treatment for Allotrichoma (Allotrichoma) occidentale

XML Treatment for Allotrichoma (Allotrichoma) robustum

XML Treatment for Allotrichoma (Allotrichoma) sabroskyi

XML Treatment for Allotrichoma (Allotrichoma) schumanni

XML Treatment for Allotrichoma (Allotrichoma) simplex

XML Treatment for Allotrichoma (Allotrichoma) wallowa

XML Treatment for Neotrichoma

XML Treatment for Allotrichoma (Neotrichoma) atrilabre

XML Treatment for Allotrichoma (Neotrichoma) baliops

XML Treatment for Allotrichoma (Neotrichoma) insulare
